# Alliance for clinical trials in oncology (ALLIANCE) trial A021501: preoperative extended chemotherapy vs. chemotherapy plus hypofractionated radiation therapy for borderline resectable adenocarcinoma of the head of the pancreas

**DOI:** 10.1186/s12885-017-3441-z

**Published:** 2017-07-27

**Authors:** Matthew H. G. Katz, Fang-Shu Ou, Joseph M. Herman, Syed A. Ahmad, Brian Wolpin, Robert Marsh, Spencer Behr, Qian Shi, Michael Chuong, Lawrence H. Schwartz, Wendy Frankel, Eric Collisson, Eugene J. Koay, JoLeen M. Hubbard, James L. Leenstra, Jeffrey Meyerhardt, Eileen O’Reilly, for the Alliance for Clinical Trials on Oncology

**Affiliations:** 1 The University of Texas MD Anderson Cancer Center, University of Texas, 1400 Pressler Street FCT 17.6058, Unit #1484, Houston, TX 77030-4009 USA; 2 Alliance Statistics and Data Center, Mayo Clinic, Rochester, MN USA; 3 University of Cincinnati, Cincinnati, OH USA; 4 Dana-Farber Cancer Institute and Harvard Medical School, Boston, MA USA; 5 NorthShore Evanston Hospital, Evanston, IL USA; 6 The University of California at San Francisco, San Francisco, CA USA; 7 University of Maryland/Greenebaum Cancer Center, Baltimore, MD USA; 8 New York-Presbyterian Hospital/Columbia University Medical Center, New York, NY USA; 9 The Ohio State University, Columbus, OH USA; 10 Mayo Clinic, Rochester, MN USA; 11 Memorial Sloan Kettering Cancer Center, New York, NY USA

**Keywords:** Pancreatic cancer, Borderline resectable, Pancreatoduodenectomy, Radiation, Stereotactic, Chemotherapy, Clinical trial

## Abstract

**Background:**

Borderline resectable pancreatic cancers infiltrate into adjacent vascular structures to an extent that makes an R0 resection unlikely when pancreatectomy is performed de novo. In a pilot study, Alliance for Clinical Trials in Oncology Trial A021101, the median survival of patients who received chemotherapy and radiation prior to anticipated pancreatectomy was 22 months, and 64% of operations achieved an R0 resection. However, the individual contributions of preoperative chemotherapy and radiation therapy to therapeutic outcome remain poorly defined.

**Methods:**

In Alliance for Clinical Oncology Trial A021501, a recently activated randomized phase II trial, patients (*N* = 134) with a CT or MRI showing a biopsy-confirmed pancreatic ductal adenocarcinoma that meets centrally-reviewed anatomic criteria for borderline resectable disease will be randomized to receive either 8 cycles of modified FOLFIRINOX (oxaliplatin 85 mg/m^2^, irinotecan 180 mg/m^2^, leucovorin 400 mg/m^2^ and infusional 5-fluorouracil 2400 mg/m^2^ over 2 days for 4 cycles) or to 7 cycles of modified FOLFIRINOX followed by stereotactic body radiation therapy (33–40 Gy in 5 fractions). Patients without evidence of disease progression following preoperative therapy will undergo pancreatectomy and will subsequently receive 4 cycles of postoperative modified FOLFOX6 (oxaliplatin 85 mg/m^2^, leucovorin 400 mg/m^2^, bolus 5-fluorouracil 400 mg/m^2^, and infusional 5-fluorouracil 2400 mg/m^2^ over 2 days for 4 cycles). The primary endpoint is the 18-month overall survival rate of patients enrolled into each of the two treatment arms. An interim analysis of the R0 resection rate within each arm will be conducted to assess treatment futility after accrual of 30 patients. Secondary endpoints include rates of margin-negative resection and event-free survival. The primary analysis will compare the 18-month overall survival rate of each arm to a historical control rate of 50%. The trial is activated nationwide and eligible to be opened for accrual at any National Clinical Trials Network cooperative group member site.

**Discussion:**

This study will help define standard preoperative treatment regimens for borderline resectable pancreatic cancer and position the superior arm for further evaluation in future phase III trials.

**Trial registration:**

ClinicalTrials.gov: NCT02839343, registered July 14, 2016.

**Electronic supplementary material:**

The online version of this article (doi:10.1186/s12885-017-3441-z) contains supplementary material, which is available to authorized users.

## Background

Borderline resectable pancreatic ductal adenocarcinomas (PDAC), like locally advanced PDACs, infiltrate adjacent tissues to involve adjacent mesenteric vascular structures but the extent to which they do so is relatively minimal so complete macroscopic resection is technically feasible [1]. Nonetheless, their pattern of infiltration is more extensive than that of potentially resectable cancers so the potential for microscopically incomplete (R1) operations is significant when surgery is used as primary therapy [2, 3]. Given that complete microscopic (R0) resection represents a requisite component of curative treatment for patients with PDAC, preoperative treatment regimens designed to both optimize surgical outcomes and to select appropriate patients for pancreatectomy are increasingly being administered to patients with borderline resectable cancers in an attempt to maximize the likelihood for long-term survival.

The therapeutic strategy utilized most frequently for borderline resectable PDAC is currently based entirely upon consensus [4, 5]. Typically, patients first receive 2 to 4 months of systemic chemotherapy followed by a conventionally fractionated course of radiation therapy given over 5–6 weeks with a radiosensitizing fluoropyrimidine or gemcitabine. Patients who complete this multimodality regimen without evidence of disease progression undergo pancreatectomy with curative intent; those who progress systemically prior to the intended operation are treated with non-surgical palliative therapies. This approach leverages theoretical benefits associated with systemic chemotherapy (e.g., systemic antitumor activity for micrometastatic disease known to exist in almost all patients), radiation therapy (e.g., “sterilization” of surgical margins) and time (e.g., patient selection) in an attempt to maximize systemic control, local control, and ultimately, overall survival. Success with this strategy has historically been described primarily in single-institution, retrospective reports [6]. In Alliance for Clinical Oncology Trial A021101, a recently-published, prospective pilot trial, we showed that this paradigm of multimodality therapy can also be implemented effectively in a multi-institutional setting; among 22 patients with borderline resectable PDAC who initiated treatment, 15 (68%) underwent pancreatectomy and the overall survival rate of all accrued patients at 18 months was 50% [7].

This paradigm of sequential administration of chemotherapy and chemoradiation was proposed at a time when gemcitabine—a systemic agent with a response rate in PDAC of less than 10%—was standard therapy [4, 5]. In the “gemcitabine era”, the rationale for following systemic chemotherapy with chemoradiation was the latter’s direct cytotoxic effect on the primary tumor, possibly leading to an improvement in local control or downstaging of the cancer to an extent that might facilitate an R0 resection. However, the added benefit of conventional chemoradiation remains unclear in this setting. Indeed, the sequential administration of gemcitabine-based chemotherapy and chemoradiation led to a radiographic response in only 12% of borderline resectable primary tumors evaluated, and downstaged only 0.8% to technically resectable in one large, retrospective analysis of this approach [8]. Furthermore, conventional chemoradiation as advocated in the consensus guidelines is delivered over a duration of 5–6 weeks prior to a 6 week break before surgery. Because chemoradiation acts principally upon the primary tumor and regional lymph nodes, the systemic micrometastatic disease that appears to exist in all patients with localized PDAC may therefore be suboptimally treated—or untreated—for as many as 3 to 4 months prior to resection using this strategy. Further, given the proximity of the adjacent dose-limiting stomach and small bowel, the delivery of high doses of radiation therapy to the tumor has been prohibited using conventionally fractionated regimens.

The consensus treatment algorithm of systemic chemotherapy followed by conventional chemoradiation that is now frequently used for patients with borderline resectable PDAC requires rigorous review in the context of significant recent advances in both medical and radiation oncology. The systemic regimens now in routine use for patients with metastatic pancreatic cancer—FOLFIRINOX and gemcitabine plus nab-paclitaxel—are associated with objective response rates of 32% and 23%, respectively, in the setting of metastatic PDAC [9, 10]. Given the relatively high response rates associated with these regimens compared to gemcitabine alone, it is conceivable that current systemic therapy may allow better treatment of systemic disease, which represents the key source of morbidity and mortality among patients with PDAC, without forgoing the potential local benefits of chemoradiation. The administration of chemotherapy alone in the preoperative setting for patients with both resectable and borderline resectable cancers is not without precedent: in a single-center study of patients with resectable PDAC, 27 (71%) of 38 patients who received preoperative gemcitabine and oxaliplatin underwent surgical resection and the median OS for all 38 patients was 27.2 months [11]. A recent multi-institutional pilot study of perioperative modified FOLFIRINOX (mFOLFIRINOX) in 21 patients with resectable pancreatic cancer resulted in a 94% R0 resection rate in 17/21 able to undergo resection, and a median OS of 33.4 months [12]. And, in a retrospective evaluation of 64 patients with borderline resectable disease treated with preoperative gemcitabine/docetaxel and other systemic agents, 31 (48%) were resected and the median OS of all 64 patients was 23.6 months [13].

Furthermore, to the extent that radiation therapy may have a role in “sterilizing” the surgical margins of borderline resectable tumors and thereby enhancing rates of R0 resection and local control, new, attractive alternatives such as stereotactic body radiation therapy (SBRT, 6.6 Gy × 5) use a dosing schedule of only 5 days. SBRT, using newer radiation machines with image guidance, fiducial markers, and motion management techniques, has facilitated the delivery of higher doses to the tumor and the reduction of toxicity to normal tissues. In a multicenter prospective study, gemcitabine followed by SBRT was shown to be safe in patients with locally advanced PDAC, with comparable quality of life and reduced abdominal pain [14, 15]. Although SBRT has not been prospectively tested in patients with borderline resectable PDAC, retrospective data are promising and support prospective evaluation [16]. Importantly, SBRT is recommended only to patients who are able to meet specific dose constraints, who can undergo placement of fiducial markers for targeting with daily image guidance, and for whom techniques to sufficiently reduce respiratory tumor motion to <5 mm are available. For patients who do not meet these criteria, hypofractionated image guided radiation therapy (HIGRT, 5 Gy × 5) is an alternate strategy in which a lower yet still potentially effective dose is delivered.

A prospective trial to further the understanding of the relative contributions of preoperative systemic and local therapies for patients with borderline resectable PDAC is now timely and essential to further progress. A021501, a collaborative randomized phase II study, will establish a treatment strategy to which novel regimens can be compared in the future. This study will compare the efficacy of two preoperative regimens, one of which utilizes modern systemic therapy alone and one that uses systemic therapy and hypofractionated SBRT or HIGRT.

## Methods

### Objectives

**Fig. 1:**
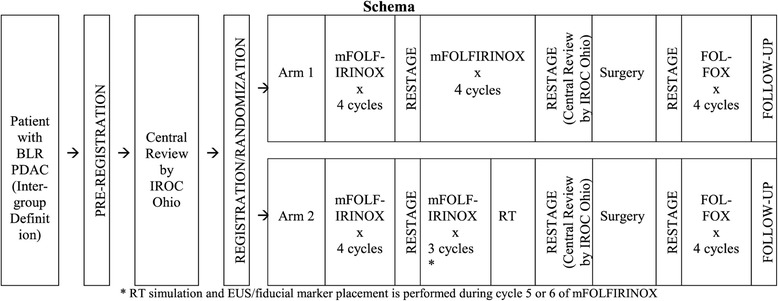
Study calendar

The primary objective of this study is to evaluate and estimate the 18-month overall survival (OS) rate of patients with borderline resectable PDAC receiving preoperative therapy consisting of one of the following regimens prior to intended surgical resection and postoperative therapy with 4 cycles of modified FOLFOX6 (mFOLFOX6): 1) Arm 1: 8 cycles of systemic mFOLFIRINOX, or 2) Arm 2: 7 cycles of systemic mFOLFIRINOX followed by hypofractionated radiation therapy. Secondary objectives include: 1) to evaluate and estimate the R0 resection rates in patients receiving each of the two multimodality treatment regimens, 2) to evaluate and estimate the event-free survival in patients receiving each of the two multimodality treatment regimens, 3) to evaluate and estimate the pathologic compete response (pCR) rates in patients receiving each of the two multimodality treatment regimens, and 4) to assess the adverse events (AE) profile and safety of each treatment arm, using the Common Terminology Criteria for Adverse Events (CTCAE) and the Patient Reported Outcome version of the CTCAE (PRO-CTCAE). The study schema is illustrated in Fig. 1. The study calendar is illustrated in Table 1.

**Table 1:** Study calendar. Pre-Study Testing Intervals. To be completed ≤21 DAYS before registration: All laboratory studies, history and physical. To be completed ≤28 DAYS before registration: CT/MRI scans used for staging

	Prior to Pre-Reg	Prior to Reg*	Day 1 of each cycle of mFOLFIR-INOX*	RT (day 1 to day 5) (Arm 2 only)	Surgery	Day 1 of each cycle of FOLFOX *	Post Tx Follow- up**
Tests & Observations							
History and physical, weight, PS ***		X [1]	X [1]	X [1]		X [1]	X [1]
Pulse, BP		X [1]	X [1]	X [1]		X [1]	X [1]
Height		X [1]					
Adverse Event Assessment (CTCAE) †		X [1]	X [1]	X [1]	D [1]	X [1]	
Adverse Event Assessment (PRO-CTCAE) ††		X [2]	X	X	D	X	
Registration QOL/Mental Well-being/Physical Well-being/Fatigue		X [2]					
Laboratory Studies							
CBC, Differential, Platelets		X	X	X	X	X	X
Chemistry (Serum Creatinine, Electrolytes, AST, ALT, Alk. Phos., Albumin, Total Bilirubin)		X	X	X	X	X	X
Pregnancy Test (#)		X					
CA 19–9		A	A	A	A	A	X
RT Planning							
EUS with Fiducial Placement for RT				B			
Staging							
Staging CT Scan of Chest or Chest X-ray/CT or MRI of Abdomen	X [3]		C [3]	C [3]	C [3]	C [3]	X [3]
Central Radiographic Review		C			C		
Central Pathology Review					X [4]		
Optional Correlative studies: For patients who consent to participate							
Blood specimen sample (A021501-PP1)	Between Registration and C1D1. See Additional file 1: Section 6.2.						
Imaging (A021501-IM1)	See Additional file 1: Section 6.4.2 for CT images submission time points and requirements.						

*Labs completed prior to registration may be used for day 1 of cycle 1 if obtained ≤7 days prior to treatment (except pregnancy test and CA 19–9, as detailed below). For subsequent cycles, labs, tests and observations may be obtained +/− 3 days from scheduled day of assessment. Radiographic windows are +/− 7 days from scheduled day of assessment

**After off-treatment (evaluation of the last treatment cycle), patients will have physical examinations, labs, and staging scans every 16 weeks (+/− 28 days) until they have reached 24 months post-registration or until documented progression, whichever occurs first. Thereafter, survival information is required every 6 months for 5 years post-registration. For patients who discontinue treatment for progressive disease or are removed from protocol treatment, survival information is required every 6 months for 5 years post-registration. See Additional file 1: Section 12.0 for removal of patients from protocol therapy

***Drug dosages need not be changed unless the calculated dose changes by ≥10%

1 May be performed by physician, NP, or PA responsible for oncologic care of the patient

2 To be completed after registration and ≤21 days prior to treatment. See Additional file 1: Appendix I

3 Chest scans must be CT or chest X-ray. Abdominal baseline and restaging scans can include either a CT or MRI, although CT is preferred. The same method of scanning used at baseline must be used for all subsequent evaluations. The CT must be acquired with 3 mm or less slice thickness. See Additional file 1: Section 7.4.1 for further details. Supporting documentation is to be submitted, per Additional file 1: Section 6.1.1. The baseline scan and restaging scan after completion of preoperative therapy/prior to surgery are to be centrally reviewed by the Alliance ICL at IROC Ohio, per Additional file 1: Section 7.4.2

4 Central Pathology review is retrospective. Sites must submit slides within 60 days of surgery of patient. See Additional file 1: Section 6.2

†Solicited AEs are to be collected starting at baseline. Routine AEs are to be collected starting after registration. See Additional file 1: Section 9.1 for the list of solicited AEs. See Additional file 1: Section 9.4 for expedited reporting of SAEs. See Additional file 1: Section 9.2 for reporting of surgical AEs, to be completed within 90 days after surgery

††Patients complete PRO-CTCAE by paper booklet ordered through the CTSU website. See Additional file 1: Section 9.1 for administration instructions. See Additional file 1: Appendix I for PRO-CTCAE assessments for IRB submission and review only. See Additional file 1: Section 4.5 for ordering instructions. PRO-CTCAE booklets should be administered at the following time points: ≤ 21 days prior to treatment; day 1 of each cycle of mFOLFIRINOX (+/− 3 days); RT days 1–5 (Arm 2 only); prior to surgery (+/− 7 days); and day 1 of each cycle of FOLFOX (+/− 3 days)

# For women of childbearing potential (see Additional file 1: Section 3.3.6). Must be done ≤7 days prior to registration

A CA19–9 may be performed <28 days prior to registration. Subsequently, CA 19–9 may be performed +/− 14 days from the scheduled date. During treatment, CA 19–9 should be performed every 28 days. For patients who have normal CA 19–9 levels at baseline, continued testing of CA 19–9 is not required

B EUS/fiducial marker placement is mandatory for patients in the RT arm. Immediately following review of first restaging studies, planning for EUS/fiducial placement and RT simulation should be scheduled to be performed during either cycle 5 or 6 of mFOLFIRINOX

C Restaging scans should be performed for both Arm 1 and Arm 2 at the following time points: 1. After the first 4 cycles of mFOLFIRINOX; 2. Prior to Surgery; 3. Post-surgery but prior to first cycle of FOLFOX; 4. After 4 cycles while on FOLFOX. The baseline and restaging scan after completion of preoperative therapy and prior to surgery are to be centrally reviewed by the Alliance ICL at IROC Ohio per Additional file 1: Section 7.4.2. After protocol treatment, scans should be performed per the schedule indicated by footnote “**”

D Surgery-related AEs should be assessed and captured within 90 days of surgery

### Setting

This study, A021501, is conducted by the Alliance for Clinical Trials in Oncology (Alliance), collaborating with the Southwest Oncology Group (SWOG), NRG Oncology, and the Eastern Cooperative Oncology Group-American College of Radiology Imaging Network (ECOG-ACRIN). Patients will be accrued from member institutions of any of these National Clinical Trials Network cooperative groups. The institutional review board at each participating institution must approve the study. All patients must provide written informed consent.

### Eligibility, preregistration and registration

Registration is accomplished in two phases. Eligibility criteria are confirmed during a preregistration phase and include age ≥ 18 years, ECOG performance status (PS) of 0 or 1, biopsy confirmation of adenocarcinoma of the pancreatic head, no remote lymphadenopathy or distant metastases, and a computed tomography (CT) or magnetic resonance imaging (MRI) study of the abdomen using a pancreatic protocol and CT or MRI of the chest demonstrating a primary tumor characterized by one or more of the following relationships (Intergroup Criteria [6]): 1) a tumor-vessel interface (TVI) with the mesenteric vein (SMV) or portal vein (PV) measuring ≥180° of the circumference of either vein’s wall or short-segment occlusion of either vein with a normal vein above and below the obstruction amenable to reconstruction; 2) any TVI with the common hepatic artery (CHA) with a normal artery proximal and distal to the TVI amenable to reconstruction; and 3) a TVI with the superior mesenteric artery (SMA) measuring <180° of the circumference of the vessel wall. Tumors with an interface with the SMV and PV measuring <180° and without an interface with either the CHA or SMA—considered borderline resectable by other guidelines—are considered resectable [5]. A TVI with the SMV, PV, or CHA but without a normal vessel proximal and distal to the interface to allow reconstruction, a TVI with the SMA measuring ≥180° of that vessel’s circumference, and a TVI with the aorta are considered to represent locally advanced disease. Patients with resectable, locally advanced or metastatic PDAC are ineligible.

Final registration requires real-time confirmation of disease stage with central review of all radiologic images and multidisciplinary evaluation of the patient by a medical oncologist, radiation oncologist, and surgeon. Additional criteria included granulocytes ≥2000/μL, hemoglobin >9 g/dL, platelets ≥100,000/μL, albumin >3.0 g/dL, creatinine ≤1.5 times the upper limit of normal, aspartate transaminase and alanine transaminase ≤2.5 times the upper limit of normal, and bilirubin ≤2 mg/dL. Exclusion criteria include peripheral neuropathy grade ≥ 2, prior therapy for PDAC, Gilbert’s syndrome or homozygosity for UGT1A1*2, and any active second malignancy.

### Treatment plan

#### Preoperative treatment

Upon registration, patients are randomized to one of two treatment arms. Patients in both arm 1 and arm 2 receive 4 cycles of mFOLFIRINOX (bolus intravenous [IV] oxaliplatin 85 mg/m^2^, irinotecan 180 mg/m^2^, leucovorin 400 mg/m^2^, then a 46–48-h IV infusion of 5-fluorouracil 2400 mg/m^2^) with subsequent prophylactic white blood cell growth factor support. Following the first four cycles of mFOLFIRINOX, patients are restaged with CT or MRI, and those without evidence of metastases and who maintain a PS of 0 or 1 receive either an additional 4 cycles of mFOLFIRINOX (arm 1) or an additional 3 cycles of mFOLFIRINOX followed by radiotherapy using either SBRT (6.6 Gy × 5) or HIGRT (5 Gy × 5).

SBRT is strongly preferred over HIGRT and it is expected that the majority of patients eligible for this trial will receive SBRT. Only those patients who do not meet specific criteria as outlined in the protocol should be offered HIGRT to ensure patient safety. SBRT allows for simultaneous integrated boosts (SIB) to the tumor vessel interface (TVI). In contrast, HIGRT delivers a homogeneous dose without a boost to the TVI in order to minimize normal tissue dose. Each site must be credentialed for SBRT prior to enrollment and all radiation plans must be centrally reviewed prior to treatment. Pancreas tumors are not easily seen on cone beam CT scans at the time of SBRT. Placement of fiducial markers under echoendoscopic guidance within or near the tumor is required prior to radiation. An online tutorial for SBRT planning and delivery has been created to assist those sites who have not previously performed pancreas SBRT (*www.educase.com*).

#### Surgical resection

Following completion of either chemotherapy alone (arm 1) or chemotherapy followed by radiation (arm 2), all patients are restaged with CT or MRI, and those without locally advanced or metastatic disease on immediate central radiologic review and who have a PS of 0 or 1 are required to undergo pancreatectomy within 4–10 weeks. Radiographic “downstaging” is not required. The following surgical procedures are mandated: 1) periadventitial dissection of the right lateral aspect of the SMA [17]; 2) venous and/or short-segment hepatic arterial resection when necessary to achieve negative margins; and 3) evaluation of the histopathologic status of the pancreatic and bile duct margins intraoperatively, with re-resection when appropriate.

#### Postoperative treatment

Patients with a PS of 0 or 1, without prohibitive toxicity from previous chemotherapy, and without evidence of residual or recurrent disease on CT/MRI images are considered for 4 cycles of postoperative mFOLFOX6 (oxaliplatin 85 mg/m^2^ and leucovorin 400 mg/m^2^ IV, bolus 5-fluorouracil 400 mg/m^2^ IV, followed by a 46–48-h IV infusion of 5-fluorouracil 2400 mg/m^2^).

### Assessment and follow up

Radiologic response and progression are evaluated using the Response Evaluation Criteria in Solid Tumors (RECIST) version 1.1 guidelines [18]. Analysis of the surgical specimen is performed following recommendations of the American Joint Committee on Cancer and the College of American Pathologists guidelines [19, 20]. Histopathologic response is centrally reviewed and characterized by the extent of residual viable cancer cells in the surgical specimen (<5% or ≥5% cancer cells) [21]. Histopathologic complete response (CR) is defined as the absence of cancer cells in the specimen; in such cases, the pretreatment biopsy is centrally re-reviewed. Resection status is characterized as R0, R1 (microscopic tumor at any margin), or R2 (macroscopically incomplete resection).

Adverse events (AEs) are graded using the Common Toxicity Criteria Adverse Event (CTCAE) Version 4.0 [22]. AEs are recorded during preoperative chemotherapy and chemoradiation, during surgery and within the 90-day postoperative period, and during postoperative therapy.

Patients are followed every 4 months following treatment until 24 months post registration or until documented progression of disease. All visits include a history and physical examination, laboratory studies, and CT or MRI of the chest and abdomen. The appearance of any new lesion with characteristics of local relapse or metastatic disease is considered recurrence. Following 24 months, survival information is required every 6 months for 5 years post-registration.

### Correlative studies

Patients may also be consented to two correlative studies. The first will utilize patients’ germline DNA from a single 10 mL blood sample acquired at registration. This study will test the association between the rs2853564 *VDR* variant and OS, and will discover novel candidate genes associated with both OS and chemotherapeutic toxicity of using genome-wide genotyping approaches [23]. In the second study, standard cross-sectional imaging studies will be used to estimate prognosis based on discrete radiographic features of the primary tumor [24].

### Statistics

#### Sample size

We anticipate enrolling a maximum of 124 evaluable patients (62 per arm) per statistical design. An additional 10 (5 per arm) patients will be accrued to account for cancelations, ineligibilities, major violations, and lost-to-follow up, etc. Thus the total targeted accrual will be 134 patients. With an anticipated accrual of 4 patients per month, the accrual period of the study is estimated at 34 months.

#### Power analysis

Eligible patients will be stratified by performance status and randomized into two treatment arms. Within each arm, a single-arm sequential design with one interim analysis for futility will be implemented. The final efficacy analysis and interim analysis will be based on the 18-month OS rate and R0 resection rate, respectively, and evaluated in each arm separately. The comparison of OS between arms will be carried out only if both arms are deemed promising at the end of the trial. A “pick-the-winner” strategy will be implemented to choose one treatment strategy for recommendation.

The median OS of patients treated with multimodality therapy for borderline resectable PDAC reported in historical studies varies from 14 to 28 months, with a median of 21 to 22 months and an interquartile range of about 18 to 23 months. Most of the previous studies are retrospective single-institution studies subject to patient selection bias, which likely skew the reported OS higher. Thus, we consider the median OS of at most 18 months (equivalent to 18 m OS rate of 0.50, assuming exponential survival function) which is the lower bound of the IQR of literature reported data as the null hypothesis. To demonstrate clinical meaningful improvement in OS, we target an alternative hypothesis of a median OS of at least 27 months (equivalent to 18 m OS rate of 0.63; a 50% increase in median OS time).

A maximum of 62 evaluable patients in each arm will provide 82% power to detect an absolute improvement of 13% in 18 months OS rate, i.e., testing alternative hypothesis that the 18 months OS rate is at least 63% against the null hypothesis that the 18 months OS rate is at most 50%, at a one-sided significance level of 0.07.

One interim analysis will be conducted to assess treatment futility for each arm separately. For each treatment arm, the R0 resection rate will be evaluated when surgical data become available for the first 30 evaluable patients. If the R0 resection rate is at least 60% (null hypothesis), based on weighted R0 resection rate from historical data, the arm warrants continuing to full accrual for OS final analysis. If the observed R0 resection rate is significantly less than 60%, futility of that arm has been met and accrual of new patients to that arm will cease. Therefore, the interim analysis based on the R0 resection rate will be testing the alternative hypothesis of R0 resection rate ≤ 40% against the null hypothesis of R0 resection rate ≥ 60%. The probability of stopping accrual at interim is 43% and 0.8% if the true R0 resection rate is 40% and 60%, respectively.

## Discussion

Herein we describe a multi-center randomized phase II clinical trial to be conducted in the US National Clinical Trials Network designed to evaluate the efficacy of two rational preoperative treatment strategies for patients with borderline resectable PDAC. This study, A021501, builds upon our previous work, A021101, and it will define standard preoperative treatment regimens to which future novel regimens can and will be compared in subsequent randomized trials.

The statistical design of this trial focuses on evaluating whether clinical meaningful improvement in survival can be achieved by the tested regimens compared to historical data. This study uses a critically important interim surrogate endpoint (R0 resection rate) that is different from the final endpoint (18 month OS rate) and an interim analysis which allows elimination of a futile arm after only 30 patients are enrolled to that arm. If both arms are deemed promising at the end of the trial, a pick-the-winner strategy will be employed to choose one arm as the “winner”. Use of a proximate end point, the R0 resection rate, for the interim analysis protects patients from the possibility of being randomized to a futile arm while the whole study is still powered to detect a clinically important and meaningful improvement in the OS rate.

The concept and design of this trial was developed and evolved by a multi-disciplinary team within the Alliance for Clinical Trials in Oncology with the support and critical feedback of the National Cancer Institute’s Pancreatic Cancer Task Force and members of the other participating cooperative groups. The trial represents a natural next step in an investigational program that has proceeded with the advice, investment, and involvement of the Cancer Therapy Evaluation Program (CTEP). Indeed, at the request of CTEP, we performed an initial pilot study A021101 designed to rapidly assess the feasibility of a multi-institutional study of borderline resectable PDAC and to develop a standardized clinical and research infrastructure (e.g., rapid review of imaging, pathologic assessments, quality control of radiation protocols) specific to this disease stage that is necessary to study it. A021101 represented one of the most collaborative studies that has ever been conducted in the pancreas cancer space: it met its accrual endpoint early and within a year due to the enthusiasm of centers within the Alliance, ECOG-ACRIN, SWOG and NRG Oncology [7]. The trial demonstrated that rapid, real-time central review of imaging is feasible, established other infrastructural elements viewed as critical to the conduct of multimodality treatment trials for pancreatic cancer, and has set the stage for this subsequent randomized phase II A021501 study which represents the natural next step in this line of investigation.

## Additional file

Additional file 1: Study protocol. (DOCX 15460 kb)
