# Supplementary material for: Alliance for clinical trials in oncology (ALLIANCE) trial A021501: preoperative extended chemotherapy vs. chemotherapy plus hypofractionated radiation therapy for borderline resectable adenocarcinoma of the head of the pancreas
Source: BMC Cancer. 2017 Jul 27;17:505. doi: 10.1186/s12885-017-3441-z (PMC5530569; doi:10.1186/s12885-017-3441-z)
Supplement: Additional file 1: — Study protocol. (DOCX 15460 kb) [file 12885_2017_3441_MOESM1_ESM.docx]

Protocol Update #01

11/01/2016

**Alliance for Clinical Trials in Oncology**

**_________________________________________**

**PROTOCOL UPDATE TO ALLIANCE A021501**

**_________________________________________**

**Preoperative Extended Chemotherapy vs. Chemotherapy Plus Hypofractionated Radiation Therapy for Borderline Resectable Adenocarcinoma of the Head of the Pancreas**

| X | **Update:** |  |  | **Status Change:** |  |
| --- | --- | --- | --- | --- | --- |
|  |  |  |  |  |  |
|  | Eligibility changes |  |  | Activation |  |
|  |  |  |  |  |  |
| X | Therapy / Dose Modifications / Study Calendar changes |  |  | Closure |  |
|  |  |  |  |  |  |
| X | Informed Consent changes |  |  | Suspension / temporary closure |  |
|  |  |  |  |  |  |
| X | Scientific / Statistical Considerations changes |  |  | Reactivation |  |
|  |  |  |  |  |  |
| X | Data Submission / Forms changes |  |  |  |  |
|  |  |  |  |  |  |
| X | Editorial / Administrative changes |  |  |  |  |
|  |  |  |  |  |  |
|  | Other: | | | |  |

***Expedited review is allowed. IRB approval (or disapproval) is required within 90 days. Please follow your IRB of record guidelines.***

**UPDATES TO THE PROTOCOL:**

[**Cover Page**](#CoverPage)

- Below the study title, after “Study-specific credentialing requirements,” the statement, “Not a CIRB-reviewed study” has been added.
- The ClinicalTrials.gov NCT identifying number has been added.
- A new Imaging Co-Chair, Dr. Koay, has been added.
- The contact information for the Radiation Oncology Co-Chairs, Dr. Chuong and Dr. Herman have been updated.
- The SWOG Co-Chair has been corrected from Dr. Ahmad Syed to Dr. Syed Ahmad.
- The email address for Dr. Jeffrey Meyerhardt has been updated.
- The QOL Statistician has been updated from Jeff Sloan to Amylou Dueck.
- The Protocol Coordinator has been updated from Morgen Alexander-Young to Alexandra LeVasseur.

[**CTSU Information**](#CTSU)

Contact information for CTSU and section headers have been updated to be consistent with the CTSU boilerplate language.

[**Schema**](#Schema)

In the first paragraph under the schema diagram, the following changes have been made:

- The phrase “radiation therapy” has been replaced with “imaging” to correctly reflect study requirements.
- A new second sentence has been added: “Radiation therapy must be performed at a facility participating in the IROC monitoring program.”

[**Section 1.3**](#Sec1p3)

In the second paragraph, the following phrase “and published on the JAMA surgery website…” has been added. A citation for this publication has also been added as #17 in the references section ([Section 16.0](#Sec16p0)). All subsequent citations have been renumbered.

[**Section 1.4.6**](#Sec1p4p6)

In the second to last sentence that begins, “However since the dose…,” the number “9” has been replaced with the number ‘8” to correct an error.

[**Section 2.2.4**](#Sec2p2p4)

The phrase “using the CTC-AE and PRO-CTCAE” has been added to the end of the objective in [Section 2.2.4](#Sec2p2p4).

[**Section 2.3**](#Sec2p3)

The baseline physical and mental well-being items have been moved to [Objective 2.3](#Sec2p3) as follows: “…covariates of baseline overall QOL, mental well-being, physical well-being, and fatigue.”

[**Section 2.4**](#Sec2p4)

- The QOL objective, [Section 2.4](#Sec2p4), has been removed. Subsequent objectives have been renumbered.
- Any reference to time points other than baseline for LASA items have been removed.

[**Section 2.5.1**](#Sec2p4)

The former [objective 2.5.1](#Sec2p4) has been removed, as the ST-1 correlative research has been removed from the study.

[**Section 4.3**](#Sec4p3)

- A021501 will not be CIRB reviewed. Under, “IRB Approval,” the following statement has been added: “Please note, this study is not reviewed by the NCI Central Institution Review Board (CIRB).”
- The CTSU Language has been updated with the new language provided by the CTSU.

[**Section 4.3.1**](#Sec4p3p1)

The following new third bullet point has been inserted between the previous second and third bullet points:

- Either enter the protocol # in the search field at the top of the protocol tree, or

[**Section 4.3.2**](#Sec4p3p2)

The first two bullet points have been removed and replaced with the following text:

- CTSU Transmittal Sheet (optional)
- IRB Approval

[**Section 4.3.3**](#Sec4p3p3)

The following changes have been made:

- A new first paragraph has been inserted (previously “Check the status…”).
- A new final paragraph has been inserted that begins: “Note: The status…”

[**Section 4.3.4**](#Sec4p3p4)

All text in this section has been replaced with updated submission information.

[**Section 4.5**](#Sec4p5)

Instructions have been added for ordering patient booklets.

[**Section 4.7.1**](#Sec4p7p1)

Instructions for registering to the biospecimen correlative substudy, A021501-ST1, have been removed.

[**Section 5.0**](#Sec5p0)

Within the Study Calendar, the following changes have been made:

- The specimens submitted per correlative study A021501-ST1 have been removed
- The word “Total” has been added before “Bilirubin” to more accurately describe the laboratory study.
- Adverse Event Assessment (PRO-CTCAE) and Registration QOL… have been moved to immediately below Adverse Event Assessment (CTCAE) under the “Tests & Observations” heading in response to comments from the NCI Reviewers, as these measures will now be integrated with AE reporting.
- All time points other than baseline have been removed for LASA items.

Within the footnotes under the Study Calendar, the following changes have been made:

- The first sentence of footnote ** has been removed, as data collection at 4 weeks post-therapy is no longer required.
- In the new first sentence of footnotes **, previously the second sentence, the following changes have been made:
  - “At completion of therapy” has been replaced with “After off-treatment (evaluation of the last treatment cycle) to reduce possible time point confusion
  - The phrase “physical examinations” has replaced “visits”.
- Footnote 4 has been moved to become footnote 2. Subsequent footnotes have been renumbered.
- A new footnote, ††, has been added to provide additional information about PRO-CTCAE booklets and administration time points.
- Footnote D has been corrected to remove the window of 60 to 90 days. Instead, surgery-related AEs should be assessed and captured within 90 days of surgery.
- Footnotes have been renumbered to reflect changes made within the Study Calendar.
- Footnote D has been added to PRO-CTCAE.

[**Section 6.2**](#Sec6p2)

- Above the specimen submission table, the following changes have been made:
  - References to study A021501-ST1 have been removed. Reference to the specimen collection kits for the correlative research have been removed.
  - The final sentence of the second paragraph has been moved to create a third paragraph, and the phrase “tissue and blood” in that sentence has been replaced with the word “specimens”, to reduce any site confusion.
- Within the specimen submission table, the following changes have been made:
  - The previous specimens to be submitted for patients who consent to A021501-ST1 have been removed.
  - Two new title rows have been added: “Mandatory for all patients on A021501,” and “To be submitted ONLY for patients that consent to A021501-PP1***.”
- The previous footnotes **, ***, and † have been removed, as they pertained to the ST1 study.
- A new footnote ** has been added: “**Required ONLY in patients with pathologic CR or histology other than adenocarcinoma at time of surgery. See [Section 6.2.2](#Sec6p2p2) for submission requirements.”
- A new footnote *** has been added: “***Patients who consent to A021501-PP1 are those who answer “Yes” to the model consent question, “I agree to have my blood specimen collected…”
- The previous footnotes 2 and 3 have been removed (previously, “Blocks/cores to be used for biomarker genomic analyses…” and, “Plasma to be used for the biomarker analyses described in previous [Section 14.1](#Sec14p1).”

[**Section 6.2.2**](#Sec6p2p2)

- In the current fourth from last paragraph, reference to A021501-ST1 has been removed, and the informed consent question for specimen banking has been added for clarity.
- The previous fourth from last paragraph of [Section 6.2.2](#Sec6p2p2) has been removed (previously starting with, “For patients who consent to participate [model consent question “I agree to have my specimen collected…]”).

[**Section 6.2.3**](#Sec6p2p3)

The previous [Section 6.2.3](#Sec6p2p3) has been removed (previously, “Tissue submission requirements for A021501-ST1”), as the correlative substudy A021501-ST1 has been removed from the protocol. Subsequent sections have been renumbered.

[**Section 6.2.4**](#Sec6p2p3)

The previous [Section 6.2.4](#Sec6p2p3) has been removed (previously, “Blood specimen submission for A021501-ST1”), as the correlative substudy A021501-ST1 has been removed from the protocol.

[**Section 6.3**](#Sec6p3)

This entire section has been removed as these items are included in the [Section 5.0](#Sec5p0) Study Calendar. Subsequent sections have been renumbered.

[**Section 7.0**](#Sec7p0)

The phrase “associated with the enrolling institution and” has been removed from the fourth paragraph to reduce confusion on radiation therapy requirements.

[**Section 7.2**](#Sec7p2)

- Instructions on how to access online EduCase tutorial videos for radiation planning and treatment have been added to create a new first paragraph.
- Under the heading, “SBRT is strongly preferred...,” the following changes have been made for clarity:
  - In the first bullet point, “prior to delivery of radiation therapy on any protocol patient,” has been removed.
  - In the second bullet point, “(no active bleeding or symptoms),” has been added to the second sentence after the word “resolved.”
  - In the third bullet point, the phrase, “based on endoscopy,” has been added after the word “stomach.”
  - A new fifth bullet point has been added that begins, “Patients should have 4D CT…”
  - The final bullet point has been removed (previously, “SBRT is recommended…”).
- Under the heading, “HIGRT should be considered…,” the following changes have been made for clarity:
  - In the 2^nd^ bullet, the phrase, “on endoscopy,” has replaced the phrase “(loss of fat plane).”
  - The 4^th^ and 5^th^ bullets have been removed (previously both beginning with “HIGRT”).

[**Section 7.2.1**](#Sec7p2p1)

- In the heading, “Fiducial marker placement…,” the word attempted has replaced the word performed.
- Under the heading, “Fiducial marker placement…,” the following changes have been made:
  - In the first sentence of the second bullet point, the words “in or” have been added before the word “directly”.
  - A new final bullet point has been added that begins: “In the rare…”
- In the “CT simulation…” section header, the words “All patients must undergo,” have been removed, and the words “is done” have been added for clarity.
- Under the heading, “CT simulation…,” the following changes have been made:
  - In the second bullet, the phrase “arms above the head,” has been added after the word “supine.”
  - In the second to last bullet, the phrase, “in any direction,” has been added to the third sentence after the words “tumor motion,” and a new final sentence has been added that begins: “If ≤ 5 mm of tumor motion…”
  - In the last bullet, the phrase, “or PET,” has been added after “MRI.”

[**Section 7.2.1.1**](#Sec7p2p1p1)

- In the section header, the text in parenthesis has been removed (previously, “(Please see the IROC…)”).
- A new first paragraph has been added that begins: “See tutorial for examples…”
- Within the HIGRT/SBRT treatment planning section, the following changes have been made for clarity:
  - Within Dose Prescription, the following changes have been made:
    - In the first bullet, the phrase “or PTV” has been removed from the end of the sentence, and a new second sentence has been added.
  - Within Target Volumes, the following changes have been made:
    - A new subsection, “Target Volumes Definitions,” and subsequent sub-bullet points have been added for clarity.
    - Under Gross Tumor Volume (GTV), in the second bullet, the word “only” has been added to the end of the sentence.
  - The “Gross Internal Target Volume (GITV),” section has been moved to immediately follow the “Target Volumes” section.
  - Within Gross Internal Target Volume (GITV), the following has been changed:
    - In the first bullet, the phrase, “For patients who have 4D CT simulation,” has been added as a new beginning to the sentence.
    - A new second bullet has been added that begins: “GITV will not be…”
    - The third bullet has been replaced with a new third bullet and two sub-bullet points that begins: “The GITV may be determined by either…”
  - The “Tumor-Vessel Interface (TVI),” section has been moved to immediately follow the “Gross Internal Target Volume (GITV)” section.
  - Within Tumor-Vessel Interface (TVI), the following has been changed:
    - In the second bullet, all abbreviations have been spelled out, and an additional location, “common hepatic artery (CHA),” has been added.
    - A new third bullet has been added that begins: “TVI structures will…”
    - The fourth bullet has been removed (previously, “The entire circumference…).
    - In the second to last bullet, the following changes have been made:
      - The words “these contours” have replaced the words “this contour”
      - The words “in the axial, coronal, and sagittal view” have been added.
    - In the last bullet point, the following changes have been made:
      - The phrase, “This contour,” has been replaced with a phrase that begins: “The vessel(s) contoured at the level…”
      - The word “expansion” has been added as a final word in the sentence.
  - Three new bullets have been added for Fiducials/Clips: “Fiducials and/or clips should be contoured…,” and “This volume should be expanded 3 mm…,” and “Note: If insurance does not cover…”
  - Within Planning Target Volume (PTV), the following changes have been made:
    - In the HIGRT bullet point, the following changes have been made:
      - The word “modified” has been added to the first sentence.
      - The phrase, “modified PTV = GITV + TVI + 3-5 mm),” has been added as a final sentence.
    - Within the SBRT bullet point, the following changes have been made:
      - The second and third sentences have been removed.
      - The phrase, “(PTV = GITV + TVI + 3 mm),” has been added as a final sentence for the bullet.
      - A sub-bullet point that begins, “The OARs…” has been added.
    - The third bullet point has been replaced with text that begins, “The final modified…” (previously, “Fiducial markers used…”).
  - Within Treatment Technique, in the SBRT bullet point, the phrase, “static IMRT or VMAT must be used; 3DRT is not permissible,” has replaced the phrase that began, “intensity modulated radiation…”
  - Within Motion Management, the first bullet point has been moved to create the final bullet point of the section that begins: “SBRT: Cranial caudal tumor…”
  - Within Organs at Risk (OARs), the following has been added:
    - “Follow the upper abdominal contouring atlas located:https://www.rtog.org/CoreLab/ContouringAtlases/UpperAbdominalNormalOrganContouringConsensusGuidelines.aspx.”
    - In the first bullet, “Spleen should also be contoured and added as an OAR, although no formal dose constraint will be used for spleen.”
    - A new third bullet: “OAR dose constraints are based on those OARs within 1 cm (all directions of the final modified PTV).”
    - A new fourth bullet: “‘Proximal’ refers to any OAR within 1 cm of the final modified PTV (mPTV).”
    - A new fifth bullet, “HIGRT constraints,” and two subsequent sub-bullet points, “Modified PTV…,” and “A dose of 5 Gy….”
    - In the sub-bullet points under “SBRT constraints,” the following changes have been made:
      - New first, second, and third sub-bullet points have been added that begin, “Modified PTV=…,” and “PTV=GTV…,” and “Greater than 95%...”
      - In the fourth sub-bullet, the following changes have been made:
        - In the first sentence, “90” has been corrected to “95.”
        - The words “modified” and “(mPTV)” have been added to the first sentence.
        - A new final sentence has been added that begins: “Efforts should be made to focus…”
      - In the fifth sub-bullet, the phrase, “at least,” has been added before the phrase, “15 Gy and 20 Gy.”
  - A new title has been added for the table following the Organs at Risk (OARs) section that reads: “SBRT Required OARs and Constraints.”
  - Within the “SBRT Required OARs and Constraints” table, the following changes have been made:
    - In the second row, “Modified PTV” has replaced “mPTV” in the first column, a “m” has been added in the second column, and the constraints in the third column now read “V25 > 95% (range 25-40 Gy),” (previously, “V33 > 95%”).
    - In the third row, text has been added in all three columns for “PTV.”
    - In the fifth row, the words “Proximal” and “Prox” have been removed (previously, “Proximal Duodenum” and Duodenum Prox”).
    - A new seventh row has been added, and the third column now includes the phrase, “V33 <1cc.”
    - In the eighth row, the word “Small” has been added.
    - A new tenth row has been added, and the third column now includes the phrase, “V33 <1cc.”
    - The former ninth row has been deleted (previously, “Proximal Bowel”).
    - In the thirteenth row, “V33 <1cc,” has replaced, “V12 <50%.”
    - The former thirteenth row has been deleted (previously, “Proximal Stomach”).
    - In the sixteenth row, “V20 <1cc,” has replaced, “V8 <1cc,” in the third column.
    - A new final row titled “Spleen,” has been added to both SBRT and HIGRT constraints tables.
  - The phrase “Required OARs and” has been added to the title of the second table, so that it now reads: “HIGRT Required OARs and Constraints.”
  - Within the “HIGRT Required OARs and Constraints” table, the following changes have been made:
    - In the second row, “Modified PTV” has replaced “mPTV” in the first column, a “m” has been added in the second column, and “Rx dose is 5 Gy x 5” has been added to the beginning of the constraints in the third column, along with a “m” in front of “PTV.”

[**Section 7.2.2.4**](#Sec7p2p2p4)

The contact information for Drs. Herman and Chuong has been updated.

[**Section 7.4.1**](#Sec7p4p1)

The following sentence has been added as a second paragraph:

- “Sites are to use their optimal preoperative image acquisition protocol to optimize their ability to interpret the scans.”

[**Section 7.4.1.2**](#Sec7p4p1p2)

The second and third bullets have been removed, as MRI of the chest is not collected per protocol.

[**Section 8.2**](#Sec8p2)

The following language has been added: “**NOTE**: PRO-CTCAE data should not be used for determining attribution, dose modifications, or reporting serious adverse events.”

[**Section 9.0**](#Sec9p0)

Information about PRO-CTCAE has been added as the last 2 sentences of the first paragraph and a new second paragraph that begins: “**NOTE**: PRO-CTCAE…”

[**Section 9.1**](#Sec9p1)

- The following phrase has been inserted at the end of the third paragraph: “…by CTCAE, PRO-CTCAE, or both.”
- A new second paragraph that includes information about PRO-CTCAE administration has been added.
- An additional column “PRO-CTCAE v1.0 Term” has been added to the table so that the table now includes both solicited CTCAE and PRO-CTCAE adverse events. Eleven additional rows have been added to account for all PRO-CTCAE data that will be collected.

[**Section 9.3**](#Sec9p3)

The following language has been added: “**NOTE**: PRO-CTCAE data should not be used for determining attribution, dose modifications, or reporting serious adverse events.”

[**Section 9.4**](#Sec9p4)

The following language has been added: “**NOTE**: PRO-CTCAE data should not be used for determining attribution, dose modifications, or reporting serious adverse events.”

[**Section 9.4.1**](#Sec9p4p1)

Under “Additional Instructions or Exclusion to CTEP-AERS Expedited Reporting Requirements,” the third from last bullet has been removed that excluded deaths clearly due to progressive disease from CTEP-AERS reporting requirements. This is not correct, all deaths, even those clearly due to progressive disease are required to be submitted as an adverse event via CTEP-AERS.

[**Section 9.5**](#Sec9p5)

This section has been added to describe CDUS Reporting.

[**Section 13.1.2.4**](#Sec13p1p2p4)

- In the third bullet point, the hyperlinked section has been corrected from “[Section 7.4](#Sec7p4)” to “[Section 9.1](#Sec9p1).”
- The following language has been added to the end of [Section 13.1.2.4](#Sec13p1p2p4):
  - “Similarly, scores (0-4) and maximum score for each PRO-CTCAE item will be recorded for each patient separately for the 3 periods.

[**Section 13.2**](#Sec13p2)

In the first paragraph, the following has been added:

- A new fourth sentence, which begins: “We anticipate to pre-register…”
- The word “Since” to the beginning of the fifth sentence.
- At the end of the fifth sentence, the phrase: “the actual accrual will be 4 patients per month.”
- A new final sentence, that begins: “The accrual period of this study…”

[**Section 13.4.1.1**](#Sec13p4p1p1)

The phrase, “approximately 8 months after trial activation,” has been added to the end of the second sentence.

[**Section 13.4.1.2**](#Sec13p4p1p2)

Two sentences have been added to the end of the paragraph, which begin: “The final analysis will be carried out…”, and “This will occur approximately…”

[**Section 13.4.2.4**](#Sec13p4p2p4)

- The phrase, “For CTCAE data,” has been added to the beginning of the first sentence in the first paragraph.
- A new second paragraph has been added that states PRO-CTCAE will be, at the minimum, analyzed in the same manner as CTCAE data.

[**Section 13.5**](#Sec13p5)

A new section, “Safety Monitoring,” has been added. Subsequent sections have been renumbered.

[**Section 13.7**](#Sec13p7)

The Accrual Target table has been updated to reflect the **total** number of American Indian/ Alaska Natives (4). Previously the total stated “0” but this was inaccurate.

[**Section 14.0**](#Sec14p0)

The first sentence has been updated to reflect the correct number of (two) substudies available for enrolled patients. Previously the sentence stated “four” but this was inaccurate.

[**Section 14.1**](#Sec14p1)

The entire section has been removed. Subsequent sections have been renumbered.

[**Section 14.1.3**](#Sec14p1p3)

The phrase “as described in [Section 14.1.3](#Sec14p1p3)” has been removed from the final paragraph of current [Section 14.1.3](#Sec14p1p3) as this phrase referred to the previous [14.1.3](#Sec14p1p3) which has been removed.

[**Section 14.2**](#Sec14p2)

The previous [Section 14.2](#Sec14p2) and all subsections have been removed (previously, “Evaluations of Biomarkers of Response to Therapy in Alliance A021501: A021501-ST1”). This correlative science substudy has been removed from the protocol. The subsequent sections have been renumbered.

[**Appendix I**](#AppI)

The LASA and PRO-CTCAE measures have been added to [Appendix I](#AppI).

**_____________________________________________________________________________________**

**UPDATES TO THE MODEL CONSENT:**

**WHAT ARE THE STUDY GROUPS?**

Language that explains the optional participation for patients to self-report their side effects from treatment has been added to the ‘WHAT ARE THE STUDY GROUPS?’ section of the informed consent document to form new 8-11^th^ paragraphs, beginning with, “Additionally, you will be asked…”

**Additional Studies Section**

Under the Sample Collections for Laboratory Studies, language regarding the A021501-ST1 correlative substudy has been removed. The following changes have been made:

- In the third paragraph under “Optional Sample Collection…,” reference to tissue collection has been removed. Language regarding the purpose of the additional research has been changed from “how to prevent, find and treat cancer and other diseases” to “whether substances in your blood (sometimes called biomarkers) are related to the way that your body responds or doesn’t respond to the chemotherapy you receive in this trial).”
- Under “What is involved,” the following changes have been made:
  - In bullet 1, the amount of blood collected prior to treatment has changed from 2 tablespoons to 2 teaspoons, as the blood collection for ST1 has been removed. In addition, the description of the subsequent blood collections has been removed with the removal of the ST1 study.
  - The previous bullet 3 has been removed (previously starting with, “A new biopsy will also be performed…”).
- Under “What are the possible risks,” the previous bullet 2 has been removed (previously starting with, “The common risks of the additional biopsy…”) as the ST1 study has been removed.
- Under “Are there any costs or payments,” the previous second sentence has been removed (previously, “The study sponsor will pay for the cost…”).
- Under “Samples for the laboratory studies,” the second consent question has been removed (previously, “I agree to have the additional research biopsy tissues collected and I agree that my specimen sample(s) and related information may be used for laboratory studies described above.”

All references to the optional quality of life study have been removed, as well as any reference to LASA items other than baseline.

**A replacement protocol and consent document have been issued**

**____________________________________________________________**

**Attach to the front of every copy of this protocol**

**____________________________________________________________**

ALLIANCE FOR CLINICAL TRIALS IN ONCOLOGY

ALLIANCE A021501

**Preoperative extended chemotherapy vs. chemotherapy plus hypofractionated radiation therapy for borderline resectable adenocarcinoma of the head of the pancreas**

*NCI-supplied agent(s): NONE*

*Industry-supplied agent(s): NONE*

***Commercial agent(s):*** *5-fluorouracil (NSC #19893); Leucovorin (NSC #3590); Oxaliplatin (NSC #266046); Irinotecan (NSC #616348);*

*An IND exempt study*

***Study-specific credentialing requirements:*** *Institutional Imaging Certification by IROC Ohio;*

*Radiation Therapy Credentialing by IROC Houston/Rhode Island; Not a CIRB-reviewed study.*

ClinicalTrials.gov Identifier: NCT02839343

| Study Chair |  |  |
| --- | --- | --- |
| Matthew HG Katz, MD, FACS | Community Oncology Co-Chair | Medical Oncology Co-Chair |
| The University of Texas MD Anderson | James L. Leenstra, MD | Brian Wolpin, MD |
| Unit 1484, PO Box 301402 | Saint Vincent Hospital | Dana-Farber Cancer Institute |
| Houston, Texas 77230-1402 | Tel: 507-645-2655 | Tel: 617-632-6942 |
| Tel: 713-794-4660 Fax: 713-745-1921 | *leenstra.james@mayo.edu* | *bwolpin@partners.org* |
| *mhgkatz@mdanderson.org* |  |  |

| Correlative Science Co-Chair | Pathology Co-Chair | QOL Co-Chair |
| --- | --- | --- |
| Eric Collisson, MD | Wendy Frankel, MD | JoLeen M. Hubbard, MD |
| UCSF | The Ohio State University | Mayo |
| Tel: 415-476-0624 | Tel: 614-688-8660 | Tel: 507-284-8318 |
| *eric.collisson@ucsf.edu* | *wendy.frankel@osumc.edu* | *hubbard.joleen@mayo.edu* |

| Imaging Co-Chairs | | | Radiation Oncology Co-chairs | |
| --- | --- | --- | --- | --- |
| Larry Schwartz, MD | Eugene J. Koay, MD, PhD | | Joseph M. Herman, MD, MSc | Michael Chuong, MD |
| Columbia | MD Anderson | | MD Anderson | Miami Cancer Institute |
| Tel: 212-305-8994 | | Tel: 713-563-2381 | Tel: 713-745-4193 | Tel: 727-421-3443 |
| *lhs2120@cumc.columbia.edu* | *ekoay@mdanderson.org* | | *jmherman@mdanderson.org* | *michaelchu@baptisthealth.net* |

| Pancreatic Cadre Leader | ECOG-ACRIN Co-Chair | SWOG Co-Chair | Correlative Committee Chair |
| --- | --- | --- | --- |
| Andrew H. Ko MD | Robert Marsh, MD | Syed Ahmad, MD | Federico Innocenti, MD, PhD |
| UCSF | NorthShore Evanston | University of Cincinnati | UNC at Chapel Hill |
| Tel: 415-353-7286 | Tel: 847-570-2515 | Tel: 513-584-8900 | Tel: 919-966-9422 |
| *andrewko@medicine.ucsf.edu* | *rmarsh@northshore.org* | *ahmadsy@ucmail.uc.edu* | *innocent@unc.edu* |

| Primary Statistician | Secondary Statistician | Correlative Science Statistician | QOL Statistician |
| --- | --- | --- | --- |
| Fang-Shu Ou, PhD | Qian Shi, PhD | Ann L. Oberg, PhD | Amylou Dueck, PhD |
| Mayo | Mayo | Mayo | Mayo |
| Tel: 507-266-9987 | Tel: 507-538-4340 | Tel: 507-538-1556 | Tel: 480-301-6159 |
| *ou.fang-shu@mayo.edu* | *shi.qian2@mayo.edu* | *oberg.ann@mayo.edu* | *dueck.amylou@mayo.edu* |

| Alliance GI Committee Chairs | | Protocol Coordinator | Data Manager |
| --- | --- | --- | --- |
| Jeffrey Meyerhardt, MD, MPH | Eileen M. O’Reilly, MD | Alexandra LeVasseur | Pamela Fain Pribyl |
| Dana-Farber Cancer Institute Tel: 617-632-6855  *jeffrey_meyerhardt@dfci.harvard.edu* | MSKCC  Tel: 646-888-4182  *oreillye@mskcc.org* | Tel: 773-834-4518  Fax: 312-345-0117  *alevasseur@uchicago.edu* | Tel: 507-284-9549  Fax: 507-284-1902  *fainpribyl.pamela@mayo.edu* |

**Study Resources:**

| Expedited Adverse Event Reporting  https://eapps-ctep.nci.nih.gov/ctepaers/ | Medidata Rave® iMedidata portal  https://login.imedidata.com | |
| --- | --- | --- |
| OPEN (Oncology Patient Enrollment Network)  https://open.ctsu.org | Biospecimen Management System  http://bioms.allianceforclinicaltrialsinoncology.org | |
|  | | |
| Protocol Contacts: | | |
| A021501 Nursing Contact  Barb Kleiber  The Ohio State University  Tel: 614-293-1815  E-mail: *barbara.kleiber@osumc.edu* | | A021501 Pharmacy Contact  Zoe Ngo, PharmD  University of California San Francisco  Tel: 415-514-3878  Email: *zoe.ngo@ucsf.edu* |
| Alliance Biorepository  at Washington University (WUSTL)  Washington University at St. Louis  BJC Institute of Health  425 S. Euclid Ave, Room 1H-05-135  St. Louis, MO 63110-1005  Tel: 314-454-7615 Fax: 314-454-5525  *tbank@wudosis.wustl.edu* | | IROC Ohio (Alliance Imaging Core Lab)  The Ohio State University  Wright Center of Innovation  395 W. 12th Ave., RM #414  Columbus, OH 43240  Tel: 614-293-2929 Fax: 614-293-9275  *Alliance021501@IROCOhio.org* |
| IROC Rhode Island QA Center  640 George Washington Highway, Building B, Suite 201  Lincoln, Rhode Island 02865-4207  Phone: (401) 753-7600  Fax: (401) 753-7601  *IROCRI@QARC.org* | | |

| Protocol-related questions may be directed as follows: | |
| --- | --- |
| Questions | Contact (via email) |
| Questions regarding patient eligibility, treatment, and dose modification: | Study Chair, Nursing Contact, Protocol Coordinator, and (where applicable) Data Manager |
| Questions related to data submission, RAVE or patient follow-up: | Data Manager |
| Questions regarding the protocol document and model informed consent: | Protocol Coordinator |
| Questions related to IRB review | Alliance Regulatory Inbox regulatory@allianceNCTN.org |
| Questions regarding CTEP-AERS reporting: | Regulatory Affairs Manager  regulatory@allianceNCTN.org  (773) 702-9814 |
| Questions regarding specimens/specimen submissions: | Appropriate Alliance Biorepository |

| Document History | Effective Date: |  |  |
| --- | --- | --- | --- |
| Pre-Activation | 08/24/2016 |  |  |
| Activation  Update #01 | XX/XX/2016  11/01/2016 |  |  |

CANCER TRIALS SUPPORT UNIT (CTSU) ADDRESS AND CONTACT INFORMATION

| **For regulatory requirements:** | **For patient enrollments:** | **For study data submission:** |
| --- | --- | --- |
| Regulatory documentation can be submitted to the CTSU via:  **ONLINE:**  Regulatory Submission Portal (Sign in at [www.ctsu.org](http://www.ctsu.org), and select the Regulatory Submission sub-tab under the Regulatory tab).  **EMAIL:**  [CTSURegulatory@ctsu.coccg.org](mailto:CTSURegulatory@ctsu.coccg.org) (regulatory documentation only).  **FAX:**  215-569-0206  **MAIL:**  CTSU Regulatory Office  1818 Market Street, Suite 1100  Philadelphia, PA 19103  For regulatory questions call the CTSU Regulatory Help Desk at 1-866-651-CTSU | Please refer to the patient enrollment section of the protocol for instructions on using the Oncology Patient Enrollment Network (OPEN) which can be accessed at https://www.ctsu.org/OPEN_SYSTEM/ or https://OPEN.ctsu.org.  Contact the CTSU Help Desk with any OPEN-related questions at ctsucontact@westat.com. | Data collection for this study will be done exclusively through Medidata Rave. Please see the data submission section of the protocol for further instructions. |
| The most current version of the **study protocol and all supporting documents** must be downloaded from the protocol-specific page of the CTSU Member website located at https://www.ctsu.org. Access to the CTSU members’ website is managed through the Cancer Therapy and Evaluation Program - Identity and Access Management (CTEP-IAM) registration system and requires user log on with CTEP-IAM username and password. Permission to view and download this protocol and its supporting documents is restricted and is based on person and site roster assignment housed in the CTSU RSS. | | |
| **For clinical questions (i.e., patient eligibility or treatment-related)** see Protocol Contacts, Page 2*.* | | |
| **For non-clinical questions (i.e., unrelated to patient eligibility, treatment, or clinical data submission)** contact the CTSU Help Desk by phone or e-mail:  CTSU General Information Line – 1-888-823-5923, or ctsucontact@westat.com. All calls and correspondence will be triaged to the appropriate CTSU representative. | | |
| **The CTSU website is located at** https://www.ctsu.org. | | |

**Preoperative extended chemotherapy vs. chemotherapy plus hypofractionated radiation therapy for borderline resectable adenocarcinoma of the head of the pancreas**

| **Pre-Registration Eligibility Criteria (see** [**Section 3.2.1**](#Sec3p2p1)**)** | **Required Initial Laboratory Values** | |
| --- | --- | --- |
| Cytologic or histologic proof of adenocarcinoma of the | Absolute neutrophil count (ANC) | ≥ 1500/mm^3^ |
| pancreatic head or uncinate process. |  |  |
| TNM Stage: T1-4N0-1orNxM0 | Platelet Count | ≥ 100,000/mm^3^ |
| Local radiographic reading consistent with borderline resectable cancer of the pancreatic head | Creatinine | ≤ 1.5 x upper limit of normal (ULN) |
| **Registration Eligibility Criteria (see** [**Section 3.3**](#Sec3p3)**)** | Calc. Creatinine or Clearance (see Alliance website) | > 45 mL/min |
| Confirmation of radiographic stage as borderline resectable by central radiographic review |  |  |
| No prior chemotherapy or radiation for pancreatic cancer and no definitive resection of pancreatic cancer | Total Bilirubin | ≤ 2.0 x mg/dL |
| No strong CYP3A4 inhibitors or inducers (See [Section 3.3.3](#Sec3p3p3)) |  |  |
| No grade ≥ 2 neuropathy  No known Gilbert’s Syndrome or known homozygosity for UGAT1A1*28 polymorphism. | AST / ALT | ≤ 2.5 x ULN |
| No uncontrolled gastric ulcer disease (Grade 3 gastric ulcer disease) within 28 days of registration | | |
| Not pregnant and not nursing, because this study involves an agent that has known genotoxic, mutagenic and teratogenic effects. | | |
| Age ≥ 18 years  ECOG Performance Status 0 or 1 | | |
|  | | |

**Schema**

| Patient with BLR PDAC (Inter-group Definition) | 🡪 | PRE-REGISTRATION | 🡪 | Central Review by IROC Ohio | 🡪 | REGISTRATION/RANDOMIZATION | 🡪 | Arm 1 | mFOLF-  IRINOX  x  4 cycles | RESTAGE | mFOLFIRINOX  x  4 cycles | | RESTAGE (Central Review by IROC Ohio) | Surgery | | RESTAGE | FOL-FOX  x  4 cycles | FOLLOW-UP |
| --- | --- | --- | --- | --- | --- | --- | --- | --- | --- | --- | --- | --- | --- | --- | --- | --- | --- | --- |
|  |  |  |  |  |  |  |  |  |  | | | | | | | | | |
|  |  |  |  |  |  |  | 🡪 | Arm 2 | mFOLF-  IRINOX  x  4 cycles | RESTAGE | mFOLF-IRINOX  x  3 cycles * | RT | RESTAGE (Central Review by IROC Ohio) | | Surgery | RESTAGE | FOL-FOX  x  4 cycles | FOLLOW-UP |

* RT simulation and EUS/fiducial marker placement is performed during cycle 5 or 6 of mFOLFIRINOX

Surgery and imaging must be conducted at the registering institution. Radiation therapy must be performed at a facility participating in the IROC monitoring program. Chemotherapy may be given at a non-registering institution. If the NCTN Group credited for enrollment is a non-Alliance Group, then other requirements from the credited Group may apply. All protocol conduct must be followed and the registering institution is responsible for ensuring all data is reported per protocol.

Treatment is to continue until disease progression or unacceptable adverse event. Patients will be followed for 5 years or until death, whichever comes first.

**Please refer to the full protocol text for a complete description of the eligibility criteria (**[**Section 3.0**](#Sec3p0)**) and treatment plan (**[**Section 7.0**](#Sec7p0)**).**

**All sites must fulfill imaging and radiotherapy credentialing requirements. Refer to** [**Section 15.0**](#Sec15p0) **of the protocol for full description of requirements.**

**Table of Contents**

[1.0 Background 7](#_Toc463874463)

[1.1 Background and Rationale 7](#_Toc463874464)

[1.2 Rationale for Trial Design 8](#_Toc463874465)

[1.3 Trial Importance 8](#_Toc463874466)

[1.4 Pertinent Existing Data 9](#_Toc463874467)

[1.5 Impact of the Trial 13](#_Toc463874468)

[2.0 Objectives 14](#_Toc463874469)

[2.1 Primary Objective 14](#_Toc463874470)

[2.2 Secondary Objectives 14](#_Toc463874471)

[2.3 Other Objective 14](#_Toc463874472)

[2.4 Correlative Science Objectives 14](#_Toc463874473)

[3.0 Patient Selection 15](#_Toc463874474)

[3.1 On-Study Guidelines 15](#_Toc463874475)

[3.2 Pre-Registration Eligibility Criteria 15](#_Toc463874476)

[3.3 Eligibility Criteria 16](#_Toc463874477)

[4.0 Patient Registration 18](#_Toc463874478)

[4.1 CTEP Investigator Registration Procedures 18](#_Toc463874479)

[4.2 CTEP Associate Registration Procedures / CTEP-IAM Account 18](#_Toc463874480)

[4.3 CTSU Site Registration Procedures 19](#_Toc463874481)

[4.4 Patient Pre-Registration Requirements 21](#_Toc463874482)

[4.5 Patient Registration/Randomization Requirements 21](#_Toc463874483)

[4.6 Patient Pre-Registration and Registration/Randomization Procedures 21](#_Toc463874484)

[4.7 Registration to Correlative and Companion Studies 22](#_Toc463874485)

[4.8 Stratification Factors and Treatment Assignments 22](#_Toc463874486)

[5.0 Study Calendar 23](#_Toc463874487)

[6.0 Data and Specimen Submission 25](#_Toc463874488)

[6.1 Data Collection and Submission 25](#_Toc463874489)

[6.2 Specimen Collection and Submission 25](#_Toc463874490)

[6.3 Submission of Digital Radiation Therapy Data 29](#_Toc463874491)

[6.4 Imaging Requirements, Credentialing and Submission Instructions 30](#_Toc463874492)

[7.0 Treatment Plan/Intervention 33](#_Toc463874493)

[7.1 Chemotherapy (Arm 1 and Arm 2) 34](#_Toc463874494)

[7.2 Radiotherapy – Arm 2 Only 35](#_Toc463874495)

[7.3 Surgery (Arm 1 and Arm 2) 44](#_Toc463874496)

[7.4 Imaging 48](#_Toc463874497)

[8.0 Dose and Treatment Modifications 51](#_Toc463874498)

[8.1 Ancillary Therapy, Concomitant Medications, and Supportive Care 51](#_Toc463874499)

[8.2 Dose Modifications 54](#_Toc463874500)

[9.0 Adverse Events 57](#_Toc463874501)

[9.1 Routine Adverse Event Reporting 57](#_Toc463874502)

[9.2 Surgical Adverse Events 58](#_Toc463874503)

[9.3 CTCAE Routine Reporting Requirements 58](#_Toc463874504)

[9.4 Expedited Adverse Event Reporting (CTEP-AERS) 59](#_Toc463874505)

[9.5 CDUS Reporting 62](#_Toc463874506)

[10.0 Drug Information 63](#_Toc463874507)

[10.1 General Considerations 63](#_Toc463874508)

[10.2 Oxaliplatin (Eloxatin) – NSC #266046 63](#_Toc463874509)

[10.3 Irinotecan (Camptosar) – NSC #616348 67](#_Toc463874510)

[10.4 Leucovorin Calcium – NSC #3590 69](#_Toc463874511)

[10.5 5-Fluorouracil (5-FU) (Efudex, Arucil, Carac, Fluroplex) – NSC #19893 71](#_Toc463874512)

[11.0 Measurement of Effect 73](#_Toc463874513)

[11.1 Schedule of Evaluations: 73](#_Toc463874514)

[11.2 Definitions of Measurable and Non-Measurable Disease 73](#_Toc463874515)

[11.3 Guidelines for Evaluation of Measurable Disease 74](#_Toc463874516)

[11.4 Measurement of Treatment/Intervention Effect 74](#_Toc463874517)

[11.5 Definitions of Analysis Variables 78](#_Toc463874518)

[11.6 Surgical Pathologic Determination of Treatment Response 78](#_Toc463874519)

[11.7 Histopathologic Determination of R-status 78](#_Toc463874520)

[11.8 Treatment Evaluation after Completion of Surgery and Post-operative Chemotherapy 79](#_Toc463874521)

[12.0 End of Treatment/Intervention 79](#_Toc463874522)

[12.1 Duration of Treatment 79](#_Toc463874523)

[12.2 Managing Ineligible Patients and Registered Patients Who Never Receive Protocol Intervention 80](#_Toc463874524)

[12.3 Extraordinary Medical Circumstances 81](#_Toc463874525)

[13.0 Statistical Considerations 81](#_Toc463874526)

[13.1 Study Endpoints 81](#_Toc463874527)

[13.2 Sample Size 82](#_Toc463874528)

[13.3 Power Justification 82](#_Toc463874529)

[13.4 Statistical Analysis Plan 84](#_Toc463874530)

[13.5 Safety Monitoring 86](#_Toc463874531)

[13.6 Descriptive Factors 86](#_Toc463874532)

[13.7 Inclusion of Women and Minorities 87](#_Toc463874533)

[14.0 Correlative and Companion Studies 88](#_Toc463874534)

[14.1 Pharmacogenetic Studies in Alliance A021501: A021501-PP1 88](#_Toc463874535)

[14.2 Imaging Science Studies in Alliance A021501: Alliance A021501-IM1 90](#_Toc463874536)

[15.0 General Regulatory Considerations and Credentialing 94](#_Toc463874537)

[15.1 Institutional Credentialing 94](#_Toc463874538)

[16.0 References 96](#_Toc463874539)

[Appendix I: Registration QOL/Mental Well-being/Fatigue and PRO-CTCAE Measures 99](#_Toc463874540)

[Appendix II: Definition Of Borderline Resectable Disease Table For Section 3.2.1 (Eligibility) 108](#_Toc463874541)

[Appendix III: Patient Drug Information Handout and Wallet Card 109](#_Toc463874542)

[Appendix IV: Standard lymphadenectomy Illustration 112](#_Toc463874543)

[Appendix V: Fiducial Marker Ordering Information 113](#_Toc463874544)

# 1.0 Background

## 1.1 Background and Rationale

Borderline resectable pancreatic ductal adenocarcinoma (PDAC) is an increasingly recognized clinical stage of localized PDAC [1]. Although borderline resectable cancers, like locally advanced pancreatic cancers (LAPC), infiltrate to involve adjacent vital structures, the extent to which they do so is relatively minimal so surgical resection represents a technical option. However, the extent to which borderline resectable cancers infiltrate into the local anatomy is greater than that of potentially resectable cancers so the potential for incomplete (R1 or R2) operations is significant when surgery is used as primary therapy [2, 3]. Given that complete microscopic (R0) resection represents a requisite component of curative therapy for patients with PDAC, neoadjuvant regimens designed to both select patients for surgery and optimize surgical outcomes are increasingly being administered to patients with borderline resectable cancers in an attempt to maximize the likelihood for long-term survival.

The therapeutic strategy utilized most frequently for borderline resectable PDAC in the United States is currently based entirely upon consensus [4, 5]. Typically, patients first receive a predefined period of systemic chemotherapy (2 to 4 months) followed by a conventional 5-6 week course of gemcitabine or 5-FU-based chemoradiation. Patients who complete this multimodality regimen without evidence of disease progression undergo pancreatectomy with curative intent; those who progress prior to the intended operation are treated with additional non-operative therapies with palliative intent. This approach leverages theoretical benefits associated with systemic chemotherapy (e.g., systemic antitumor activity upon micrometastatic disease known to exist in almost all patients), chemoradiation (e.g., “sterilization” of surgical margins) and time (e.g., patient selection) in an attempt to maximize systemic control, local control, and ultimately, overall survival.

Three important considerations are noteworthy in this regard. First, the sequential administration of chemotherapy and chemoradiation was proposed at a time when gemcitabine—a systemic agent with a response rate against PDAC less than 10%—was standard. In the “gemcitabine era”, the rationale for following systemic chemotherapy with chemoradiation was the latter’s direct cytotoxic effect on the primary tumor, possibly leading to an improvement in local control or even a reduction in the size or anatomic extent (“downstaging”) of the cancer to an extent that might facilitate an R0 resection. However, the added benefit of conventional chemoradiation remains unclear in this setting, as it is unclear in other stages of localized PDAC [6-9]. Indeed, the sequential administration of gemcitabine-based chemotherapy and chemoradiation led to a radiographic response in only 12% of borderline resectable primary tumors evaluated, and downstaged only 0.8% to technically resectable in one large, albeit retrospective analysis of this approach [10].

Second, chemoradiation as routinely administered to patients with localized PDAC and as advocated in the consensus guidelines is dosed over a duration of 5-6 weeks prior to a 6 week break before surgery. Because chemoradiation acts only upon the primary tumor and regional lymph nodes, the systemic micrometastatic disease that may exist in all patients with localized PDAC is suboptimally untreated for as many as 3-4 months prior to resection.

The third consideration is that the daily radiotherapy treatments used in conventional chemoradiation regimens are associated with significant direct and opportunity costs as patients must spend time away from work or even family, and they may be associated with moderate toxicity. And, to the extent that clinical trials of novel treatment strategies may be conducted at high volume centers located at a distance from patients’ homes, the length of time associated with conventional chemoradiation represents a critical barrier both to enthusiastic enrollment to such trials and to progression in the field.

The consensus treatment algorithm of systemic chemotherapy and conventional chemoradiation that is routinely used for patients with borderline resectable PDAC must be rigorously evaluated in the context of significant advances in both chemotherapy and radiation that have occurred within the past 5 years. The systemic regimens now in routine use for patients with metastatic pancreatic cancer—FOLFIRINOX and gemcitabine plus nab-paclitaxel—are associated with objective response rates of 32% and 23%, respectively, in the setting of metastatic disease [11, 12]. Given the high response rates associated with FOLFIRINOX and gemcitabine plus nab-paclitaxel, it is conceivable that continuous dosing of chemotherapy alone may allow better treatment of the systemic disease that more commonly represents the source of morbidity and mortality among patients with PDAC without forgoing any theoretical effect on the primary tumor previously associated with chemoradiation. Furthermore, to the extent that radiation therapy may have a role in “sterilizing” the surgical margins of patients with borderline resectable disease, thereby enhancing rates of R0 resection and local control, attractive alternatives to conventional chemoradiation now exist that appear to yield rates of local control and acute and late toxicity rates equivalent to those associated with conventional chemoradiation using dosing schedules as short as 5 days [13, 14].

In this trial, we seek to identify one treatment program that meet clinically-relevant benchmarks for activity, and which can provide the foundation for future randomized comparison trials in a space where there are currently limited high-level data to guide treatment choices.

## 1.2 Rationale for Trial Design

Herein we propose to evaluate the efficacy of two rational neoadjuvant treatment backbones in a randomized study. The study builds upon our previous work as requested by CTEP and will define standard preoperative treatment regimens to which future novel regimens can and will be compared in subsequent randomized trials.

We have proposed a randomized frequentist design using exact binomial distribution for this study. This design focuses on evaluating whether clinical meaningful improvement of survival rate can be achieved by the tested regimens comparing to historical data. The evaluation of each treatment arm will be conducted separately, based on a practical assessment of whether the arm is “clinically relevant” alone, as well as the outcome comparisons between two treatments if both arms are deemed promising at the end of the trial. This study uses a critically important interim endpoint (R0 resection rate) that is different from the final endpoint (18 month OS rate) and an interim analysis which allows elimination of any futile arm after only 30 patients enrolled to that arm. If both arms are deemed promising at the end of the trial, a pick-a-winner strategy will be employed to choose one arm as the “winner”. Using a fast maturing end point, R0 resection rate, for interim analysis prevent the possibility of halting the trial at interim analysis while the whole study still powered to detect clinical meaningful improvement in OS rate.

## 1.3 Trial Importance

A trial to define a standard preoperative treatment backbone for borderline resectable PDAC is critically necessary to provide a regimen for current use and a fundamental therapeutic backbone to which future novel regimens can be compared in subsequent studies.

The concept and design of this trial has evolved within the Pancreatic Cancer Task Force over the past two years and has been vetted with multiple votes and polls of its members. The trial also represents a natural next step in an investigational program that has proceeded with the advice, investment, and involvement of CTEP. Indeed, at the request of CTEP, we performed an initial pilot study designed to rapidly assess the feasibility of a multi-institutional study of borderline resectable PDAC and to develop a standardized clinical and research infrastructure (e.g., rapid review of imaging, pathologic assessments, quality control of radiation protocols) specific to this disease stage that is necessary to study it [15, 16]. That trial represented one of the most collaborative studies that has ever been conducted in the pancreas cancer space: it met its accrual endpoint early and within a year due to the enthusiasm of centers from the Alliance, ECOG, SWOG and RTOG. Alliance Trial A021101—the results of which were recently presented as an oral presentation at the 2015 annual meeting of ASCO and published on the JAMA surgery website [17]—demonstrated that rapid, real-time central review of imaging is feasible, established other infrastructural elements viewed as critical to the conduct of multimodality treatment trials for pancreatic cancer, and set the stage for the subsequent randomized phase II study that CTEP agreed would represent the natural next step in this line of investigation.

This successor trial has the support of all major clinical trials groups as well as the Pancreatic Cancer Task Force. Furthermore, the Task Force has declared the search for a treatment paradigm for patients with this stage of disease as a strategic priority.

If both arms of this trial are found to be able to provide clinically meaningful improvement of OS rate, “pick-a-winner” approach will be conducted and one regimen will be recommend as the rational backbone for future regimens and studies. If only one arm is “clinically relevant”, then that arm will serve as the control arm for future randomized trials. If no arm is “clinically relevant” then the rationale for an alternative approach should be investigated.

## 1.4 Pertinent Existing Data

Existing data are primarily from small, single-center, and often retrospective studies exploring novel treatment strategies for borderline resectable PDAC. The results of these studies are therefore difficult to compare because of heterogeneity in trial design, variability in enrolled populations, and absence of quality control. However, the following observations are useful:

1.4.1 Neoadjuvant chemotherapy alone for resectable PDAC

Neoadjuvant chemotherapy alone does not preclude R0 resection of radiographically resectable PDAC, and it is associated with reasonable rates of OS. In a small European study of 28 patients with resectable pancreatic cancer, an R0 resection was accomplished in 80% of patients who received gemcitabine and cisplatin prior to intended resection [18]. In a more recent single-center study, 27 (71%) of 38 resectable patients who received preoperative gemcitabine and oxaliplatin underwent surgical resection, and 26 of these received postoperative gemcitabine. The median OS for all 38 patients was 27.2 months [19].

1.4.2 Neoadjuvant chemotherapy alone for borderline resectable PDAC

Neoadjuvant chemotherapy is well tolerated and selects patients with borderline resectable PDAC for surgical resection. In a retrospective evaluation of 64 patients treated with neoadjuvant gemcitabine/docetaxel and a variety of second line agents, 31 (48%) were resected; 87% had an R0 resection and 10% (N=3) had a complete pathologic response. Median OS of all 64 patients was 23.6 months; that of unresected patients was 15.4 months [20].

1.4.3 Neoadjuvant gemcitabine-based chemotherapy with conventional chemoradiation for borderline resectable PDAC

Neoadjuvant gemcitabine-based chemotherapy with standard chemoradiation is associated with favorable outcomes and high R0 resection rates in patients with borderline resectable PDAC but is not associated with a high radiographic response based on RECIST or “downstaging” of the pancreas tumor. In a retrospective evaluation of 129 patients with borderline resectable PDAC, only 12% had a RECIST response to neoadjuvant therapy that consisted of gemcitabine-based systemic therapy followed by conventional chemoradiation with concurrent gemcitabine or capecitabine. Only 1 patient (0.8%) had their disease downstaged to resectable status following receipt of chemoradiation. Nonetheless, 66% of all patients underwent pancreatectomy and their median OS was 22 months (95% CI: 14-30 months)[10].

1.4.4 Neoadjuvant therapy with FOLFIRINOX and conventional chemoradiation for borderline resectable PDAC

In our initial pilot study we showed that FOLFIRINOX-based multimodality therapy for borderline resectable PDAC is well tolerated and study of this approach is feasible in the cooperative group setting [16]. 22 of 23 enrolled patients started therapy (median age 64 years, 64% ECOG PS 0). All patients completed mFOLFIRINOX and 21 (95%) completed CRT. Fourteen (64% [95% confidence interval (CI), 41-83%]) patients had grade ≥3 adverse events during preoperative therapy. The best RECIST radiographic responses during preoperative treatment were 2 CR, 4 PR, 14 SD and 2 PD. 7 patients did not undergo planned resection due either to progression (6) or refusal (1). 15 (68% [95% CI, 49–88%]) patients underwent pancreatectomy. Fourteen (93%) operations had microscopically negative margins, 5 (33%) operative specimens had <5% residual viable tumor cells, and 2 (13%) specimens had pathologic complete responses. Overall survival of all patients at 18 months was 50%.

1.4.5 Standard chemoradiation for locally advanced PDAC

The role of CRT in patients with locally advanced disease is unclear. The Gastrointestinal Tumor Study Group 9283 [GISTG 1998] and Eastern Cooperative Oncology Group 4201 [21] studies reported improved OS with chemoradiation. However, the chemoradiation arms were associated with substantial grade 3-4 toxicity. In a retrospective review of two prospective studies, the Groupe Coopérateur Multidisciplinaire en Oncologie (GERCOR) reported a superior OS in patients receiving chemotherapy and CRT versus those receiving chemotherapy alone [22]. The Fédération Francophone de Cancérologie Digestive and Société Francophone de Radiothérapie Oncologique (FFCD-SFRO) study reported inferior OS and worse toxicity with the addition of chemoradiation to chemotherapy [23]. Recently, preliminary results of the phase III GERCOR LAP 07 study demonstrated no OS benefit with the addition of chemoradiation [9].

1.4.6 Hypofractionated radiation therapy: modern techniques

Hypofractionated radiation therapy may be delivered using two similar techniques over an abbreviated 5 day schedule. Both short-course hypofractionated image guided radiation therapy (HIGRT, 5 Gy x 5) and stereotactic body radiation therapy (SBRT, 6.6 Gy x 5) are delivered over 5 consecutive days (see figures below for comparison). HIGRT delivers a homogeneous dose of radiation therapy to the tumor plus a 0.5-1 cm expansion to account for microscopic extension and set-up error as well as breathing motion. This means that the treated volume (tumor plus margin) receives a homogeneous dose of 5 Gy per day—a dose limited to minimize the risk of long-term bowel or stomach toxicity in the event the patient is not able to undergo subsequent surgery. SBRT also delivers RT over 5 days but a heterogeneous dose is delivered and the volume includes only the tumor plus 2-3 mm. Using this technique a higher daily dose of RT is given (6.6 Gy). However since the dose is heterogeneous, some areas of the target will receive up to 8 Gy per day while other areas of the target will receive <6.6 Gy. With SBRT the tumor is treated to a higher total dose and consequently adjacent nodal regions are not as well covered as compared to HIGRT to minimize dose to critical normal structures including the duodenum, stomach, and small bowel.


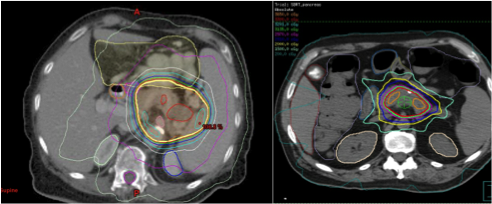


Figure S1 A. HIGRT B. SBRT

1.4.7 Rationale for hypofractionated (HIGRT/SBRT) dose and fractionation (5-6.6 Gy x 5)

It is unclear whether HIGRT or SBRT is better in the neoadjuvant setting and both have been shown to be effective in sterilizing the tumor margin of primary pancreatic tumors. HIGRT is less technically demanding than SBRT and therefore can be delivered at most radiation clinics. Conversely, SBRT requires the use of motion management strategies including gating (tracking of tumor during breathing), breath hold (radiation beam is only on while the patient holds their breath at a specific position), and abdominal compression (pressure is applied to the upper abdomen to limit the range of diaphragmatic excursion and thus tumor motion) with complex radiation planning and therefore may not be able to be delivered at all radiation facilities. **Sites that enroll patients will decide whether to utilize either SBRT or HIGRT on this trial. SBRT is the recommended and preferred radiation technique for this protocol. However, if centers are not able to deliver SBRT or the patient is inappropriate for SBRT, then HIGRT will be delivered.**

At this time, there is no clear consensus regarding an optimal fractionation schedule for patients with borderline resectable or locally advanced pancreas cancer [24, 25]. To date, Stanford has treated more than 150 patients with SBRT, and this treatment has resulted in local control rates of >90% with acceptable acute GI toxicity. A single fraction of Linac-based SBRT (25 Gy x 1) has resulted in excellent tumor control. However, close to 50% of these patients developed late grade 2-5 duodenal toxicity within one year, primarily because of the proximity of the duodenum to the pancreas. Delivering hypofractionated radiation (5 or 6.6 Gy over 5 days) instead of single fraction treatment appears to result in similar tumor control with less late toxicity (≤20%) [13, 26-28].

Using the linear-quadratic formulation, the biologically equivalent dose (BED) of the proposed fractionation schedules are given in comparison to other commonly used schemes (Table S1). The proposed 6.6 Gy x 5 schedule (BED early/late 54.8/105.6) closely approximates that of standard chemoradiation (BED early/late 60/83.3), but without concurrent chemotherapy and treating a smaller tumor margin (0.3 cm vs. ~2 cm). Furthermore, the proposed 6.6 Gy x 5 fractionation schedule has a much lower late BED (105.6 vs. 233.3) with a similar early BED (54.8 vs. 87.5) as the previous 25 Gy x 1 regimen. In this study, we will refine our current understanding of radiation tolerance of the pancreas and adjacent organs.

Table S1. Estimated biological equivalent dose (BED) of Fractionation Schedules

| **Dose / fractions** | **Nodes Tx** | **Chemo** | **BED**  **early** | **BED**  **late** |
| --- | --- | --- | --- | --- |
|  |  |  | α/β=10 | α/β=-3 |
| 50 Gy/25 (st | Yes | Yes | 60 | 83.3 |
| 30 Gy/10 | Yes | Yes | 39 | 60 |
| **25 Gy/5** | **Yes** | **No** | **37.5** | **66.7** |
| **33 Gy/5** | **No** | **No** | **54.8** | **105.6** |
| 25 Gy/1 | No | No | 87.5 | 233.3 |

1.4.8 Neoadjuvant hypofractionated radiation therapy for resectable PDAC

Hypofractionated radiation therapy, either alone or preceded by systemic therapy, does not preclude R0 resection of radiographically resectable PDAC, and it is associated with reasonable rates of local control and OS. Two consecutive single-arm trials performed at MD Anderson evaluated gemcitabine-based CRT (30 Gy in 10 treatments) with and without induction gemcitabine and cisplatin [29]. In these two trials, the most common reason patients failed to undergo resection following chemoradiation was metastatic disease; only 1 out of 176 patients had isolated local progression that precluded surgery. The overall median survival durations of the no induction versus induction chemotherapy arms were 22.7 months and 17.4 months, respectively, and the median survival durations of the resected patients was 34 and 31 months. These results were encouraging and better than historical controls of patients with resectable cancers who received an initial operation.

In a study of preoperative proton-based radiation (5 Gy x 5) with capecitabine, followed by surgery and adjuvant gemcitabine, toxicity was low (4.1% grade 3 and no grade 4/5). 37/48 patients underwent surgery and progression-free survival of all 48 patients was 10 months and median overall survival was 10 months. Distant recurrence was the primary mode of failure (72.9%), highlighting the need for more aggressive systemic therapy in this population [30].

1.4.9 Neoadjuvant hypofractionated radiation therapy for borderline resectable PDAC

Hypofractionated radiation therapy may be effective in the neoadjuvant setting for patients with borderline resectable disease. A retrospective study including 73 PDAC patients (57 borderline resectable, 16 locally advanced) who received induction chemotherapy followed by 5-fraction SBRT at Moffitt Cancer Center was published in 2013 [31]. Most borderline resectable patients received induction GTX (67%); only 5% received induction FOLFIRINOX. Among the borderline resectable patients, 56% underwent surgery with 97% achieving negative margins. Median OS in the patients who underwent margin-negative resection was significantly higher than in unresected patients (19.3 vs. 12.3 months; P=.03). Furthermore this approach was well tolerated with no acute grade 3+ toxicity reported and only 5.3% late grade 3+ toxicity. This single institution series was recently updated to include a total of 159 patients (110 borderline resectable, 49 locally advanced) [14]. Among the borderline resectable patients, 51% underwent surgery with 97% achieving negative margins. Median OS in the resected borderline resectable patients was 34 months. Acute grade 3+ toxicity was 2% and late grade 3+ toxicity was 5%.

Single-institution data from Johns Hopkins reported patients who received 25-33 Gy SBRT in 5 fractions followed by surgical resection. Median OS from date of diagnosis was 18.4 months. Of the 19 patients (21.6%) who underwent surgery following SBRT, 11% had borderline resectable cancers, and 84% had margin-negative resections [27].

1.4.10 Hypofractionated radiation therapy for locally advanced PDAC

Results of SBRT in patients with locally advanced PDAC are encouraging. Early phase I/II studies using single-fraction SBRT (25 Gy in 1 fraction) demonstrated excellent freedom from local progression (FFLP) at 1 year (>90%) and minimal acute toxicity in patients with locally advanced disease, but resulted in high late grade 2-4 gastrointestinal (GI) toxicity [24, 32-35]. A single-arm phase II multi-institutional study to determine whether gemcitabine with fractionated SBRT (in 5 fractions of 6.6 Gy, total 33.0 Gy) would achieve reduced late grade 2-4 GI toxicity compared with a historical cohort of patients treated with gemcitabine and a single 25 Gy-fraction of SBRT was subsequently conducted [Herman 2014]. Forty-nine LAPC patients received up to 3 doses of GEM (1,000 mg/m2) followed by a one-week break and SBRT (33.0 Gy in 5 fractions). Following SBRT, patients continued GEM until progression or toxicity. Rates of acute and late (primary endpoint) grade ≥2 gastritis, fistula, enteritis, or ulcer toxicities were 2% and 11%, respectively. QLQ-C30 global quality of life scores remained stable from baseline to after SBRT (67 at baseline, median change of 0 at both follow-ups; P>.05 for both). Patients reported a significant improvement in pancreatic pain (P<.001) 4 weeks after SBRT on the QLQ-PAN26 questionnaire. Median plasma CA19-9 was reduced following SBRT (median time post-SBRT 4.2 weeks, 220 vs. 62 U/mL, p<0.001). Median overall survival was 13.9 months (95% CI, 10.2-16.7). FFLP at 1 year was 78%. Four patients with LAPC at diagnosis (8%) underwent margin- and node-negative resections.

## 1.5 Impact of the Trial

A trial to help understand the respective roles of preoperative systemic and local therapies for patients with borderline resectable PDAC is critically necessary. This study will establish a treatment strategy to which future novel regimens can be compared in future trials. The study will compare the efficacy of two commonly employed preoperative regimens, one of which only utilizes systemic therapy and one that also uses a novel local treatment modality. The trial will provide the only multi-center, quality-controlled data for this stage of disease.

# 2.0 Objectives

2.1 Primary Objective

To evaluate and estimate 18 months overall survival (OS) rate of patients with borderline resectable PDAC receiving neoadjuvant therapy consisting of one of the following regimens prior to intended surgical resection and adjuvant therapy with 4 cycles of FOLFOX:

1) Arm 1: 8 cycles of systemic FOLFIRINOX, and/or

2) Arm 2: 7 cycles of systemic FOLFIRINOX followed by hypofractionated radiation therapy

2.2 Secondary Objectives

2.2.1 To evaluate and estimate the R0 resection rates in patients receiving each of the two multimodality treatment regimens.

2.2.2 To evaluate and estimate the event-free survival in patients receiving each of the two multimodality treatment regimens.

2.2.3 To evaluate and estimate the pathologic compete response (pCR) rates in patients receiving each of the two multimodality treatment regimens

2.2.4 To assess the adverse events (AE) profile and safety of each treatment arm, using the CTC-AE and PRO-CTCAE.

2.3 Other Objective

Results of the primary analysis will be examined for consistency, while taking into account the stratification factors and/or covariates of baseline overall QOL, mental well-being, physical well-being, and fatigue.

## 2.4 Correlative Science Objectives

2.4.1 Pharmacogenetic studies in Alliance A021501: A021501-PP1

To test the effect of the rs2853564 *VDR* variant on OS rate and discover novel candidate genes associated with OS and severe toxicity of chemotherapy by using genome-wide genotyping approaches.

2.4.2 Imaging science studies in Alliance A021501: A021501-IM1

- To evaluate risk classification previously developed by Koay et al [36] using NAUC.
- To access prognostic value of NAUC ration defined as post-neoadjuvant NAUC divided by pre-neoadjuvant therapy NAUC.
- To evaluate risk classification previously developed by Koay et al using delta measure.

# 3.0 Patient Selection

For questions regarding eligibility criteria, see the Study Resources page. Please note that the Study Chair cannot grant waivers to eligibility requirements.

## 3.1 On-Study Guidelines

This clinical trial can fulfill its objectives only if patients appropriate for this trial are enrolled. All relevant medical and other considerations should be taken into account when deciding whether this protocol is appropriate for a particular patient. Physicians should consider the risks and benefits of any therapy, and therefore only enroll patients for whom this treatment is appropriate.

Physicians should consider whether any of the following may render the patient inappropriate for this protocol:

- Psychiatric illness which would prevent the patient from giving informed consent.
- Medical condition such as uncontrolled infection (including HIV), uncontrolled diabetes mellitus or cardiac disease which, in the opinion of the treating physician, would make this protocol unreasonably hazardous for the patient.
- “Currently active” second malignancy other than non-melanoma skin cancers or cervical carcinoma in situ. Patients are not considered to have a “currently active” malignancy if they have completed therapy and are free of disease for ≥ 3 years.
- Patients who are unable to lie flat on their back on a hard surface with their arms up for at least 20 minutes.

In addition:

- Women and men of reproductive potential should agree to use an appropriate method of birth control throughout their participation in this study due to the teratogenic potential of the therapy utilized in this trial. Appropriate methods of birth control include abstinence, oral contraceptives, implantable hormonal contraceptives or double barrier method (diaphragm plus condom).

## 3.2 Pre-Registration Eligibility Criteria

Use the spaces provided to confirm a patient’s eligibility by indicating Yes or No as appropriate. It is not required to complete or submit the following page(s).

When calculating days of tests and measurements, the day a test or measurement is done is considered Day 0. Therefore, if a test were done on a Monday, the Monday one week later would be considered Day 7.

A female of childbearing potential is a sexually mature female who: 1) has not undergone a hysterectomy or bilateral oophorectomy; or 2) has not been naturally postmenopausal for at least 12 consecutive months (i.e., has had menses at any time in the preceding 12 consecutive months).

3.2.1 Documentation of disease

**___ Pathology:** Cytologic or histologic proof of adenocarcinoma of the pancreatic head or uncinate process. Diagnosis should be verified by local pathologist.

___ **TNM Stage**: TX, T1-4N0-1orNxM0*

*M1 disease includes spread to distant lymph nodes, organs, and ascites

**___ Criteria for borderline resectable disease:** Local radiographic reading must be consistent with borderline resectable cancer of the pancreatic head as defined by intergroup radiographic criteria and must meet **any one or more** of the following on CT/MRI:

- An interface is present between the primary tumor and the **superior mesenteric vein or portal vein** and measures ≥ **180°** of the circumference of the vessel wall
- Short-segment **occlusion of the SMV-PV** is present with normal vein above and below the level of obstruction that is **amenable to resection and venous reconstruction**
- Short segment **interface** (of any degree) is present between tumor and **hepatic artery** with normal artery proximal and distal to the interface that is **amenable to resection and reconstruction**
- An interface is present between the tumor and **superior mesenteric artery or celiac axis** measuring < **180°** of the circumference of the vessel wall

Patients with less extensive disease than the above four (4) criteria are considered potentially resectable and are **NOT** eligible

Patients with more extensive disease than the above 4 criteria are considered locally advanced and are **NOT** eligible.

In addition patients with the following are considered locally advanced and are **NOT** eligible:

- Any interface between the tumor and the aorta.

**See** [**Appendix II**](#AppII) **for additional clarification and definitions of less and more extensive disease.**

## 3.3 Eligibility Criteria

3.3.1 Disease Status

___ Confirmation of radiographic stage as borderline resectable disease by real-time Alliance central radiographic review

3.3.2 Prior Treatment

___ No prior chemotherapy or radiation for pancreatic cancer

___ No definitive resection of pancreatic cancer

3.3.3 Concomitant Medications

___ Chronic concomitant treatment with strong inhibitors of CYP3A4 is not allowed on this study. Patients on strong CYP3A4 inhibitors must discontinue the drug for 14 days prior to registration on the study. See [Section 8.1.10](#Sec8p1p10) for more information.

___ Chronic concomitant treatment with strong CYP3A4 inducers is not allowed. Patients must discontinue the drug 14 days prior to the start of study treatment. See [Section 8.1.11](#Sec8p1p11) for more information.

3.3.4 Medical History

___ No grade ≥ 2 neuropathy

___ No known Gilbert’s Syndrome or known homozygosity for UGAT1A1*28 polymorphism.

___ No uncontrolled gastric ulcer disease (Grade 3 gastric ulcer disease) within 28 days of registration

3.3.6 Pregnancy and Nursing Status

___ Not pregnant and not nursing, because this study involves an agent that has known genotoxic, mutagenic and teratogenic effects.

Therefore, for women of childbearing potential only, a negative pregnancy test done ≤ 7 days prior to registration is required.

3.3.7 Age ≥ 18 years

3.3.8 ECOG Performance Status 0 or 1

3.3.9 Required Initial Laboratory Values:

| Absolute Neutrophil Count (ANC) | ≥ 1,500/mm^3^ |
| --- | --- |
| Platelet Count | ≥ 100,000/mm^3^ |
| Creatinine | ≤ 1.5 x upper limit of normal (ULN) |
| or |  |
| Calc. Creatinine Clearance | > 45 mL/min |
| Total Bilirubin | ≤ 2.0 mg/dL |
| AST / ALT | ≤ 2.5 x upper limit of normal (ULN) |

# 4.0 Patient Registration

## 4.1 CTEP Investigator Registration Procedures

Food and Drug Administration (FDA) regulations and National Cancer Institute (NCI) policy require all investigators participating in any NCI-sponsored clinical trial to register and to renew their registration annually.

Registration requires the submission of:

• a completed Statement of Investigator Form (FDA Form 1572) with an original signature

• a current Curriculum Vitae (CV)

• a completed and signed Supplemental Investigator Data Form (IDF)

• a completed Financial Disclosure Form (FDF) with an original signature

Fillable PDF forms and additional information can be found on the CTEP website at <http://ctep.cancer.gov/investigatorResources/investigator_registration.htm>. For questions, please contact the CTEP Investigator Registration Help Desk by email at <pmbregpend@ctep.nci.nih.gov>.

## 4.2 CTEP Associate Registration Procedures / CTEP-IAM Account

The Cancer Therapy Evaluation Program (CTEP) Identity and Access Management (IAM) application is a web-based application intended for use by both Investigators (i.e., all physicians involved in the conduct of NCI-sponsored clinical trials) and Associates (i.e., all staff involved in the conduct of NCI-sponsored clinical trials).

Associates will use the CTEP-IAM application to register (both initial registration and annual re-registration) with CTEP and to obtain a user account.

Investigators will use the CTEP-IAM application to obtain a user account only. (See CTEP Investigator Registration Procedures above for information on registering with CTEP as an Investigator, which must be completed before a CTEP-IAM account can be requested.)

An active CTEP-IAM user account will be needed to access all CTEP and CTSU (Cancer Trials Support Unit) websites and applications, including the CTSU members’ website.

Additional information can be found on the CTEP website at <http://ctep.cancer.gov/branches/pmb/associate_registration.htm>. For questions, please contact the ***CTEP Associate Registration Help Desk*** by email at <ctepreghelp@ctep.nci.nih.gov>.

4.2.1 TRIAD Installations for Submission of Diagnostic Imaging and Digital RT Data

The Transfer of Imaging and Data (TRIAD) application can be used for submission of diagnostic imaging and digital RT data. TRIAD is the American College of Radiology’s (ACR) image exchange application. TRIAD provides sites participating in clinical trials a secure method to transmit DICOM, DICOM RT and other digital files. TRIAD anonymizes and validates the images as they are transferred.

Use of TRIAD for submission of imaging and RT data on this trial is optional, but preferred. TRIAD must be installed locally for this purpose. See [Section 6.3.1](#Sec6p3p1) for information on the use of TRIAD for submission of digital RT and [Section 6.4](#Sec6p4) for submission of imaging data.

Site staff who will submit images through TRIAD will need to be registered with the Cancer Therapy Evaluation Program (CTEP) and have a valid and active CTEP Identity and Access Management (IAM) account. Please refer to CTEP Registration Procedures of the protocol for instructions on how to request a CTEP-IAM account.

To submit images, the site TRIAD user must be on the site’s affiliate rosters and be assigned the 'TRIAD site user' role on the CTSU roster. Users should contact the site’s CTSU Administrator or Data Administrator to request assignment of the TRIAD site user role.

When a user applies for a CTEP-IAM account with the proper user role, he/she will need to have the TRIAD application installed on his/her workstation to be able to submit files. TRIAD installation documentation can be found by following this link https://triadinstall.acr.org/triadclient/. This process can be done in parallel to obtaining your CTEP-IAM account username and password.

If you have any questions regarding this information, please send an e-mail to the TRIAD Support mailbox at TRIAD-Support@acr.org.

Please see [Section 6.4](#Sec6p4) for further information of data submission using TRIAD.

## 4.3 CTSU Site Registration Procedures

This study is supported by the NCI Cancer Trials Support Unit (CTSU).

**IRB Approval**:

**Please note:** This study is not reviewed by the NCI Central Institutional Review Board (CIRB).

Each investigator or group of investigators at a clinical site must obtain IRB approval for this protocol and submit IRB approval and supporting documentation to the CTSU Regulatory Office before they can be approved to enroll patients. Assignment of site registration status in the CTSU Regulatory Support System (RSS) uses extensive data to make a determination of whether a site has fulfilled all regulatory criteria including but not limited to: an active Federal Wide Assurance (FWA) number, an active roster affiliation with the Lead Network or a participating organization, a valid IRB approval, and compliance with all protocol specific requirements.

4.3.1 Downloading Site Registration Documents

Site registration forms may be downloaded from the A021501 protocol page located on the CTSU members’ website. Permission to view and download this protocol and its supporting documents is restricted and is based on person and site roster assignment housed in the CTSU RSS.

- Go to https://www.ctsu.org and log in to the members’ area using your CTEP-IAM username and password
- Click on the Protocols tab in the upper left of your screen
- Either enter the protocol # in the search field at the top of the protocol tree, or
- Clink on the By Lead Organization folder to expand
- Click on the Alliance link to expand, then select trial protocol #A021501
- Click on LPO Documents, select the Site Registration Documents link, and download and complete the forms provided.

4.3.2 Requirements for A021501 Site Registration

- CTSU Transmittal Sheet (optional)
- IRB Approval
- Alliance Imaging Core Lab at IROC Ohio Credentialing (See [Section 15.1.1](#Sec15p1p1))
- Radiation Therapy Site Credentialing required prior to the delivery of radiation therapy on any protocol patient (See [Section 15.1.2](#Sec15p1p2))
- CTSU RT Facilities Inventory Form

**NOTE**: Per NCI policy all institutions that participate on protocols with a radiation therapy component must participate in the Image and Radiation Oncology Core (IROC) monitoring program. If this form has been previously submitted to CTSU it does not need to be resubmitted unless updates have occurred at the RT facility

4.3.3 Checking Your Site’s Registration Status

You can verify your site registration status on the member’s section of the CTSU website.

- Go to https://www.ctsu.org and log in to the members’ area using your CTEP-IAM username and password
- Click on the Regulatory tab at the top of your screen
- Click on the Site Registration tab
- Enter your 5-character CTEP Institution Code and click on Go

Note: The status given only reflects compliance with IRB documentation and institutional compliance with protocol-specific requirements as outlined by the Lead Network. It does not reflect compliance with protocol requirements for individuals participating on the protocol or the enrolling investigator’s status with the NCI or their affiliated networks.

4.3.4 Submitting Regulatory Requirements

Submit required forms and documents to the CTSU Regulatory Office, where they will be entered and tracked in the CTSU RSS.

ONLINE: [www.ctsu.org](http://www.ctsu.org) (members’ section) 🡪 Regulatory Submission Portal

EMAIL: [CTSURegulatory@ctsu.coccg.org](mailto:CTSURegulatory@ctsu.coccg.org) (for regulatory document submission only)

FAX: 215-569-0206

MAIL: CTSU Regulatory Office

1818 Market Street, Suite 1100

Philadelphia, PA 19103

4.3.5 Credentialing

- Alliance Imaging Core Lab at IROC Ohio Credentialing
- Radiation Therapy Site Credentialing

See [Section 15.0](#Sec15p0) for credentialing requirements.

## 4.4 Patient Pre-Registration Requirements

**Informed consent:** the patient (or guardian, where institutional policy and IRB of record allow) must be aware of the neoplastic nature of his/her disease and willingly consent after being informed of the procedure to be followed, the experimental nature of the therapy, alternatives, potential benefits, side-effects, risks, and discomforts. Current human protection committee approval of this protocol and a consent form is required prior to patient consent and registration.

Eligibility of patients as outlined in [Section 3.2](#Sec3p2) of the protocol should be confirmed prior to pre-registration.

After the patient has been pre-registered the staging scans should be sent to the IROC Ohio Alliance Imaging Core Lab (ICL), per [Section 6.4](#Sec6p4). Registration must occur within 21 days of image submission.

## 4.5 Patient Registration/Randomization Requirements

**Local review of case:** Patient case must be evaluated by a medical oncologist, surgeon and radiation therapist prior to registration.

**Central Review Confirmation:** The Alliance Imaging Core Lab at IROC Ohio will notify the pre-registering site, within 5 business days of receipt, whether or not the patient is eligible based on the central imaging review. IROC Ohio will send an email confirmation to the pre-registering site whether the patient is eligible, and will also carbon copy the Alliance Registration office. The Registration office will open the gate for the patient registration to proceed. The radiology reviewer will enter the central review results into Rave.

**Patient completed booklets:** Patient questionnaire booklets are to be ordered prior to the registration of any patients. Patient questionnaire booklets can be ordered by downloading and completing the CTSU supply request form (located under the site registration documents section of the A021501 CTSU site) and faxing the form to the CTSU data operations center at 1-888-691-8039. Samples of the booklets are found starting on the second page of [Appendix I](#AppI) which are to be used for reference and IRB submission only. They are not to be used for patient completion. There is no booklet for the Registration QOL/Mental Well-being/Physical Well-being/Fatigue assessment (first page of [Appendix I](#AppI)). If needed, the first page of [Appendix I](#AppI) can be adapted to use as a source document.

## 4.6 Patient Pre-Registration and Registration/Randomization Procedures

Patient enrollment will be facilitated using the Oncology Patient Enrollment Network (OPEN). OPEN is a web-based registration system available on a 24/7 basis. To access OPEN, the site user must have an active CTEP-IAM account (check at < <https://eapps-ctep.nci.nih.gov/iam/index.jsp> >) and a 'Registrar' role on either the LPO or participating organization roster.

All site staff will use OPEN to enroll patients to this study. It is integrated with the CTSU Enterprise System for regulatory and roster data and, upon enrollment, initializes the patient in the Rave database. OPEN can be accessed at <https://open.ctsu.org> or from the OPEN tab on the CTSU members’ side of the website at <https://www.ctsu.org>. A user manual is available for OPEN users on the CTSU site.

Prior to accessing OPEN, site staff should verify the following:

- All eligibility criteria have been met within the protocol stated timeframes.
- All patients have signed an appropriate consent form and HIPAA authorization form (if applicable).

Note: The OPEN system will provide the site with a printable confirmation of registration and treatment information. Please print this confirmation for your records.

To receive site reimbursement for specific tests and/or bio-specimen submissions, completion dates must be entered in the OPEN Funding screen post registration. Please refer to the protocol-specific funding page on the CTSU members’ website for additional information. Timely entry of completion dates is recommended as this will trigger site reimbursement.

Further instructional information is provided on the OPEN tab of the CTSU members’ side of the CTSU website at <https://www.ctsu.org> or at <https://open.ctsu.org>. For any additional questions contact the CTSU Help Desk at 1-888-823-5923 or [ctsucontact@westat.com](mailto:ctsucontact@westat.com).

## 4.7 Registration to Correlative and Companion Studies

4.7.1 Registration to Substudies described in [Section 14.0](#Sec14p0)

There are 2 substudies within Alliance A021501. These correlative science studies must be offered to all patients enrolled on Alliance A021501 (although patients may opt to not participate). These substudies do not require separate IRB approval. These substudies included within Alliance A021501 are:

• Alliance A021501-PP1, Pharmacogenetic studies in Alliance A021501 ([Section 14.1](#Sec14p1))

• Alliance A021501-IM1, Imaging science studies in Alliance A021501 ([Section 14.2](#Sec14p2))

If a patient answers “yes” to “I agree to have my specimen collected and I agree that my specimen sample(s) and related information may be used for the laboratory study(ies) described above,” they have consented to participate in the substudies described in [Section 14.1](#Sec14p1). The patient should be registered to Alliance A021501-PP1 at the same time they are registered to the treatment trial (A021501). Samples should be submitted per [Section 6.2](#Sec6p2).

If a patient answers “yes” to “I agree have my scan images be collected and submitted for the research stated above,” they have consented to participate in the substudy described in [Section 14.2](#Sec14p2). The patient should be registered to Alliance A021501-IM1 at the same time they are registered to the treatment trial (A021501). Images should be submitted per [Section 6.4](#Sec6p4).

## 4.8 Stratification Factors and Treatment Assignments

ECOG PS: 0 vs. 1

# 5.0 Study Calendar

The pre-study intervals are guidelines only. Laboratory and clinical parameters during treatment are to be followed using individual institutional guidelines and the best clinical judgment of the responsible physician. It is expected that patients on this study will be cared for by physicians experienced in the treatment and supportive care of patients on this trial.

**Pre-Study Testing Intervals**

• To be completed ≤ 21 DAYS before registration: All laboratory studies, history and physical.

• To be completed ≤ 28 DAYS before registration: CT/MRI scans used for staging

|  | **Prior to Pre-Reg** | **Prior to Reg*** | **Day 1 of each cycle of mFOLFIR-INOX*** | **RT (day 1 to day 5) (Arm 2 only)** | **Surgery** | **Day 1 of each cycle of FOLFOX *** | **Post Tx**  **Follow- up**** |
| --- | --- | --- | --- | --- | --- | --- | --- |
| **Tests & Observations** |  |  |  |  |  |  |  |
| History and physical, weight, PS *** |  | X (1) | X (1) | X (1) |  | X (1) | X (1) |
| Pulse, BP |  | X (1) | X (1) | X (1) |  | X (1) | X (1) |
| Height |  | X (1) |  |  |  |  |  |
| Adverse Event Assessment (CTCAE) † |  | X (1) | X (1) | X (1) | D (1) | X (1) |  |
| Adverse Event Assessment (PRO-CTCAE) †† |  | X (2) | X | X | D | X |  |
| Registration QOL/Mental Well-being/Physical Well-being/Fatigue |  | X (2) |  |  |  |  |  |
| **Laboratory Studies** |  |  |  |  |  |  |  |
| CBC, Differential, Platelets |  | X | X | X | X | X | X |
| Chemistry (Serum Creatinine, Electrolytes, AST, ALT, Alk. Phos., Albumin, Total Bilirubin) |  | X | X | X | X | X | X |
| Pregnancy Test (#) |  | X |  |  |  |  |  |
| CA 19-9 |  | A | A | A | A | A | X |
| **RT Planning** |  |  |  |  |  |  |  |
| EUS with Fiducial Placement for RT |  |  |  | B |  |  |  |
| **Staging** |  |  |  |  |  |  |  |
| Staging CT Scan of Chest or Chest X-ray/CT or MRI of Abdomen | X (3) |  | C (3) | C (3) | C (3) | C (3) | X (3) |
| Central Radiographic Review |  | C |  |  | C |  |  |
| **Central Pathology Review** |  |  |  |  | X (4) |  |  |
| **Optional Correlative studies: For patients who consent to participate** | | | | | | | |
| Blood specimen sample (A021501-PP1) | Between Registration and C1D1. See [Section 6.2](#Sec6p2). | | | | | | |
| Imaging (A021501-IM1) | See [Section 6.4.2](#Sec6p4p2) for CT images submission time points and requirements. | | | | | | |

* Labs completed prior to registration may be used for day 1 of cycle 1 if obtained ≤ 7 days prior to treatment (except pregnancy test and CA 19-9, as detailed below). For subsequent cycles, labs, tests and observations may be obtained +/- 3 days from scheduled day of assessment. Radiographic windows are +/- 7 days from scheduled day of assessment.

** After off-treatment (evaluation of the last treatment cycle), patients will have physical examinations, labs, and staging scans every 16 weeks (+/- 28 days) until they have reached 24 months post-registration or until documented progression, whichever occurs first. Thereafter, survival information is required every 6 months for 5 years post-registration. For patients who discontinue treatment for progressive disease or are removed from protocol treatment, survival information is required every 6 months for 5 years post-registration. See [Section 12.0](#Sec12p0) for removal of patients from protocol therapy.

*** Drug dosages need not be changed unless the calculated dose changes by ≥ 10%.

1. May be performed by physician, NP, or PA responsible for oncologic care of the patient.
2. To be completed after registration and ≤ 21 days prior to treatment. See [Appendix I](#AppI).

3 Chest scans must be CT or chest X-ray. Abdominal baseline and restaging scans can include either a CT or MRI, although CT is preferred. The same method of scanning used at baseline must be used for all subsequent evaluations. The CT must be acquired with 3 mm or less slice thickness. See [Section 7.4.1](#Sec7p4p1) for further details. Supporting documentation is to be submitted, per [Section 6.1.1](#Sec6p1p1). The baseline scan and restaging scan after completion of preoperative therapy/prior to surgery are to be centrally reviewed by the Alliance ICL at IROC Ohio, per [Section 7.4.2](#Sec7p4p2).

4 Central Pathology review is retrospective. Sites must submit slides within 60 days of surgery of patient. See [Section 6.2](#Sec6p2).

† Solicited AEs are to be collected starting at baseline. Routine AEs are to be collected starting after registration. See [Section 9.1](#Sec9p1) for the list of solicited AEs. See [Section 9.4](#Sec9p4) for expedited reporting of SAEs. See [Section 9.2](#Sec9p2) for reporting of surgical AEs, to be completed within 90 days after surgery.

†† Patients complete PRO-CTCAE by paper booklet ordered through the CTSU website. See [Section 9.1](#Sec9p1) for administration instructions. See [Appendix I](#AppI) for PRO-CTCAE assessments for IRB submission and review only. See [Section 4.5](#Sec4p5) for ordering instructions. PRO-CTCAE booklets should be administered at the following time points: ≤ 21 days prior to treatment; day 1 of each cycle of mFOLFIRINOX (+/- 3 days); RT days 1-5 (Arm 2 only); prior to surgery (+/- 7 days); and day 1 of each cycle of FOLFOX (+/- 3 days).

# For women of childbearing potential (see [Section 3.3.6](#Sec3p3p6)). Must be done ≤ 7 days prior to registration.

A CA19-9 may be performed < 28 days prior to registration. Subsequently, CA 19-9 may be performed +/- 14 days from the scheduled date. During treatment, CA 19-9 should be performed every 28 days. For patients who have normal CA 19-9 levels at baseline, continued testing of CA 19-9 is not required.

B EUS/fiducial marker placement is mandatory for patients in the RT arm. Immediately following review of first restaging studies, planning for EUS/fiducial placement and RT simulation should be scheduled to be performed during either cycle 5 or 6 of mFOLFIRINOX.

C Restaging scans should be performed for both Arm 1 and Arm 2 at the following time points: 1. After the first 4 cycles of mFOLFIRINOX; 2. Prior to Surgery; 3. Post-surgery but prior to first cycle of FOLFOX; 4. After 4 cycles while on FOLFOX. The baseline and restaging scan after completion of preoperative therapy and prior to surgery are to be centrally reviewed by the Alliance ICL at IROC Ohio per [Section 7.4.2](#Sec7p4p2). After protocol treatment, scans should be performed per the schedule indicated by footnote “**”

D Surgery-related AEs should be assessed and captured within 90 days of surgery.

# 6.0 Data and Specimen Submission

## 6.1 Data Collection and Submission

Data collection for this study will be done exclusively through the Medidata Rave clinical data management system. Access to the trial in Rave is granted through the iMedidata application to all persons with the appropriate roles assigned in Regulatory Support System (RSS). To access Rave via iMedidata, the site user must have an active CTEP-IAM account (check at https://eapps-ctep.nci.nih.gov/iam/index.jsp) and the appropriate Rave role (Rave CRA, Read-Only, Site Investigator) on either the LPO or participating organization roster at the enrolling site.

Upon initial site registration approval for the study in RSS, all persons with Rave roles assigned on the appropriate roster will be sent a study invitation e-mail from iMedidata. To accept the invitation, site users must log into the Select Login (https://login.imedidata.com/selectlogin) using their CTEP-IAM user name and password, and click on the “accept” link in the upper right-corner of the iMedidata page. Please note, site users will not be able to access the study in Rave until all required Medidata and study specific trainings are completed. Trainings will be in the form of electronic learnings (eLearnings), and can be accessed by clicking on the link in the upper right pane of the iMedidata screen.

Users who have not previously activated their iMedidata/Rave account at the time of initial site registration approval for the study in RSS will also receive a separate invitation from iMedidata to activate their account. Account activation instructions are located on the CTSU website, Rave tab under the Rave resource materials (Medidata Account Activation and Study Invitation Acceptance). Additional information on iMedidata/Rave is available on the CTSU members’ website under the Rave tab at www.ctsu.org/RAVE/ or by contacting the CTSU Help Desk at 1-888-823-5923 or by e-mail at ctsucontact@westat.com.

A Schedule of Forms is available on the Alliance study webpage, within the Case Report Forms section. The Schedule of Forms is also available on the CTSU site within the study-specific Education and Promotion folder, and is named Time & Events.

6.1.1 Supporting documentation

This study requires supporting documentation for diagnosis, response, and progression. Supporting documentation will include radiology, pathology, and surgical operation reports; these must be submitted at the following time points:

- **Baseline:** Imaging report, pathology/cytology report, clinic note
- **Completion of preoperative therapy**: Imaging report
- **Resection:** Operative report, pathology report
- **Response:** Imaging report
- **Progression/relapse/recurrence:** Imaging report, and pathology report if applicable

## 6.2 Specimen Collection and Submission

For all patients registered to Alliance A021501: Retrospective histopathology review will be conducted using a representative paraffin embedded block of pancreatic tumor from the surgical resection specimens. If the surgical resection specimen does not show residual cancer, the block from the diagnostic biopsy or representative FNA cytologic slide will also be acceptable. **The submission of tumor (or diagnostic pathology for patients that have a pCR at surgery) for histopathology review is required for all patients registered to this study, including those who are found to be ineligible and those who do not receive protocol therapy.**

For patients registered to substudy A021501-PP1: All participating institutions must ask patients for their consent to participate in the correlative substudies planned for Alliance A021501-PP1, although patient participation is optional. Biomarker and pharmacogenetic studies will be performed. Rationale and methods for the scientific components of this study is described in [Section 14.1](#Sec14p1).

For patients who consent to participate, specimens will be collected at the time points listed in the table below:

|  | **Prior to Initiation of Treatment*** | **At surgery** | **Storage/ Shipping** | **Submit to:** |
| --- | --- | --- | --- | --- |
| **Mandatory for all patients on A021501:** | | | | |
| **Paraffin block OR 10 unstained slides for histopathology review** | X | X** | Ambient temp/ ship over night | WUSTL |
| **To be submitted ONLY for patients that consent to A021501-PP1***:** | | | | |
| **Whole Blood^1^**  (EDTA/lavender top) | 1 x 10 mL |  | Cool pack/ship over night | WUSTL |

* To be collected any time after registration but prior to the initiation of treatment

** Required ONLY in patients with pathologic CR or histology other than adenocarcinoma at time of surgery. See [Section 6.2.2](#Sec6p2p2) for submission requirements.

*** Patients who consent to A021501-PP1 are those who answer “Yes” to the model consent question, “I agree to have my blood specimen collected and I agree that my specimen samples and related information may be used for the laboratory studies described above.”

1 For patients who consent to correlative study A021501-PP1, whole blood is to be used for pharmacogenomic analyses described in [Section 14.1](#Sec14p1). This tube of blood is preferred to be collected prior to the initiation of treatment but can be collected at any time while a consented patient is on study.

6.2.1 Specimen submission using the Alliance Biospecimen Management System

USE OF THE ALLIANCE BIOSPECIMEN MANAGEMENT SYSTEM (BioMS) IS MANDATORY AND ALL SPECIMENS MUST BE LOGGED AND SHIPPED VIA THIS SYSTEM.

BioMS is a web-based system for logging and tracking all biospecimens collected on Alliance trials. Authorized individuals may access BioMS at the following URL: http://bioms.allianceforclinicaltrialsinoncology.org using most standard web browsers (Safari, Firefox, and Internet Explorer). For information on using the BioMS system, please refer to the ‘Help’ links on the BioMS webpage to access the on-line user manual, FAQs, and training videos. To report technical problems, such as login issues or application errors, please contact: 1-855-55-BIOMS or Bioms@alliancenctn.org. For assistance in using the application or questions or problems related to specific specimen logging, please contact: 1-855-55-BIOMS or Bioms@alliancenctn.org.

After logging collected specimens in BioMS, the system will create a shipping manifest. This shipping manifest must be printed and placed in the shipment container with the specimens.

All submitted specimens must be labeled with the protocol number (A021501), Alliance patient number, patient’s initials, date and type of specimen collected (e.g., serum, whole blood).

A copy of the Shipment Packing Slip produced by BioMS must be printed and placed in the shipment with the specimens.

Instructions for the collection of specimens are included below. Please be sure to use a method of shipping that is secure and traceable. Extreme heat precautions should be taken when necessary.

Ship specimens on Monday through Thursday only. Shipping by overnight service to assure receipt is encouraged. Do not ship specimens on Fridays or Saturdays [unless lab specifies Saturday delivery].

All specimens should be sent to the following address:

Alliance Biorepository at Washington University (WUSTL)

BJC Institute of Health

425 S. Euclid Ave.

Room 5120

St. Louis, MO 63110-1005

Tel: 314-454-7615 Fax: 314-454-5525

tbank@wudosis.wustl.edu

6.2.2 Tissue collection and processing for histopathology review

Consistent and accurate histologic grading is important for this study. Submission of tumor paraffin blocks, cores, or slides (see below) from the patient’s resection is required for all patients enrolled to this study. If the resection specimen shows pCR, then submission of the pretreatment/diagnostic fine needle aspiration (FNA) or core biopsy is also necessary.

The pretreatment diagnosis of pancreatic adenocarcinoma will be established by fine needle aspiration (FNA) cytology or core needle biopsy. All patients must undergo local pathology review of the biopsy for confirmation of diagnosis prior to registration. Central pathology review of the pretreatment specimen is not required prior to registration. It is anticipated that the pretreatment diagnosis of pancreatic adenocarcinoma will be established by fine needle aspiration (FNA) cytology in most cases. Less commonly, core needle biopsy may have been performed for initial diagnosis. All patients will undergo local pathology review of the biopsy for confirmation of diagnosis.

Following protocol surgery, in the event that the surgical pathology report of the resected tumor demonstrates no pathologic evidence of malignancy (i.e., pathologic complete response; pCR) or an unexpected histology other than adenocarcinoma, the pretreatment diagnostic cytology/pathology specimens and representative slides of the resected post-treatment specimen will be requested and reviewed at The Ohio State University by the pathology co-chair.

The following materials, derived from formalin fixed, paraffin embedded diagnostic and surgical pathology tissue blocks are required for central pathology review when the resection specimen shows pCR:

- one stained representative slide of the pretreatment FNA containing unequivocal adenocarcinoma

or

- core needle biopsy containing unequivocal adenocarcinoma

The de-identified surgical pathology report, coded with the Alliance patient ID number, must accompany all tissue specimen submissions. Usually, this is generated by obscuring all PHI (names and dates) with white-out or a black magic marker, labeling each page of the report with the Alliance patient ID, and photocopying the report.

For patients who consent to future unspecified research (model consent question, “My samples and related information may be kept in a Biobank for use in future health research”) any unused tumor tissues from histology review will be stored at the Alliance biorepository for later use.

Paraffin blocks of primary diagnostic and, when available, metastatic tissue obtained from archival pancreatic tumor specimens should be sent to the Alliance Biorepository at Washington University (WUSTL). Please specify the source of the tumor block (primary or metastatic site).

The Alliance has instituted special considerations for the small percentage of hospitals whose policy prohibits long-term storage of blocks, and the smaller percentage of hospitals whose policies prohibit release of any block. If, due to institutional policy, a block cannot be sent, one stained and 10 unstained slides (thickness of 4-5 micron) will be sent to the Alliance Biorepository (WUSTL).

The goal of the Alliance Biorepository is to provide investigators with quality histology sections for their research while maintaining the integrity of the tissue. All paraffin blocks that are to be stored at the Alliance Biorepository will be vacuum packed to prevent oxidation and will be stored at 4º C to minimize degradation of cellular antigens. For these reasons it is preferred that the Alliance Biorepository bank the block until the study investigator requests thin sections. Please contact the Alliance Biorepository if additional assurances with your hospital pathology department are required.

6.2.3 Blood specimen submission for A021501-PP1

For patients who consent to participate, whole blood specimen will be used for the pharmacogenomic studies described in [Section 14.1](#Sec14p1). This specimen should be collected prior to the initiation of protocol treatment.

Collect 10 mL of peripheral venous blood in an EDTA (lavender) tube. The tubes should be inverted several times to mix the EDTA and refrigerated until shipped on cool pack by overnight mail to the Alliance Biorepository at Washington University (WUSTL). The specimens should be shipped the same day that the blood is drawn in ambient temperature per [Section 6.2.1](#Sec6p2p1).

Label specimens with the following identification:

1. Procurement date
2. Alliance patient number
3. Alliance study number (i.e., A021501-PP1)
4. Whole Blood

Ship blood per instructions in [Section 6.2.1](#Sec6p2p1).

## 6.3 Submission of Digital Radiation Therapy Data

6.3.1 Digital RT Data Submission Requirements

Submission of treatment plans in digital format as DICOM RT is required. Digital data must include CT scans, structures, plan and dose files. This study uses TRIAD for RT data submission. Use of TRIAD requires several preliminary steps. See [Section 4.2.1](#Sec4p2p1) for details. Additional information is available at: http://triadhelp.acr.org/ClinicalTrials/NCISponsoredTrials.aspx

In the event that a site has not completed all steps required for TRIAD data submission in time to meet the timeline for pre-treatment review, data submitted via SFTP will also be accepted. See the instructions for submission of data via SFTP on the IROC Rhode Island website under Digital Data.

Any items on the list below that are not part of the digital submission may be included with the transmission of the digital RT data.

One week prior to the start of radiotherapy, the following data shall be submitted for pre-treatment review:

6.3.1.1 Treatment Planning System Output

RT treatment plans including CT, structures, dose and plan files. These items are included in the digital plan.

Dose volume histograms (DVH) for the composite treatment plan for all target volumes and required organs at risk. For all RT plans, a DVH shall be submitted for a category of tissue called “unspecified tissue.” This is defined as tissue contained within the skin, but which is not otherwise identified by containment within any other structure. DVHs are included in the digital plan.

Digitally reconstructed radiographs (DRR) for each treatment field, showing the collimator and beam aperture. Please include two sets, one with and one without overlays of the target volumes and organs at risk. Please have the fiducial markers included in the DRRs that are used for set-up.

Treatment planning system summary report that includes the monitor unit calculations, beam parameters, calculation algorithm, and volume of interest dose statistics.

6.3.1.2 Supportive Data

The following data is to be submitted to IROC Rhode Island:

- - All diagnostic imaging used to define the target volume.
  - Prescription sheet for entire treatment.
  - If the recommended doses to the organs at risk are exceeded, an explanation should be included for review by IROC Rhode Island and the radiation oncology reviewers.

6.3.1.3 Forms

The following forms are to be submitted to IROC Rhode Island:

- RT-1 Dosimetry Summary Form
- Motion Management Reporting Form
- Within 21 days of the completion of radiotherapy, the following data shall be submitted for all patients:
- The RT-2 Radiotherapy Total Dose Record Form
- A copy of the patient's radiotherapy record including the prescription, and the daily and cumulative doses to all required areas.
- Documentation listed above showing any modifications from the original submission.

Supportive data and forms may be included with the transmission of the digital RT data or submitted separately via e-mail (DataSubmission@qarc.org) or mailed to:

IROC Rhode Island QA Center

640 George Washington Highway, Building B, Suite 201

Lincoln, Rhode Island 02865-4207

Phone: (401) 753-7600

Fax: (401) 753-7601

Questions regarding the dose calculations or documentation should be directed to:

Protocol Dosimetrist

IROC Rhode Island QA Center

640 George Washington Highway, Building B, Suite 201

Lincoln, Rhode Island 02865-4207

Phone: (401) 753-7600

Fax: (401) 753-7601

## 6.4 Imaging Requirements, Credentialing and Submission Instructions

6.4.1 Institutional Imaging Credentialing Procedures

Prior to the enrollment of patients on A021501, institutions must be credentialed by the Alliance Imaging Core Laboratory (ICL) at The Ohio State University Medical Center (also known as IROC Ohio). If the site is already credentialed by the ICL to participate in imaging studies, the ICL will provide a brief A021501 protocol refresher prior to the site enrolling patients on this trial.

See [Section 15.1.1](#Sec15p1p1) for further details

6.4.2 CT Submission Instructions

The following images and local interpretation reports will be collected digitally and submitted for centralized, **real-time re-review** (in addition for the A021501-IM1 correlative study and for banking purposes)**:**

- Baseline (within 28 days prior to patient registration): This scan will be reviewed centrally to ensure that patient meets the criteria for borderline resectable disease, as detailed in [Section 3.2.1](#Sec3p2p1) and [Appendix II](#AppII).
- Pre-Surgery restaging:

1. Arm 1 (after 8 cycles of mFOLFIRINOX and prior to surgery).
2. Arm 2 (after 7 Cycles of mFOLFIRINOX + Radiotherapy and prior to surgery)

This scan will be reviewed centrally to determine if the patient has potentially resectable disease and is appropriate for surgical intervention, at completion of preoperative therapy.

For patients who consent to the imaging study A021501-IM1 (model consent question, “I agree to have my scan images be collected and submitted for the research stated above”), the following scans and local interpretation reports should also be submitted to the IROC Ohio Imaging Core Lab (ICL) for the A021501-IM1 correlative study and banking purposes. These scans may be batched shipped every 6 months:

- Post 4 cycles of mFOLFIRINOX (See [Section 5.0](#Sec5p0))
- Post-surgery restaging (Post-Surgery and prior to first cycle of FOLFOX)
- Post 4 Cycles of FOLFOX
- Post-Treatment Follow-Ups (every 16 weeks +/- 28 days after completion of treatment for 24 months post-registration or until documented progression, whichever occurs first)

All scans must be submitted digitally. CD/DVD shipment of images is **not** allowed.

6.4.2.1 Real-time review scan submission and timeframes

The complete CT/MRI scan in digital **DICOM** format will be submitted to Alliance Imaging Core Laboratory at IROC Ohio within no more than 3 business days after acquisition completeness or pre-registration. BMP files, JPG files, or hard copies (films) are not acceptable. The raw data of the entire study should be saved until the scan is accepted by the Imaging Core Laboratory. The Imaging Core Lab will notify site and Alliance A021501 imaging committee within **2 business days** of the data receipt as well as within **3 business days** of the quality check report upon data receipt.

Sites need to de-identify the patient data using institutional procedures to remove patient name and medical record number while preserving the Alliance patient ID number (e.g., 112136) and protocol number (e.g., A021501), respectively.

For baseline staging and pre-surgical restaging CT/MR scans, imaging data must be submitted to the Imaging Core Lab **electronically** via TRIAD, Web-based data transfer, or FTP data transfer approaches for real-time central review purposes. For patients that consent to A021501-IM1, CT/MRI images will be collected digitally for the research described in [Section 14.2](#Sec14p2).

TRIAD data transfer: See [Section 4.2.1](#Sec4p2p1) for details. Additional information is available at http://triadhelp.acr.org/ClinicalTrials/NCISponsoredTrials.aspx

6.4.2.2 Web-based data transfer

Any PCs with Internet access can be used to upload images to the Imaging Core Lab via this approach. The standard Web access information will be provided separately through the specific trial e-mail Alliance021501@irocohio.org, per the request by participating sites before their first data submission.

6.4.2.3 FTP Transfer

Any FTP software can be used to upload images to the secure FTP Server of the Imaging Core Laboratory. The standard FTP access information will be provided separately through the specific trial e-mail Alliance021501@irocohio.org, per the request by participating sites before their first data submission.

De-identified local scan interpretation reports must be sent along with all image submissions. These de-identified reports can be sent to the ICL at IROC Ohio via Alliance021501@IROCOhio.org or Fax at (614) 293-9275.

Send an e-mail notification to inform the Imaging Core Lab at Alliance021501@irocohio.org of the imaging data submission once the data transfer is complete. Any questions or problems about the data transfer to the Imaging Core Lab, call the Core Lab IT group at 614-293-2630 or 614-366-4932 for help.

Please note, institutions located in states with dosimetry reporting requirements specified by state law must include dosimetry data in the final imaging report.

# 7.0 Treatment Plan/Intervention

Protocol treatment is to begin ≤ 14 days of registration.

For questions regarding treatment, please see the study contacts page.

It is acceptable for individual chemotherapy doses to be delivered ≤ a 24-hour (business day) window before and after the protocol-defined date for Day 1 of a new cycle. For example, if the treatment due date is a Friday, the window for treatment includes the preceding Thursday through the following Monday. In addition, patients are permitted to have a new cycle of chemotherapy delayed up to 7 days for major life events (e.g., serious illness in a family member, major holiday, vacation that cannot be rescheduled) without this being considered a protocol violation. Documentation to justify this delay should be provided.

Surgery and imaging will be conducted at the registering institution. Radiation therapy must be performed at a radiation therapy facility participating in the IROC monitoring program. Chemotherapy may be given at a non-registering institution. All protocol conduct must be followed and the registering institution is responsible for ensuring all data is reported per protocol. Please refer to the Alliance policy and procedures document posted on the Alliance website for the policy on engagement in research by non-registering institutions. If the NCTN Group credited for enrollment is a non-Alliance Group, then other requirements from the credited Group may apply.

Patients will be randomized to either Arm 1 or Arm 2 of the study. Patients will progress through treatment in the absence of any criteria described in [Section 12.1.3](#Sec12p1p3).

Patients on both arms A and Arm 2 will receive 4 cycles of mFOLFIRINOX and then undergo restaging imaging studies, as per [Section 7.4.1](#Sec7p4p1). These scans are NOT centrally reviewed.

After the first set of restaging scans and patients do not show progression on the local read per [Section 12.1.3](#Sec12p1p3), patients on Arm 1 will receive 4 additional cycles of mFOLFIRINOX. Patients on Arm 2 will receive 3 additional cycles of mFOLFIRINOX and then hypofractionated radiotherapy per [Section 7.2](#Sec7p2). These additional cycles of mFOLFIRINOX for both Arm 1 and Arm 2 should start within 14 days after cycle 4 day 1 unless dose modifications indicate delay.

After preoperative therapy is completed, patients will be restaged and, if still considered a surgical candidate for resection per the criteria of [Section 3.2.1](#Sec3p2p1) after central review, will proceed to surgery per [Section 7.3](#Sec7p3). Surgery must occur 4 to 8 weeks after last dose chemotherapy (Arm 1) or of radiation (Arm 2).

Patients considered inappropriate for surgery will go off of study treatment, and are to be followed per the follow-up schedule provided in the study calendar ([Section 5.0](#Sec5p0)).

Following surgery, patients will receive post-operative chemotherapy with 4 cycles of FOLFOX starting 4 to 12 weeks from date of surgery.

Treatment will continue until disease progression or unacceptable adverse event or inability to tolerate treatment even with all allowed dose reductions, or completion of the full treatment program and entrance onto clinical follow-up. Also, see Sections [12.1.3](#Sec12p1p3) and [12.3](#Sec12p3) for additional information regarding discontinuation of treatment.

See [Section 8.1](#Sec8p1) for ancillary/concomitant therapy guidelines.

## 7.1 Chemotherapy (Arm 1 and Arm 2)

7.1.1 Neoadjuvant mFOLFIRINOX

Arm 1: 8 Cycles of mFOLFIRINOX

Arm 2: 7 Cycles of mFOLFIRINOX

1 cycle = 14 days

A cycle of mFOLFIRINOX is delivered as follows:

- **Oxaliplatin**: 85 mg/m^2^ IV over 2 hours on day 1, followed by,
- **Irinotecan**: 180 mg/m^2^ IV over 90 minutes on day 1, followed by,
- **Leucovorin***: 400 mg/m^2^ IV over 2 hours on day 1, followed by,
- **5-FU****: 2400 mg/m^2^ IV over 46-48 hours.

*Alternatively, leucovorin may be administered concurrently with the last 30 min of oxaliplatin, and the entire 90 minutes of irinotecan.

**5-FU is administered via IV infusion only; there is no bolus injection of 5-FU.

White blood cell growth factor is required within 24 hours of completion of 5-FU, administered according to institutional procedures. See [Section 8.1.9](#Sec8p1p9) for further details.

Table S2. Neoadjuvant mFOLFIRINOX dosing

| **Agent** | **Dose** | **Route** | **Day** | **ReRx** |
| --- | --- | --- | --- | --- |
| Oxaliplatin | 85 mg/m^2^ | IV | Day 1 | every 2 weeks |
| Irinotecan | 180 mg/m^2^ | IV | Day 1 | every 2 weeks |
| Leucovorin | 400 mg/m^2^ | IV | Day 1 | every 2 weeks |
| Fluorouracil | 2,400 mg/m^2^ | IV | Days 1-2 | every 2 weeks |

See [Section 7.4](#Sec7p4) for restaging scan imaging requirements and [Section 7.3](#Sec7p3) for details on surgery.

7.1.2 Adjuvant FOLFOX

Arm 1 or Arm 2: 4 Cycles of FOLFOX

1 cycle = 14 days

FOLFOX is to be initiated 4 to 12 weeks from date of surgery.

A cycle of FOLFOX is delivered as follows:

- **Oxaliplatin**: 85 mg/m^2^ IV over 2 hours on day 1, followed by,
- **Leucovorin**: 400 mg/m2 IV over 2 hours (Alternatively, leucovorin may be administered (via separate infusion containers) concurrently with oxaliplatin), on day 1, followed by,
- **5-FU**: 400mg/m2 IV bolus, then 2400 mg/m2 continuous IV over 46-48 hours.

Table S3. Adjuvant FOLFOX dosing

| **Agent** | **Dose** | **Route** | **Day** | **ReRx** |
| --- | --- | --- | --- | --- |
| Oxaliplatin | 85 mg/m^2^ | IV | Day 1 | every 2 weeks |
| Leucovorin | 400 mg/m^2^ | IV | Day 1 | every 2 weeks |
| Fluorouracil | 400 mg/m^2^ bolus then  2,400 mg/m^2^ | IV | Days 1-2 | every 2 weeks |

## 7.2 Radiotherapy – Arm 2 Only

*An optional, yet recommended, short online tutorial for radiation planning and treatment is available for review.*

- Go to the EduCase website – <http://www.educase.com>
- Register on educase.com by clicking the “Login” tab, “Don’t have an account?” link, and complete the required registration information.
- Once logged in, navigate to EduCase > Case Listing. In the right-hand menu, navigate to: EduCase > Events > Alliance
- The password is “borderline”
- On the Alliance Case Listing page will be all of the links to optional video tutorials for physicians (treatment planning), dosimetry, physics, and therapists.
- There is also a practice case to review contouring of anatomy.

Patients will receive either:

- Stereotactic body radiation therapy (SBRT)

or

- Hypofractionated image guided radiation therapy (HIGRT).

**SBRT is strongly preferred, when appropriate**. The following are required for patients to be eligible for SBRT:

- Centers delivering SBRT must be credentialed for SBRT ([Section 15.1.2](#Sec15p1p2)).
- Patients with active duodenal or gastric ulcers are not acceptable for SBRT. Patients with previous ulcers that have resolved (no active bleeding or symptoms) are acceptable for SBRT.
- Patients with direct tumor invasion of the bowel or stomach based on endoscopy are not acceptable for SBRT, but may be considered for HIGRT.
- Patients should not be treated with SBRT if SBRT-specific organ at risk (OAR) constraints cannot be met; these patients should be considered for HIGRT.
- Patients should have 4D CT simulation to assess tumor motion with respiration
- Patients should be treated with SBRT only if tumor motion can be minimized using motion management techniques, when applicable.
- SBRT requires the ability to perform daily image guidance.

**HIGRT** should be considered in the following situations:

1. Centers that are not credentialed for SBRT may treat with HIGRT if appropriately credentialed ([Section 15.1.2](#Sec15p1p2)).
2. Direct tumor invasion of the bowel or stomach on endoscopy is acceptable for HIGRT.
3. Active duodenal or gastric ulcers are not acceptable for HIGRT. Patients with previous ulcers that have resolved are acceptable. This must be confirmed at the time of fiducial placement.

**Regardless whether SBRT or HIGRT is used this protocol requires pre-treatment review of the contours and plan PRIOR TO DELIVERY of radiation treatment for all patients. See** [**Section 6.3**](#Sec6p3) **for pre-treatment review requirements. See** [**Section 15.1.2**](#Sec15p1p2) **for the credentialing requirements for this study.**

7.2.1 Radiotherapy technical factors and treatment planning

- Only ≥6 MV photons are permitted for HIGRT and SBRT.
- Particle therapy is not permitted.

**Fiducial marker placement must be attempted for all patients receiving HIGRT and SBRT as described below:**

- 1-5 (preferably ≥3) fiducial markers should be placed for targeting purposes. Example markers include Civco, Visicoil, and Gold anchor (See [Appendix V](#AppV) for ordering info).
- The fiducial markers will be placed in or directly at the tumor periphery and/or within 1 cm of the tumor (normal pancreas) under endoscopic ultrasound (preferred) or CT guidance.
- Fiducials may be implanted prior to enrollment or during a planned research biopsy as this is an acceptable standard of care procedure for any patient receiving RT for localized pancreatic or periampullary cancer. If fiducials are not placed prior to enrollment, it is recommended that they be placed during either cycles 5 or 6 of FOLFIRINOX.
- The fiducial markers will be used as surrogates for targeting the daily tumor position during treatment.
- In the rare event when fiducials cannot be placed, the patient can receive HIGRT or chemotherapy alone per local PI discretion.

CT simulation is done in the supine position with a customized immobilization device.

- Simulation should not be done on the same day as fiducial marker placement. An interval of at least 12 hours is required between fiducial marker placement and simulation.
- Patients will be positioned supine, arms above the head, in an Alpha Cradle or equivalent immobilization device that will be custom-made for each patient.
- Administration of IV and oral contrast is required for target and normal tissue delineation unless patient has a contrast allergy refractory to premedication or at the discretion of the treating physician. A pretreatment renal scan to assess kidney differential will be performed prior to initiation of treatment as clinically appropriate.
- A 4D CT scan (when available) will be performed to assess respiratory motion. If 4D CT scan is not available then fluoroscopy on the treatment machine or simulator can be used to determine maximal motion based on the fiducial marker. If >5 mm of tumor motion in any direction is noted, then the use of respiratory motion management (i.e. breath-holding technique, respiratory tracking, abdominal compression) is recommended. If ≤ 5mm of tumor motion, respiratory motion management is still recommended, but not required.
- MRI or PET simulation may be performed in the treatment position and later fused to the CT simulation scan to assist in target volume delineation, or if IV contrast cannot be used, but this is not required.

7.2.1.1 HIGRT/SBRT Treatment Planning

See tutorial for examples of contouring the tumor, vessels, and organs at risk (OARS)

Dose Prescription:

- - - - - **SBRT:** A dose of 6.6 Gy 5 fractions will be prescribed to the modified PTV (mPTV). Up to 8 Gy will be allowed to boost the dose to the tumor vessel interface (TVI).
        - **HIGRT:** A dose of 5 Gy x 5 fractions will be prescribed to the PTV.

Target Volumes:

- - Target Volumes Definitions: (see treatment planning section for full details)
    - GTV = gross tumor on free breathing scan
    - PTV = GTV on free breathing scan + 3mm uniform expansion
    - GITV = gross tumor, accounting for motion
    - TVI = tumor vessel interface contoured for each vessel separately at level of tumor
    - TVI expansion (TVIexp) = TVI + 3mm uniform expansion for each vessel involved.
    - Modified PTV (mPTV) = GITV + TVI expansion + 3mm (for SBRT) or 5-10mm (for HIGRT) all subtracted by the OAR expansion (OAR + 3mm)
  - Gross Tumor Volume (GTV):
    - The GTV will be created in the same manner for both SBRT and HIGRT.
    - The GTV will include the primary pancreatic tumor only.
    - The diagnostic CT, respiratory-correlated 4D-CT scan, pancreas protocol CT, MRI, and/or the FDG-PET/CT scan (when available) should be reviewed to facilitate accurate delineation of the GTV.

Gross Internal Target Volume (GITV):

- - For patients who have 4D CT simulation, the GITV will be created in the same manner for both SBRT and HIGRT.
  - GITV will not be created if breath-hold (ABC) or gating is used.
  - The GITV may be determined by either of the following:
    - Contouring the GTV on the “primary” scan (usually the contrast enhanced scan), and then expand the GTV as necessary based on the breathing phases of the 4D-CT (which are pulled in as secondary scans).
    - Contouring the tumor on two extreme phases of breathing cycle (inspiration/expiration) on 4D CT and/or the extreme SI motion of the pancreatic fiducials seen on fluoroscopy or 4D CT.

Tumor-Vessel Interface (TVI):

- - The TVI will be created in the same manner for both SBRT and HIGRT.
  - The TVI will include the segment of portal vein, superior mesenteric vein (SMV), superior mesenteric artery (SMA), common hepatic artery (CHA), and/or celiac artery that is in direct contact with tumor.
  - TVI structures will be contoured to include entire radial extent of any vessel that contacts the tumor. For example, the SMA TVI will include the entire 360-degree extent of SMA at that level even if there is only 90-degree involvement of the SMA by tumor. This radial contour should extend superiorly and inferiorly on each axial slice where there is GTV contoured (see tutorial for full explanation).
  - Review of these contours in the axial, coronal, and sagittal view with a radiologist who has expertise in pancreatic cancer is encouraged.
  - The vessel(s) contoured at the level of the GTV should be uniformly expanded by 3mm to generate the TVI expansion.

Fiducials/Clips:

- - Fiducials and/or clips should be contoured separately starting superiorly and moving inferiorly in numeric order. It is often easier to see them in the bone window.
  - This volume should be expanded 3mm to create “fiducial exp.”
  - Note: If insurance does not cover fiducial placement, HIGRT only will be allowed.

Clinical Target Volume (CTV):

- - Because elective nodal irradiation will NOT be performed for either SBRT or HIGRT, a CTV will NOT be defined.

Planning Target Volume (PTV):

- - **HIGRT:** The modified PTV will be created by a uniform expansion of 5-10 mm from the summed GITV and TVI volumes. A smaller PTV expansion is encouraged based on the utilization of daily image guidance such as cone-beam CT. (modified PTV = GITV + TVI + 3-5mm)
  - **SBRT**: The PTV will be created by a uniform expansion of 3 mm from the summed GITV and TVI volumes. (PTV = GITV + TVI + 3mm)
    - The OARs should be expanded by 3mm. If there is overlap with the PTV, especially the duodenum, small bowel, and/or stomach, then the PTV should be edited out of the expanded OAR structures to form the **modified PTV (mPTV)**. Smoothing of the final modified PTV is allowed if sharp edges are created from the expansion.
  - The final modified PTV is edited by subtracting the OAR + 3mm from the mPTV (see tutorial for full explanation).

Treatment Technique

- - **HIGRT:** 3D conformal radiation therapy (3DCRT), static IMRT, or volumetric arc therapy (VMAT) are all permissible techniques for HIGRT. Flattening Filter Free (FFF) treatment is allowed.
  - **SBRT:** Due to the close proximity of OARs (especially GI luminal structures) to the PTV, static IMRT or VMAT must be used; 3DCRT is not permissible. It is recommended that 6-12 co-planar static IMRT fields or 1-3 arc fields (VMAT/Rapid-Arc) be used in the radiation treatment plan. Flattening Filter Free (FFF) treatment is allowed. CyberKnife is a permissible technique for treatment delivery but must be credentialed individually. See [Section 15.1.2](#Sec15p1p2) for credentialing instructions
  - **HIGRT and SBRT:** HIGRT and SBRT treatment plans will be developed using commercially available systems based on tumor geometry and location. Institutional standards for radiation quality assurance and radiation delivery will be utilized (see [Section 6.3](#Sec6p3) for protocol-specific Quality Assurance Documentation).

Motion Management

- - Motion management using respiratory gating or breath-hold (active breathing control), (Varian, Elekta, Novalis), respiratory tracking (Cyberknife), or abdominal compression can reduce cranial-caudal fiducial marker motion from typically 11-22 mm peak to ≤5 mm.
  - Use of respiratory gating, ABC, or respiratory tracking requires real time cone beam CT, fluoroscopy, or KV imaging to confirm fiducial location during treatment.
  - If tumor/fiducial marker motion is >5 mm despite the use of abdominal compression and/or other motion management techniques listed above are not available, then patients should receive HIGRT instead of SBRT (see [Section 7.2.1](#Sec7p2p1)).
  - The selection of which radiotherapy treatment machine to use is left to each investigator. As long as the specified dosimetric parameters for SBRT are reached, patients may be treated on any IGRT-enabled machine.
  - **HIGRT:** Motion management described above should be used when possible for HIGRT, but is not required. At a minimum daily port films aligned to spine should be performed.
  - **SBRT:** Cranial-caudal tumor motion must be limited to ≤5 mm and motion management techniques should be used to achieve this, when applicable.

Organs at Risk (OARs): Follow the upper abdominal contouring atlas located:https://www.rtog.org/CoreLab/ContouringAtlases/UpperAbdominalNormalOrganContouringConsensusGuidelines.aspx

- The spinal cord, bilateral kidneys, liver, stomach, duodenum, and other small bowel should be contoured carefully. Spleen should also be contoured and added as an OAR, although no formal dose constraint will be used for spleen.
- Radiation dose to OARs must be minimized.
- OAR dose constraints are based on those OARs within 1 cm (all directions of the final modified PTV).
- “Proximal” refers to any OAR within 1 cm of the final modified PTV (mPTV).
- HIGRT constraints
  - Modified PTV = GITV + TVI expansion + 3mm subtracted by OAR expansion
  - A dose of 5 Gy x 5 fractions will be prescribed to the modified PTV
- SBRT constraints
  - - Modified PTV = GITV + TVI expansion + 3mm subtracted by OAR expansion.
    - PTV = GITV fb + 3mm expansion
    - Greater than 95% of the PTV (GTV + 3mm expansion) should receive a minimum of 4 Gy per fraction.
    - Greater than 95% of the modified PTV (mPTV) should receive a minimum of 5 Gy and a maximum of 8 Gy per fraction. The dose to the PTV should be maximized as long as the OAR dose constraints are met. Efforts should be made to focus hot spots and maximize dose to the TVI expansion.
    - If the constraints below cannot be achieved, then 90% of the **GTV** must receive at least 25 Gy and 90% of the tumor vessel interface 30 Gy. **If these constraints cannot be met, the patient should receive HIGRT.**
    - Based on an analysis of duodenal toxicity representing pooled data (Herman et al) from 3 previous prospective studies, the following dose constraints must be met: V15<9cc, V20<3cc.   V15 and V20 are defined as the percent volume receiving at least 15 Gy and 20 Gy, respectively.  No more than 1cc of the duodenum, small bowel, or stomach may exceed 33 Gy.  The remainder of the constraints **will be limited as follows:**

SBRT Required OARs and Constraints:

| **Description** | **Planning System Name** | **Constraints** |
| --- | --- | --- |
| Modified PTV | mPTV | V25 > 95% (range 25-40 Gy) |
| PTV | PTV | V20 > 95% |
| **OAR** |  | **Constraints** |
| Duodenum | Duodenum | V15 <9cc |
|  |  | V20 <3cc |
|  |  | V33 <1cc |
| Small Bowel | Bowel | V15 <9cc |
|  |  | V20 <3cc |
|  |  | V33 <1cc |
| Stomach | Stomach | V15 <9cc |
|  |  | V20 <3cc |
|  |  | V33 <1cc |
| Liver | Liver | V12 <50% |
| Combined Kidneys | Kidneys | V12 <75% |
| Spinal Cord | Spinal­_Cord | V20 <1cc |
| Spleen | Spleen | No constraint |

HIGRT Required OARs and Constraints:

| **Description** | **Planning System Name** | **Constraints** |
| --- | --- | --- |
| Modified PTV | mPTV | Rx dose is 5 Gy x 5  ≥95% mPTV covered by ≥95% Rx dose, 110% max |
| Right Kidney | Kidney_R | 70% one kidney less than 15Gy |
| Left Kidney | Kidney_L | 70% one kidney less than 15Gy |
| Liver | Liver | Mean <15Gy |
| Spinal Cord | Spinal_Cord | 20 Gy max |
| Small bowel | Bowel_Small | 50% <12Gy, 105% max |
| Duodenum | Duodenum | 50% <12Gy, 105% max |
| Stomach | Stomach | 50% <12Gy, 105% max |
| Spleen | Spleen | No constraint |

7.2.1.2 HIGRT/SBRT Treatment Schedule

All patients must start HIGRT or SBRT within 6 weeks of the simulation scan.

7.2.1.3 HIGRT/SBRT Treatment Delivery

Patients will receive 5 once daily fractions of HIGRT or SBRT given over 1-2 weeks. It is preferred that all 5 fractions be delivered in consecutive daily fashion, starting on a Monday and ending on a Friday. It is acceptable for fractions to be delivered every other day, such that at least 2 or 3 fractions per week are delivered.

SBRT: As long as the specified dosimetric parameters for SBRT are reached, patients may be treated on any image-guided (IGRT)-enabled machine.

Initial patient positioning will be based on volumetric kV or cone-beam CT imaging with shifts to bony anatomy at the level of the tumor as appropriate.

Orthogonal kV/MV, kV/kV projection, or CBCT imaging will be used to verify the location of the fiducials prior to delivery of the first treatment beam. A secondary shift based on the location of fiducials should be utilized, as indicated by the position of the fiducials. For free-breathing treatments, kV fluoroscopic images should be obtained to confirm the anticipated position of the fiducials (fiducial ITV) during the entire respiratory cycle.

Active monitoring of treatment delivery accuracy will be accomplished using kV and/or MV projection imaging, either immediately before or during all (or a subset of) treatment fields.

Patient-specific dosimetric quality assurance (QA) will be performed as per standard practice.

Synchrony respiratory tracking will be required for all patients treated with CyberKnife.

7.2.2 Compliance Criteria

7.2.2.1 Prescription Dose

Variation Acceptable: The dose to the prescription isodose surface differs from that in the protocol by between 6% and 10%.

Deviation Unacceptable: The dose to the prescription isodose surface differs from that in the protocol by more than 10%.

7.2.2.2 Dose Uniformity

Variation Acceptable: Minimum dose within the PTV is less than 97% of the prescribed dose, but does not fall below 93% of this dose or Maximum dose within the PTV is greater than 110% of the prescribed dose, but does not exceed 115% of this dose.

Deviation Unacceptable: Minimum dose within the PTV is less than 93% of the prescribed dose or Maximum dose is greater than 115% of the prescribed dose.

7.2.2.3 Volume

Deviation Unacceptable:

1. Incomplete contouring of the entire GTV or PTV;
2. Use of different margins than specified for the CTV and PTV;
3. Over-contouring of the GTV by >30cc (15cc if it results in inclusion of extra duodenum, small intestine or stomach);
4. Incorrect contouring of the duodenum, stomach or small intestine that results in >15 cc overlap of the PTV with the OAR.

7.2.2.4 Treatment Interruptions

Per Protocol: All treatments occur within 15 calendar days.

Variation Acceptable: All treatments occur within 16 to 21 calendar days.

Deviation Unacceptable: All treatments take 22 or more calendar days to complete.

Questions regarding the radiotherapy section of this protocol, including treatment interruptions, should be directed to:

Dr. Joseph Herman

The University of Texas MD Anderson Cancer Center

Phone: (713) 745-4193

jmherman@mdanderson.org

Dr. Michael Chuong

Miami Cancer Institute

Phone: (727) 421-3443

michaelchu@baptisthealth.net

7.2.3 Radiation Therapy Adverse Events

The criteria used for the grading of toxicities encountered in this study are Common Toxicity Criteria (CTC) version 4.0.

Very likely (80-90%)

Fatigue (which generally goes away after the radiation therapy is completed)

Skin irritation, redness, itchiness, discomfort

Temporary changes in blood work (decrease in blood counts, increase in liver enzymes), without symptoms

Less likely (30%)

Nausea, vomiting (during therapy) – more common if stomach or gastrointestinal track irradiated

Chest wall pain, rib fracture (< 10%)

Less likely, but serious (<20%)

Gastric, esophagus, small bowel or large bowel irritation/ulceration, bleeding, fistula, obstruction or changes in motility following therapy (may require medications or surgery) (< 10% permanent changes)

Radiation-induced liver disease (RILD) (<5%). Classic RILD is a clinical diagnosis of anicteric ascites, hepatomegaly and elevation of alkaline phosphatase relative to other transaminases that may occur 2 weeks to 3 months following radiation to the liver

Non-classic RILD includes elevation of liver enzymes and/or any decline in liver function within 12 weeks from start of therapy (~20%). RILD can lead to liver failure that could lead to death. There is an increased risk of liver toxicity in patients with large tumors and in patients with pre-existing liver disease.

Permanent thrombocytopenia (<1%); this may lead to bleeding

Kidney injury (<1%); this may lead to changes on imaging and more rarely the need for medication.

## 7.3 Surgery (Arm 1 and Arm 2)

Surgical resection of the primary tumor and regional lymph nodes in the absence of disease progression 4-8 weeks following chemotherapy and/or hypofractionated RT. **Sites are to inform the study chair of the scheduled date of surgery per** [**Section 7.3.3**](#Sec7p3p3)**.**

Surgical management of borderline resectable pancreatic tumors requires resection of the major mesenteric vasculature in the majority of cases, even following receipt of systemic chemotherapy and/or radiation. It is presumed that multidisciplinary teams enrolling patients on this study will have surgeons with significant experience in the management of these tumors. At a minimum, surgeons operating in the context of this study should be skilled and credentialed at their institution to perform vascular resection in addition to performing pancreatic surgery, and/or should team with a transplant or vascular surgery colleague who themselves are skilled and credentialed to perform vascular portions of the operations.

7.3.1 Surgical Quality Assurance

7.3.1.1 General considerations

Pancreaticoduodenectomy should occur within 4-8 weeks after the last dose of preoperative radiation (Arm 1) or chemotherapy (Arm 2). Staging laparoscopy may be performed at the time of planned laparotomy but is not required. Either standard or pylorus-preserving pancreaticoduodenectomy may be performed. Surgical drains and enteral tubes (e.g. gastrostomy and/or jejunostomy tubes) may be placed at the discretion of the operating surgeon.

The operation should be performed in accordance with techniques espoused in the first edition of Operative Standards for Cancer Surgery, published by the American College of Surgeons and the Alliance for Clinical Trials in Oncology.[37]

7.3.1.2 Specific considerations

Exploration of the peritoneal cavity should include evaluation for radiographically occult macroscopic peritoneal or hepatic metastases. Lymph node sampling or frozen section lymph node biopsy is not required or recommended as part of the intraoperative assessment for extra-pancreatic disease, and is at the discretion of the surgeon.

A standard lymphadenectomy should be performed routinely, to include lymph node stations 8a, 12a2, 12p2, 12b2, 12c, 13a, 13b, 14a, 14b, 17a, 17b, as depicted in [Appendix IV](#AppIV). Other lymph node stations dissected should be detailed if performed in the operative report.

The retroperitoneal dissection along the medial edge of the uncinate process and the right lateral border of the superior mesenteric artery is believed to be an important oncologic part of the operation. All soft tissue to the right of the superior mesenteric artery (SMA) should be removed. This requires exposure and dissection along the right lateral border of the SMA.

Vascular resection and/or reconstruction of the superior mesenteric vein, portal vein, SMA/portal vein confluence, or hepatic artery will be performed at the discretion of the operating surgeon. In general, vascular resection should be performed when necessary to achieve an R0 resection. The operating surgeon or a vascular/transplant surgeon consult can perform this reconstruction. The technical details of the operation should be delineated in the operative report.

7.3.1.3 Intraoperative Frozen Section Assessment of Surgical Margins

Frozen section evaluation of the pancreatic parenchymal and hepatic (or bile) duct margins should be performed. In the event of a positive frozen section margin at either of these loci, further resection in an effort to achieve microscopically negative margins should be performed if possible. However, the extent of additional parenchymal resection should be left to the discretion of the operating surgeon.

The superior mesenteric arterial (SMA) margin should be evaluated on permanent section only.

7.3.1.4 Specimen Orientation for Surgical Pathology

The surgeon should ensure that the specimen is oriented for the surgical pathologist. Any segment of resected vascular structure (e.g. superior mesenteric or portal vein) should be identified and marked. Relevant margins evaluated by intraoperative frozen section (i.e. the hepatic (bile) duct, and pancreatic parenchymal) should be identified. The SMA margin (the soft tissue immediately adjacent to the SMA) should be separately inked using the principles outlined in the 7th edition AJCC staging system for exocrine pancreatic cancer.  Note: The SMA margin cannot be identified accurately after the specimen has been fixed in formalin or after the specimen has been dissected for histopathologic analysis.

7.3.1.5 Aborting Surgery

The planned resection should be aborted if the operating surgeon identifies:

1. Metastatic disease in distant organs (e.g., liver). Presumed disease should be biopsied and confirmed as metastatic cancer on frozen section.
2. Localized cancer that is nonetheless, in the opinion of the operating surgeon, unsafe to resect. In such cases, the **specific reasons** for aborting the operation should be enumerated in the operative report.

7.3.1.6 Operative Note Dictation and Editing: Resection Classification

The attending surgeon should dictate the operative note. The operative report should contain:

- A section detailing the operative findings with respect to the extent of disease and the primary tumor anatomy.
- A statement as to whether or not the surgeon believes there is residual macroscopic tumor following completion of the resection.

The surgeon should integrate the operative findings with the microscopic surgical margins reported on the final pathology report in order to assign a resection classification prefix of R0, R1, or R2 (defined below). Whenever possible, this prefix should be added to the final operative note before finalizing the document. An example of the final procedure description for a patient who underwent macroscopically complete tumor removal with a positive SMA margin on permanent section final pathology is: “R1 pylorus-preserving pancreaticoduodenectomy.”  The definitions for the resection classification that should be utilized in operative notes include:

- R0 – macroscopically complete tumor removal with negative microscopic surgical margins (bile duct, pancreatic parenchyma, and SMA margins)
- R1 – macroscopically complete tumor removal with any positive microscopic surgical margin (bile duct, pancreatic parenchyma, or SMA margins)
- R2 – macroscopically incomplete tumor removal with known or suspected residual gross disease

7.3.1.7 Surgical Pathology

A local pathologist experienced in the diagnosis of pancreatic adenocarcinoma should carry out pathological examination of the resected pancreatic tumor specimen. Following local review, a tissue block will be sent for central re-review. See [Section 6.2.2](#Sec6p2p2).

Three primary margins (bile duct, pancreatic neck, and SMA) should be identified and inked by the surgeon and/or pathologist. Any segment of resected vascular structure (e.g. superior mesenteric or portal vein) should be identified and marked. The SMA margin (that tissue immediately adjacent to the SMA) should be separately inked according to the procedures and recommendations of the American Joint Commission on Cancer 7th edition staging system and the College of American Pathologists guidelines for reporting of resected exocrine pancreatic cancer (2012). The tumor should be thoroughly sampled (at least one section per 1 mm of greatest tumor dimension, taken perpendicular to the inked SMA margin). The distance between the closest tumor cell and the inked SMA margin (the “SMA margin distance”) should be reported.

7.3.1.8 Frozen Section Assessment of Margins

Section assessment of bile duct and pancreatic neck margins should be performed by the local pathologist in all cases as requested by the surgeon.

7.3.1.9 Permanent Section Assessments and Final Pathology Report

The pathology report should contain all of the elements outlined in the College of American Pathologists guidelines for reporting of resected exocrine pancreatic cancer (2012). In particular, there should be specific comment on:

- Histologic diagnosis with comment on the cell of origin (pancreatic vs. bile duct vs. ampulla)
- Degree of differentiation (well, moderate, poor)
- Total number of lymph nodes examined
- Number of positive nodes
- Final margins status for the bile duct, pancreatic parenchymal, and SMA margin
- Distance (in mm) from the tumor to the inked SMA margin
- Extent of tumor infiltration (if present) of the blood vessel wall for any resected major blood vessels including the maximum histologic depth of invasion (e.g., adventitia, media).

7.3.1.10 Central Re-Review of Resected Tumor Specimen

Representative slides should be sent for central re-review to standardize determination of both treatment effect score and tumor grade.

Treatment effect score will be determined using the following system:

- Treatment Effect Score I - 0% residual tumor cells in the specimen (pCR)
- Treatment Effect Score II - 1 to <5% residual tumor cells in the specimen
- Treatment Effect Score III - ≥5% residual tumor cells in the specimen

7.3.2 Definitions of Variations in Surgical Performance

Minor Variations

The following will be considered minor surgical variations:

- Failure to perform a frozen section of bile duct or pancreatic neck margins at the time of surgery.
- Documentation of an incomplete dissection of uncinate off of SMA.
- Documentation of a lymphadenectomy less extensive than that described above.

7.3.3 Surgical Quality Control

Sites scheduling patients for surgery should notify the study chair of their intent to operate at the time the operation is scheduled. This notification should consist of an email to the study chair with the Alliance patient ID number and date of surgery, along with contact information of the site treating surgeon and credited study PI on the OPEN form.

For all patients who undergo surgery during protocol treatment (including resection or not), the study chair and surgical co-chair will review the preoperative CT scan, the operative note, the surgical pathology report, and the adverse events associated with surgery (30 days postoperative) within 60 days of surgery. This requires that sites upload the required source documentation in a timely manner (see [Section 6.1.1](#Sec6p1p1)), and complete the AE CRF without delinquency.

Specific attention will be paid to deviations as listed above, as well as adverse events that occur in association with surgery. Operative reports and outcomes which, in the opinion of the study chair and surgical co-chair, raise potential patient safety concerns will be discussed and reviewed with the credited study PI immediately. Two operations which lead to such a discussion may lead to the site being restricted from enrolling future patients.

## 7.4 Imaging

7.4.1 Imaging Guidelines

CT scans are preferred over MRI and should be used if possible (unless the patient is allergic to contrast or has renal insufficiency, for example). Chest scans must be CT or chest X-ray. Abdominal baseline and restaging scans can include either a CT or MRI. The same method of scanning used at baseline must be used for all subsequent evaluations.

Sites are to use their optimal preoperative image acquisition protocol to optimize their ability to interpret the scans.

7.4.1.1 CT Scan

Dual phase imaging

- Slice thickness: No thicker than 3mm images (however, a set of axial images 2-3 mm for review of each phase is needed). Thinner images, if obtained, should also be submitted (in case reconstructions are needed).
- Injection rate: Faster is preferable, but whatever the injection rate, the duration of the injection should be at least 30 seconds (in order to image during the pancreatic parenchymal phase, and given an average cardiac circulation time of 13-16 seconds). Injection rates should be 3-5cc/second (unless poor IV access requires slower injection times).
- Pancreatic parenchymal phase: Imaging of the abdomen (whether the entire abdomen, or just of the pancreas) should finish approximately 45-50 seconds after contrast injection terminating below the level of the horizontal course of the duodenum.
- Portal venous phase: Imaging of the entire abdomen, to begin 55-70 seconds after the start of contrast injection, to cover the liver, and pancreas to the level of the iliac crest.
- For both phases, coronal and sagittal reconstructions (2-3mm thick, every 2-3mm) are encouraged, but optional.

**NOTE**: Separate/dedicated CTs of chest/upper abdomen must be obtained at all required time points.

7.4.1.2 Chest Scans

- Lung lesions should be excluded with CT of the chest or chest X-ray.

7.4.1.3 Pre-contrast Imaging

1. SSFSE/ HASTE T2 coronal scouts 5-8 mm thickness (with or without breath-hold, per institutional standards).
2. In-Out phase T1 breath-hold T1 GRE (4-6 mm thickness) to cover liver and pancreas.
3. Out- phase T1 GRE with or without fat sat (4-5 mm thickness) for pancreas only.
4. Respiratory Triggered FSE T2 weighted images (4-6 mm thickness for liver and pancreas).
5. 2D or 3D MRCP sequence for the pancreas as performed in your institution.

7.4.1.4 Contrast Scanning

- Sequence- 3D or 2D VIBE or SPGR/ LAVA T1 GRE (2-6 mm thickness, not >7 mm for 2D sequence) acquired preferably in the coronal or axial plane. First acquire a non-contrast sequence for confirming the coverage and quality.
- Inject Gd-chelate MR contrast volume with weight-based dosing according to the product label @ 2cc/sec.
- Dynamic scanning using a 3D or 2D VIBE or SPGR/ LAVA T1 GRE (2-6 mm thickness) is then performed in the same plane as the pre-contrast scanning at 20/70/180 seconds delay after contrast medium injection.
- Axial and coronal T1 out-phase T1FS GRE/VIBE through the liver and pancreas (same thickness as dynamic above). If needed use two sequences to cover the anatomy.

7.4.2 Central review of scans

Scans should be submitted to the ICL as outlined in [Section 6.4](#Sec6p4). There are 2 real-time scans that are reviewed by a central radiologist, which will be the reading utilized to make decisions on the protocol. If there are any uncertainties or the participating site disagrees with the first central review result, a second review by either another central reviewer or an adjudicator will be performed. This process may take additional 24-48 hours turnaround time, and such review result will be used as the final decision for the outcome determinations.

The 1^st^ real-time review is a determination of whether the patient has borderline resectable pancreatic cancer. If the review determines the patient is eligible, then registration procedures are followed per [Section 4.5](#Sec4p5).

The 2^nd^ real-time review takes place after preoperative therapy completion, and is a determination of whether the tumor is potentially resectable.

If the patient does NOT have borderline pancreatic cancer by central review, then they are not eligible for the study and are deemed a screen failure. The expected rate of screen failures is provided in [Section 13.2](#Sec13p2). If they are designated by the central reviewer to have borderline pancreatic cancer, then they meet eligibility criteria as per [Section 3.2.1](#Sec3p2p1).

The 2^nd^ real-time review takes place after preoperative therapy completion, and is a determination of whether the tumor is potentially resectable. If the 2^nd^ central review determines that the tumor meets criteria for resection, the IROC Ohio notifies the site via email, and the reviewer enters the data into Rave. The patient should then proceed to surgery as per [Section 7.3](#Sec7p3).

If the review determines the patient is NOT potentially resectable, then the patient should be removed from protocol therapy, and followed as per [Section 5.0](#Sec5p0), post-treatment follow-up. Protocol treatment will be discontinued and further treatment is at the discretion of the treating physician. See [Section 12.0](#Sec12p0) for further details.

# 8.0 Dose and Treatment Modifications

## 8.1 Ancillary Therapy, Concomitant Medications, and Supportive Care

8.1.1 Patients should not receive any other agent which would be considered treatment for the primary neoplasm or impact the primary endpoint.

8.1.2 Patients should receive full supportive care while on this study.

This includes blood product support, antibiotic treatment, and treatment of other newly diagnosed or concurrent medical conditions. All blood products and concomitant medications such as antidiarrheals, analgesics, and/or antiemetics received from the first day of study treatment administration until 30 days after the final dose will be recorded in the medical records.

8.1.3 Treatment with hormones or other chemotherapeutic agents may NOT be administered except for steroids given for adrenal failure; hormones administered for non-disease-related conditions (e.g., insulin for diabetes); and intermittent use of dexamethasone as an antiemetic in solid tumor protocols.

8.1.4 Antiemetics may be used at the discretion of the attending physician, with the exception of steroids above.

As this regimen has high emetogenic potential, it is suggested that all subjects on study should receive an aggressive prophylactic antiemetic regimen, consisting of a 5HT-3 antagonist, steroid, and NK1 antagonist.

8.1.5 Diarrhea management is per the discretion of the treating physician.

This regimen has a high incidence of severe diarrhea. For symptoms of diarrhea (and/or abdominal cramping) that occur at any time during a treatment cycle, it is suggested that patients should be instructed to take an anti-diarrheal, such as loperamide. It is recommended that the anti-diarrheal should be started at the earliest sign of: (1) a poorly formed or loose stool; (2) an increase in bowel movements by 1 to 2 episodes per day compared to baseline, or (3) an increase in stool volume or liquidity. Additional anti-diarrheal measures may be implemented at the discretion of the treating physician. Patients should also be instructed to increase fluid intake to help maintain fluid and electrolyte balance during episodes of diarrhea, and IV fluids should be considered for severe diarrhea, at the discretion of the treating physician.

8.1.6 Antibiotics

Prophylactic oral antibiotic therapy is not recommended to be given at the start of study treatment. However, antibiotics should be considered for patients who have: (a) endobiliary stent(s) with an ANC that falls below 500 mm3 or have fever; (b) diarrhea persisting for more than 48 hours despite treatment or fever with diarrhea.

8.1.7 Anticoagulants

Due to drug interactions, treatment dose warfarin for goal INR > 1.5 is not recommended. Other anticoagulants are recommended to be used for prophylaxis and treatment.

8.1.8 Palliative radiation therapy may not be administered while a subject is on the study.

8.1.9 Alliance Policy Concerning the Use of Growth Factors

Please note that during pre-surgical FOLFIRINOX treatment, WBC growth factors are required (see [Section 7.1.1](#Sec7p1p1)). During post-surgical FOLFOX, WBC growth factors are NOT required and may be used at the discretion of the physician.

Blood products and growth factors should be utilized as clinically warranted and following institutional policies and recommendations. The use of growth factors should follow published guidelines of the American Society of Clinical Oncology 2006 Update of Recommendations for the Use of White Blood Cell Growth Factors: An Evidence-Based, Clinical Practice Guideline. J Clin Oncol 24(19): 3187-3205, 2006.

The use of erythropoiesis–stimulating agents (ESAs) is allowed at the discretion of the treating investigator. If an ESA is used, it should be within the context of the most current guidelines.

The use of prophylactic white blood cell growth factor support is required for all study subjects, beginning with the first treatment cycle and administered as per established institutional guidelines.

Filgrastim (G-CSF) tbo-filgrastim, and sargramostim (GM-CSF)

1. Filgrastim (G-CSF)/pegfilgrastim, tbo-filgrastim and sargramostim (GM-CSF) treatment for patients on protocols that do not specify their use is discouraged.

2. Filgrastim/pegfilgrastim, tbo-filgrastim and sargramostim may not be used:

1. To avoid dose reductions, delays or to allow for dose escalations specified in the protocol.
2. For the treatment of febrile neutropenia the use of CSFs should not be routinely instituted as an adjunct to appropriate antibiotic therapy. However, the use of CSFs may be indicated in patients who have prognostic factors that are predictive of clinical deterioration such as pneumonia, hypotension, multi-organ dysfunction (sepsis syndrome) or fungal infection, as per the ASCO guidelines. Investigators should therefore use their own discretion in using the CSFs in this setting. The use of CSF (filgrastim/pegfilgrastim or sargramostim) must be documented and reported. (e.g. on CRFs per protocol requirements)
3. If filgrastim/pegfilgrastim, tbo-filgrastim or sargramostim are used, they must be obtained from commercial sources.

8.1.10 CYP3A4 Inhibitors

Chronic concomitant treatment with strong inhibitors of CYP3A4 is not allowed on this trial. The following drugs are EXAMPLES of strong inhibitors of CYP3A4 and are not allowed during treatment with irinotecan.

- Indinavir
- Clarithromycin
- Ketoconazole

Because lists of these agents are constantly changing, please consult and review any drugs for their potential to inhibit CYP3A4. Examples of resources that may be utilized include the product information for the individual concomitant drug in question, medical reference texts such as the FDA website, or your local institution’s pharmacist.

An information handout and wallet-size card providing information for patients and their caregivers regarding potential drug interactions have been made available in [Appendix III](#AppIII).

8.1.11 CYP3A4 Inducers

Chronic concomitant treatment with strong inducers of CYP3A4 is not allowed on this trial. The following drugs are EXAMPLES of strong inducers of CYP3A4 and are not allowed during treatment with irinotecan.

- Rifampin
- Carbamazepine

Because lists of these agents are constantly changing, please consult and review any drugs for their potential to induce CYP3A4. Examples of resources that may be utilized include the product information for the individual concomitant drug in question, medical reference texts such as the FDA website, or your local institution’s pharmacist.

An information handout and wallet-size card providing information for patients and their caregivers regarding potential drug interactions have been made available in [Appendix III](#AppIII).

8.1.12 Biliary obstruction:

Biliary decompression should be performed if clinically warranted. Biliary decompression should ideally be performed during the pre-registration phase and should be accomplished endoscopically, preferably with a short metallic stent.

8.1.13 Hypersensitivity/infusion reactions

Treat hypersensitivity and infusion reactions as per institutional standards.

8.1.14 Cholinergic reaction:

Lacrimation, rhinorrhea, miosis, diaphoresis, hot flashes, flushing, abdominal cramping, diarrhea, or other symptoms of early cholinergic syndrome may occur during or shortly after receiving irinotecan. Atropine, 0.25-1mg IV or SC may be used to treat these symptoms. In patients with troublesome or recurrent symptoms, prophylactic administration of atropine shortly before irinotecan therapy may be considered. Additional antidiarrheal measures may be used at the discretion of the treating physician. Combination anticholinergic medications containing barbiturates or other agents (e.g., DonnatalR) should not be used because these may affect irinotecan metabolism. Anticholinergics should be used with caution in patients with potential contraindications (e.g., obstructive uropathy, glaucoma, tachycardia, etc.).

8.1.15 Neurologic toxicity:

Supportive care is allowed at the discretion of the treating physician. For pharyngo-laryngeal dysesthesia, it is recommended to increase the duration of oxaliplatin infusion to 6 hours for subsequent cycles.

8.1.16 Extravasation

Extravasation of oxaliplatin has been associated with necrosis. Extravasation should be treated according to institutional guidelines.

## 8.2 Dose Modifications

CTEP-AERS reporting may be required for some adverse events (See [Section 9.4](#Sec9p4)).

**NOTE:** PRO-CTCAE data should not be used for determining attribution, dose modifications, or reporting serious adverse events.

8.2.1 Dose Levels for neoadjuvant mFOLFIRINOX and adjuvant FOLFOX

General Guidelines for mFOLFIRINOX and FOLFOX:

If multiple adverse events are seen, administer dose based on greatest reduction required for any single adverse event observed. Reductions or holds for new cycles of treatment are based on treatment given in the preceding cycle and adverse events observed since the prior dose.

Dose modifications of 5-FU, irinotecan, and oxaliplatin may occur independently of each other, based on the pattern of toxicity. Patients unable to tolerate one or more drugs due to toxicity will remain on treatment with the other drugs.

During FOLFOX, follow the same instructions for modifications, excluding instructions for irinotecan.

If 5-FU is omitted, leucovorin should also be omitted.

Once the dose of any drug has been reduced, the dose cannot be re-escalated.

If any drug is held for > 4 weeks due to treatment-related toxicity, then that drug should be permanently discontinued.

There are no further dose reductions beyond those listed in the tables below for each drug. If further dose reduction is required, that drug should be discontinued.

mFOLFIRINOX Dose Levels:

| **Dose Level** | **5-FU infusion (mg/m^2^)** | **Leucovorin**  **(mg/m^2^)** | **Irinotecan (mg/m^2^)** | **Oxaliplatin (mg/m^2^)** |
| --- | --- | --- | --- | --- |
| 0* | 2400 | 400 | 180 | 85 |
| -1 | 1920 | 400 | 150 | 65 |
| -2 | 1600 | 400 | 120 | 50 |
| -3 | 1360 | 400 | 100 | 40 |

*Dose level 0 refers to the starting dose.

FOLFOX Dose Levels:

| **Dose Level** | **5-FU bolus**  **(mg/m^2^)** | **5-FU infusion (mg/m^2^)** | **Leucovorin**  **(mg/m^2^)** | **Oxaliplatin (mg/m^2^)** |
| --- | --- | --- | --- | --- |
| 0* | 400 | 2400 | 400 | 85 |
| -1 | 320 | 1920 | 400 | 65 |
| -2 | 250 | 1600 | 400 | 50 |
| -3 | 200 | 1360 | 400 | 40 |

*Dose level 0 refers to the starting dose.

8.2.2 Dose Modifications for mFOLFIRINOX and FOLFOX

8.2.2.1 Hematologic Toxicity

For **grade 2 neutrophil count decreased on day 1**, delay 5FU, oxaliplatin, and irinotecan until grade ≤ 1, then resume oxaliplatin, irinotecan, and 5FU at same dose

For **grade 3 neutrophil count decreased on day 1**, delay 5FU, oxaliplatin, and irinotecan until grade ≤ 1, then resume oxaliplatin and irinotecan at 1 dose level decreased, and 5FU at same dose

For **grade 4 neutrophil count decreased or febrile neutropenia**, delay 5FU, oxaliplatin, and irinotecan until grade ≤ 1, then resume 5FU, oxaliplatin, and irinotecan with one dose level decreased

For **grade 2 platelet count decreased**, delay 5FU, oxaliplatin, and irinotecan until grade ≤ 1, then resume oxaliplatin and irinotecan at one dose level decreased, and 5FU at same dose

For **grade 3 or 4 platelet count decreased**, delay 5FU, oxaliplatin, and irinotecan until grade ≤ 1, then resume 5FU, oxaliplatin, and irinotecan with one dose level decreased

8.2.2.2 Gastrointestinal toxicity with optimal medical management

For **grade 2 diarrhea**, delay 5FU, oxaliplatin, and irinotecan until recovery to grade ≤ 1 or baseline, then resume 5FU, oxaliplatin, and irinotecan at the same dose level.

For **grade 3 diarrhea**, delay 5FU, oxaliplatin, and irinotecan until recovery to grade ≤ 1 or baseline, then resume 5-FU, and oxaliplatin at the same dose level and irinotecan at the next lower dose level.

For **grade 4 diarrhea** , delay 5FU, oxaliplatin, and irinotecan until recovery to grade ≤ 1 or baseline then resume 5FU, oxaliplatin, and irinotecan at the next lower dose level.

For **grade 3 or 4 Nausea/Vomiting**, delay 5FU, oxaliplatin, and irinotecan until recovery to grade ≤ 1, then resume 5FU, oxaliplatin, and irinotecan at the next lower dose level.

8.2.2.3 Mucositis

**For grade 3 mucositis**, delay 5FU, oxaliplatin, and irinotecan until recovery to grade ≤ 1, then resume irinotecan and oxaliplatin at the same dose level and 5-FU at the next lower dose level.

For **grade 4 mucositis**: Delay 5FU, oxaliplatin, and irinotecan until recovery to grade ≤1, then resume 5FU, oxaliplatin, and irinotecan at the next lower dose level.

8.2.2.4 Neuropathy (sensory or motor)

For **grade 2 neurotoxicity**, decrease oxaliplatin by one dose level.

For **grade 3 or 4 neurotoxicity**, discontinue oxaliplatin.

8.2.2.5 Hepatobiliary Toxicity

For **grade 2 bilirubin increased,** omit irinotecan until grade ≤ 1, then resume at same dose.

For **grade 3 or 4 bilirubin increased,** delay.5FU, oxaliplatin, and irinotecan until recovery to grade ≤1, then resume 5FU, oxaliplatin, and irinotecan at the next lower dose level.

**For grade 3 or 4 ALT/AST increased,** discontinue 5FU, oxaliplatin, and irinotecan

8.2.2.6 Renal Insufficiency

For **grade 3 or 4 creatinine increased** and NOT attributed to study treatment, delay 5-FU, oxaliplatin, and irinotecan until grade ≤ 1 then restart irinotecan at the same doses.

8.2.2.7 Other non-hematologic toxicities

For all other **grade 3 non-hematologic toxicities considered at least possibly related to treatment**, omit the responsible drug(s) until toxicity improves to ≤ grade 1, then resume the responsible drug(s) at the next lower dose level.

For **grade 4 non-hematologic toxicities considered at least possibly related to treatment**, discontinue the responsible drug(s).

8.2.3 Dose Modifications for Obese Patients

There is no clearly documented adverse impact of treatment of obese patients when dosing is performed according to actual body weight. Therefore, all dosing is to be determined solely by actual weight without any modification unless explicitly described in the protocol. This will eliminate the risk of calculation error and the possible introduction of variability in dose administration. Failure to use actual body weight in the calculation of drug dosages will be considered a major protocol deviation. Physicians who are uncomfortable with calculating doses based on actual body weight should recognize that doing otherwise would be a protocol violation. Physicians may consult the published guidelines of the American Society of Clinical Oncology Appropriate Chemotherapy Dosing for Obese Adult Patients with Cancer: American Society of Clinical Oncology Clinical Practice Guideline. J Clin Oncol 30(13): 1553-1561, 2012.

8.2.4 Dose Modifications for HIGRT and SBRT

Radiation treatment should be delayed one day if patients are unable to tolerate radiation treatment because of nausea, vomiting, or other illness. Also, if stomach or bowel gas/filling makes treatment set-up unreliable, treatment should be delayed. Every effort should be made to provide supportive management (nausea medications) in order to complete radiation treatment within the two week time frame.

# 9.0 Adverse Events

The prompt reporting of adverse events is the responsibility of each investigator engaged in clinical research, as required by Federal Regulations. Adverse events must be described and graded using the terminology and grading categories defined in the NCI’s Common Terminology Criteria for Adverse Events (CTCAE), Version 4.0. The CTCAE is available at ctep.cancer.gov/protocolDevelopment/electronic_applications/ctc.htm. Attribution to protocol treatment for each adverse event must be determined by the investigator and reported on the required forms. Please refer the NCI Guidelines: Adverse Event Reporting Requirements for further details on AE reporting procedures. To complement CTCAE reporting, patients will self-report their side effects using the PRO-CTCAE. The specific PRO-CTCAE items for this protocol can be found in [Appendix I](#AppI). They can also be found at: <http://healthcaredelivery.cancer.gov/pro-ctcae/instrument.html>.

**NOTE:** PRO-CTCAE data should not be used for determining attribution, dose modifications, or reporting of serious adverse events.

## 9.1 Routine Adverse Event Reporting

Adverse event data collection and reporting, which are required as part of every clinical trial are done to ensure the safety of patients enrolled in the studies as well as those who will enroll in future studies using similar agents. Adverse events are reported in a routine manner at scheduled times according to the study calendar in [Section 5.0](#Sec5p0). For this trial, the form, “Adverse Events: Solicited,” is used for routine AE reporting in Rave.

PRO-CTCAE paper booklets ordered from the CTSU website are to be administered by a nurse/CRA and completed by the patient at scheduled times according to the study calendar in [Section 5.0](#Sec5p0), and then entered into Rave.

**Solicited Adverse Events:** The following adverse events are considered "expected" and their presence/absence should be solicited, and severity graded, at baseline and for each cycle of treatment by CTCAE, PRO-CTCAE, or both.

| CTCAE v4.0 Term | PRO-CTCAE v1.0 Term | CTCAE v4.0 System Organ Class (SOC) |
| --- | --- | --- |
| Blood bilirubin increased |  | Investigations |
| Hypoalbuminemia |  | Investigations |
| Wound infection |  | Infections and infestations |
| Bile duct stenosis |  | Hepatobiliary disorders |
| Diarrhea | Loose or watery stools (diarrhea) | Gastrointestinal disorders |
| Nausea | Nausea | Gastrointestinal disorders |
| Fatigue | Fatigue, tiredness, or lack of energy | General disorders |
| Weight loss |  | Investigations |
| Peripheral sensory neuropathy | Numbness or tingling in your hands or feet | Nervous system disorders |
|  | Dry mouth | Gastrointestinal disorders |
|  | Problems with tasting food or drink | Nervous system disorder |
|  | Decreased appetite | Metabolism and nutrition disorders |
|  | Vomiting | Gastrointestinal disorders |
|  | Heartburn | Gastrointestinal disorders |
|  | Bloating of the abdomen (belly) | Gastrointestinal disorders |
|  | Pain in the abdomen (belly area) | Gastrointestinal disorders |
|  | Itchy skin | Skin and subcutaneous tissue disorders |
|  | Pain | General disorders |
|  | Anxiety | Psychiatric disorders |
|  | Sad or unhappy feelings | Psychiatric disorders |

## 9.2 Surgical Adverse Events

Specific toxicities related to pancreatic cancer surgery must be evaluated to best determine the adverse events of the protocol. The following events are collected at a single time point, which is 90 days after surgery:

| **Event** | **Data to be entered** |
| --- | --- |
| Pancreatic fistula requiring drainage within 90 days of surgery | Yes/No |
| Abdominal abscess requiring drainage within 90 days of surgery | Yes/No |
| Patients with a hospitalization within 90 days of surgery: Length of hospital stay | Number of days |
| Reoperation for any reason within 90 days of surgery | Yes/No |

## 9.3 CTCAE Routine Reporting Requirements

In addition to the solicited adverse events listed in [Section 9.1](#Sec9p1), the following table outlines the combinations of time points, grades and attributions of AEs that require routine reporting to the Alliance Statistics and Data Center. Questions about routine reporting should be directed to the Data Manager.

**NOTE:** PRO-CTCAE data should not be used for determining attribution, dose modifications, or reporting serious adverse events.

*Combinations of CTCAE Grade & Attribution Required for Routine AE Data Submission on Case Report Forms (CRFs)

| **Attribution** | **Grade 1** | **Grade 2** | **Grade 3** | **Grade 4** | **Grade 5** |
| --- | --- | --- | --- | --- | --- |
| Unrelated |  |  | a | a | a |
| Unlikely |  |  | a | a | a |
| Possible |  | a | a, b | a, b | a, b |
| Probable |  | a | a, b | a, b | a, b |
| Definite |  | a | a, b | a, b | a, b |

1. Adverse Events: Other CRF - Applies to AEs occurring between registration and within 30 days of the patient’s last treatment date, or as part of the Clinical Follow-Up Phase.
2. Adverse Events: Late CRF - Applies to AEs occurring greater than 30 days after the patient’s last treatment date.

## 9.4 Expedited Adverse Event Reporting (CTEP-AERS)

Investigators are required by Federal Regulations to report serious adverse events as defined in the table below. Alliance investigators are required to notify the Investigational Drug Branch (IDB), the Alliance Central Protocol Operations Program, the Study Chair, and their Institutional Review Board if a patient has a reportable serious adverse event. The descriptions and grading scales found in the NCI Common Terminology Criteria for Adverse Events (CTCAE) version 4 will be utilized for AE reporting.  The CTCAE is identified and located on the CTEP website at: ctep.cancer.gov/protocolDevelopment/electronic_applications/ctc.htm. All appropriate treatment areas should have access to a copy of the CTCAE. All reactions determined to be “reportable” in an expedited manner must be reported using the Cancer Therapy Evaluation Program Adverse Event Reporting System (CTEP-AERS).

For further information on the NCI requirements for SAE reporting, please refer to the ‘NCI Guidelines for Investigators: Adverse Event Reporting Requirements’ document published by the NCI.

**NOTE:** PRO-CTCAE data should not be used for determining attribution, dose modifications, or reporting serious adverse events.

Note: All deaths on study require both routine and expedited reporting regardless of causality. Attribution to treatment or other cause should be provided.

9.4.1 Expedited Reporting Requirements for Adverse Events that Occur in a Non-IND/IDE trial ≤ 30 Days of the Last Day of Treatment^1^

| **FDA REPORTING REQUIREMENTS FOR SERIOUS ADVERSE EVENTS (21 CFR Part 312)**  **NOTE:** Investigators **MUST** immediately report to the sponsor (NCI) **ANY** Serious Adverse Events, whether or not they are considered related to the investigational agent(s)/intervention (21 CFR 312.64)  An adverse event is considered serious if it results in **ANY** of the following outcomes:   1. Death 2. A life-threatening adverse event 3. An adverse event that results in inpatient hospitalization or prolongation of existing hospitalization for ≥ 24 hours 4. A persistent or significant incapacity or substantial disruption of the ability to conduct normal life functions 5. A congenital anomaly/birth defect. 6. Important Medical Events (IME) that may not result in death, be life threatening, or require hospitalization may be considered serious when, based upon medical judgment, they may jeopardize the patient or subject and may require medical or surgical intervention to prevent one of the outcomes listed in this definition. (FDA, 21 CFR 312.32; ICH E2A and ICH E6). | | | | | |
| --- | --- | --- | --- | --- | --- |
| **ALL SERIOUS** adverse events that meet the above criteria **MUST** be immediately reported to the NCI via CTEP-AERS within the timeframes detailed in the table below. | | | | | |
| **Hospitalization** | - **Grade 1 Timeframes** | - **Grade 2 Timeframes** | - **Grade 3 Timeframes** | | - **Grade 4 & 5 Timeframes** |
| Resulting in Hospitalization ≥ 24 hrs | 10 Calendar Days | | | | 24-Hour;  5 Calendar Days |
| Not resulting in  Hospitalization ≥ 24 hrs | Not required | | | 10 Calendar Days |  |
| **NOTE^:^** Protocol specific exceptions to expedited reporting of serious adverse events are found in the Specific Protocol Exceptions to Expedited Reporting (SPEER) portion of the CAEPR  **Expedited AE reporting timelines are defined as:**   - “24-Hour; 5 Calendar Days” - The AE must initially be reported via CTEP-AERS ≤ 24 hours of learning of the AE, followed by a complete expedited report ≤ 5 calendar days of the initial 24-hour report. - “10 Calendar Days” - A complete expedited report on the AE must be submitted ≤ 10 calendar days of learning of the AE. | | | | | |
| ^1^ Serious adverse events that occur more than 30 days after the last administration of investigational agent/intervention and have an attribution of possible, probable, or definite require reporting as follows:  **Expedited 24-hour notification followed by complete report ≤ 5 calendar days for:**   - All Grade 4, and Grade 5 AEs   **Expedited 10 calendar day reports for:**   - - - - Grade 2 adverse events resulting in hospitalization or prolongation of hospitalization - Grade 3 adverse events | | | | | |

• Expedited AE reporting timelines defined:

⮚ “24 hours; 5 calendar days” – The investigator must initially report the AE via CTEP-AERS ≤ 24 hours of learning of the event followed by a complete CTEP-AERS report ≤ 5 calendar days of the initial 24-hour report.

⮚ “10 calendar days” - A complete CTEP-AERS report on the AE must be submitted ≤ 10 calendar days of the investigator learning of the event.

• Any medical event equivalent to CTCAE grade 3, 4, or 5 that precipitates hospitalization (or prolongation of existing hospitalization) must be reported regardless of attribution and designation as expected or unexpected with the exception of any events identified as protocol-specific expedited adverse event reporting exclusions (see below).

• Any event that results in persistent or significant disabilities/incapacities, congenital anomalies, or birth defects must be reported via CTEP-AERS if the event occurs following treatment with an agent under a CTEP IND.

• Use the NCI protocol number and the protocol-specific patient ID provided during trial registration on all reports.

Additional Instructions or Exclusions to CTEP-AERS Expedited Reporting Requirements

• All adverse events reported via CTEP-AERS (i.e., serious adverse events) should also be forwarded to your local IRB.

• Grade 1-3 nausea or vomiting and hospitalization resulting from such do not require AERS reporting, but should be reported via routine AE reporting.

• Grade 3 or 4 nausea or vomiting does not require AERS reporting, but should be reported via routine AE reporting

• Grade 1-3 diarrhea and hospitalization resulting from such does not require AERS reporting, but should be reported via routine AE reporting

• Grade 3 or 4 diarrhea does not require AERS reporting, but should be reported via routine AE reporting

• Grade 1-3 mucositis and hospitalization resulting from such does not require AERS reporting, but should be reported via routine AE reporting

• Grade 3 or 4 mucositis does not require AERS reporting, but should be reported via routine AE reporting

• Grade 1-3 neuropathy and hospitalization resulting from such does not require AERS reporting, but should be reported via routine AE reporting.

• Grade 3 neuropathy does not require AERS reporting, but should be reported via routine AE reporting

• Grade 1-3 hand foot syndrome and hospitalization resulting from such does not require AERS reporting, but should be reported via routine AE reporting

• Grade 3 hand foot syndrome does not require AERS reporting, but should be reported via routine AE reporting

• Grade 1-4 hypersensitivity reaction and hospitalization resulting from such does not require AERS reporting, but should be reported via routine AE reporting.

• Grade 3 or 4 hypersensitivity reaction does not require AERS reporting, but should be reported via routine AE reporting

• Grade 1-3 dehydration and hospitalization resulting from such does not require AERS reporting, but should be reported via routine AE reporting

• Grade 3 dehydration does not require AERS reporting, but should be reported via routine AE reporting

• Grade 1-3 fatigue and hospitalization resulting from such does not require AERS reporting, but should be reported via routine AE reporting.

• Grade 3 or 4 fatigue does not require AERS reporting, but should be reported via routine AE reporting

• Grade 1-4 hematosuppression (leukopenia, neutropenia, lymphopenia, anemia, and thrombocytopenia) with hospitalization resulting from such do not require AERs reporting, but should be reported via routine AE reporting

• Grade 3 or 4 hematosuppression (leukopenia, neutropenia, lymphopenia, anemia, and thrombocytopenia) does not require AERs reporting, but should be reported via routine AE reporting

• Deaths that occur within 90 days of surgery required CTEP-AERS reporting regardless of attribution, including in the case of progressive disease.

• All new malignancies must be reported via CTEP-AERS whether or not they are thought to be related to either previous or current treatment. All new malignancies should be reported, i.e. solid tumors (including non-melanoma skin malignancies), hematologic malignancies, myelodysplastic syndrome/acute myelogenous leukemia, and in situ tumors. In CTCAE version 4.0, the new malignancies (both second and secondary) may be reported as one of the following: (1) Leukemia secondary to oncology chemotherapy, (2) Myelodysplastic syndrome, (3) Treatment-related secondary malignancy, or (4) Neoplasms benign, malignant and unspecified-other. Whenever possible, the CTEP-AERS reports for new malignancies should include tumor pathology, history or prior tumors, prior treatment/current treatment including duration, any associated risk factors or evidence regarding how long the new malignancy may have been present, when and how the new malignancy was detected, molecular characterization or cytogenetics of the original tumor (if available) and of any new tumor, and new malignancy treatment and outcome, if available.

CTEP-AERS reports should be submitted electronically.

When submitting CTEP-AERS reports for “Pregnancy”, “Pregnancy loss”, or “Neonatal loss”, the Pregnancy Information Form should be completed and submitted, along with any additional medical information (form is available on the CTEP website at http://ctep.cancer.gov/). The potential risk of exposure of the fetus to the investigational agent(s) or chemotherapy agent(s) should be documented in the “Description of Event” section of the CTEP-AERS report.

## 9.5 CDUS Reporting

The following is for Alliance Statistician and Data Center use only:

This study will be monitored by the Clinical Data Update System (CDUS) Version 3.0. Cumulative protocol- and patient-specific CDUS data will be submitted electronically to CTEP on a quarterly basis by FTP burst of data. Reports are due January 31, April 30, July 31, and October 31. Instructions for submitting data using the CDUS can be found on the CTEP Web site (<http://ctep.cancer.gov/reporting/cdus.html>).

This study has been assigned CDUS-Abbreviated reporting.

**Note**: If your study has been assigned to CDUS-Complete reporting, **all** adverse events (both routine and expedited) that have occurred on the study and meet the mandatory CDUS reporting guidelines must be reported via the monitoring method identified above. If your study has been assigned to CDUS-Abbreviated reporting, no adverse event reporting (routine or expedited) is required to be reported via CDUS, but expedited adverse events are still required to be submitted via CTEP-AERS.

# 10.0 Drug Information

## 10.1 General Considerations

It is not necessary to change the doses of chemotherapy drugs due to changes in weight unless the calculated dose changes by ≥10%.

Study agents may be administered at a non-registering institution. If the NCTN Group credited for enrollment is a non-Alliance Group, then other requirements from the credited Group may apply.

## 10.2 Oxaliplatin (Eloxatin) – NSC #266046

For more information, please refer to the drug package insert.

Procurement

Commercial supplies. Pharmacies or clinics shall obtain supplies from normal commercial supply chain or wholesaler.

Formulation

Commercially available as:

Solution for Injection: 50 mg/10 mL (10 mL); 100 mg/20 mL (20 mL); 200 mg/40 mL (40 mL)

Lyophilized Powder for Injection: 50 mg and 100 mg

Preparation, Storage and Stability

Refer to package insert for complete preparation and dispensing instructions. Store intact vials in original outer carton at room temperature and; do not freeze. According to the manufacturer, solutions diluted for infusion are stable up to 6 hours at room temperature or up to 24 hours under refrigeration. Oxaliplatin solution diluted with D5W to a final concentration of 0.7 mg/mL (polyolefin container) has been shown to retain >90% of its original concentration for up to 30 days when stored at room temperature or refrigerated; artificial light did not affect the concentration (Andre, 2007). As this study did not examine sterility, refrigeration would be preferred to limit microbial growth. Do not prepare using a chloride-containing solution (e.g., NaCl). Dilution with D5W (250 or 500 mL) is required prior to administration. Infusion solutions do not require protection from light.

Administration

Refer to the treatment section for specific administration instructions. Administer as IV infusion over 2-6 hours. Flush infusion line with D5W prior to administration of any concomitant medication. Patients should receive an antiemetic premedication regimen. Cold temperature may exacerbate acute neuropathy. Avoid mucositis prophylaxis with ice chips during oxaliplatin infusion.

Drug Interactions

Increased Effect/Toxicity: Nephrotoxic agents may increase oxaliplatin toxicity.

When administered as sequential infusions, observational studies indicate a potential for increased toxicity when platinum derivatives (carboplatin, cisplatin, oxaliplatin) are administered before taxane derivatives (docetaxel, paclitaxel).

Decreased Effect: Oxaliplatin may decrease plasma levels of digoxin

Pharmacokinetics

Distribution: V_d_: 440 L

Protein binding: >90% primarily albumin and gamma globulin (irreversible binding to platinum)

Metabolism: Nonenzymatic (rapid and extensive), forms active and inactive derivatives

Phase: 16.8 hours

Excretion: Primarily urine (~54%); feces (~2%)

Adverse Events

Consult the package insert for the most current and complete information. Percentages reported with monotherapy.

Common known potential toxicities, > 10%:

Central nervous system: Fatigue, fever, pain, headache, insomnia

Gastrointestinal: Nausea, diarrhea, vomiting, abdominal pain, constipation, anorexia, stomatitis

Hematologic: Anemia, thrombocytopenia, leukopenia

Hepatic: Liver enzymes increased

Neuromuscular & skeletal: Back pain, peripheral neuropathy (may be dose limiting). The most commonly observed oxaliplatin toxicity is acute and cumulative neurotoxicity, observed in patients treated at doses above 100 mg/m^2^/cycle. This neurotoxicity has included paresthesias and dysesthesias of the hands, feet, and perioral region as well as unusual laryngopharyngeal dysesthesias characterized by a loss of sensation of breathing without any objective evidence of respiratory distress (hypoxia, laryngospasm, or bronchospasm). OXAL neurotoxicity appears to be exacerbated by exposure to cold. Patients on this study will be counseled to avoid cold drinks and exposure to cold water or air. Should a patient develop laryngopharyngeal dysesthesia, their oxygen saturation should be evaluated via a pulse oximeter; if normal, an anxiolytic agent should be given and the patient observed in the clinic until the episode has resolved. Because this syndrome may be associated with the rapidity of OXAL infusion, subsequent doses of OXAL should be administered as a 6-hour infusion (instead of the normal 2‑hour infusion).

Acute and cumulative neurotoxicities are dose limiting for OXAL. The acute neurotoxicity is characterized by paresthesias and dysesthesias that may be triggered or exacerbated by exposure to cold. These symptoms occur within hours of exposure and are usually reversible over the following hours or days. Cumulative doses of OXAL above 680 mg/m^2^ may produce functional impairment characterized by difficulty performing activities requiring fine sensory-motor coordination; impairment is caused by sensory rather than motor changes.

The likelihood of experiencing neurotoxicity is directly related to the total cumulative dose of OXAL administered. The relative risk of developing neurotoxicity was 10%, 50%, and 75% in patients who received total cumulative OXAL doses of 780 mg/m^2^, 1,170 mg/m^2^, and 1,560 mg/m^2^, respectively. Both acute and cumulative neurotoxicities due to OXAL have lessened in 82% of patients within 4 to 6 months, and have completely disappeared by 6 to 8 months in 41% of patients. In addition, the likelihood that neurologic symptoms will regress has been shown to correlate inversely with cumulative dose.

Respiratory: Dyspnea, cough

Less common known potential toxicities, 1% - 10%:

Cardiovascular: Edema, chest pain, peripheral edema, flushing, thromboembolism

Central nervous system: Dizziness

Dermatologic: Rash, alopecia, hand-foot syndrome

Endocrine & metabolic: Dehydration, hypokalemia

Gastrointestinal: Dyspepsia, taste perversion, flatulence, mucositis, gastroesophageal reflux, dysphagia

Genitourinary: Dysuria

Hematologic: Neutropenia

Local: Injection site reaction

Neuromuscular & skeletal: Rigors, arthralgia

Ocular: Abnormal lacrimation

Renal: Serum creatinine increased

Respiratory: URI, rhinitis, epistaxis, pharyngitis, pharyngolaryngeal dysesthesia

Miscellaneous: Allergic reactions, hypersensitivity (includes urticaria, pruritus, facial flushing, shortness of breath, bronchospasm, diaphoresis, hypotension, syncope, hiccup

Rare known potential toxicities, <1% (Limited to important or life-threatening):

Gastrointestinal: Life threatening enteric sepsis secondary to neutropenia and diarrhea.

Hepatic: Veno-occlusive disease of the liver is a rare serious adverse event that has occurred in association with administration of oxaliplatin and fluorouracil.

Otic: Clinical ototoxicity occurs in less than 1% of patients following oxaliplatin administration, and sever ototoxicity has not been reported

Nursing Guidelines

GI toxicity similar to cisplatin occurs with doses above 30 mg/m^2^. It can be almost constant and frequently severe, but not always dose-limiting. Monitor for nausea and vomiting and treat accordingly.

Dose-limiting side effect can be paresthesias of hands, fingers, toes, pharynx, and occasionally cramps which develops with a dose-related frequency (>90 mg/m^2^). Duration of symptoms tend to be brief (less than a week) with the first course, but longer with subsequent courses. Phase I patients have reported exacerbation of paresthesias by touching cold surfaces or exposure to cold. Advise patient of these possibilities and instruct patient to report these symptoms to the health care team. Also advise patient to refrain from operating dangerous machinery that requires fine sensory-motor coordination, if symptoms appear.

These sensory neuropathies developed after subsequent courses with increasing intensity (Grade 3 toxicity after the fourth course) and with increasing duration. In 63% of the patients tested in phase I at high doses (135-200 mg/m^2^), neuropathies became long-term with slow reversal over several months. Disabling walking and handwriting difficulties, as well as mouth and throat dysesthesias and laryngospasms were seen. Instruct patient to report any swallowing difficulties or gait changes.

OXAL is incompatible with NS. Flush lines with D5W prior to and following OXAL infusion.

Low back pain is a common side effect, perhaps a form of hypersensitivity reaction. Instruct patient in good body mechanics, advise light massage, heat, etc.

Laryngopharyngeal dysesthesia (LPD) occurs in about 15% of patients and is acute, sporadic, and self-limited. It usually occurs within hours of infusion, is induced or exacerbated by exposure to cold, and presents with dyspnea and dysphagia. The incidence and severity appear to be reduced by prolonging infusion time. Instruct patient to avoid ice and cold drinks the day of infusion. If ≥ Grade 2 laryngopharyngeal dysesthesia occurs during the administration of OXAL, do the following:

- Stop OXAL infusion
- Administer benzodiazepine and give patient reassurance
- Test oxygen saturation via a pulse oximeter
- At the discretion of the investigator, the infusion can be restarted at 1/3 the original rate of infusion.
- Rapid resolution is typical within minutes to a few hours. Can recur with retreatment.

| **Comparison of the Symptoms and Treatment of Laryngopharyngeal Dysesthesias and Platinum Hypersensitivity Reactions** | | |
| --- | --- | --- |
| **Clinical Symptoms** | **Laryngopharyngeal**  **Dysesthesias** | **Platinum**  **Hypersensitivity** |
| Dyspnea | present | present |
| Bronchospasm | absent | present |
| Laryngospasm | absent | present |
| Anxiety | present | present |
| O_2_ saturation | normal | decreased |
| difficulty swallowing | present  (loss of sensation) | absent |
| Pruritus | absent | present |
| urticaria/rash | absent | present |
| cold-induced symptoms | yes | no |
| BP | normal or increased | normal or decreased |
| **Treatment** | anxiolytics, observation in a controlled clinical setting until symptoms abate or at the physicians’ discretion | oxygen, steroids, epinephrine, bronchodilators; fluids and vasopressors, if appropriate |

Alopecia is rare with OXAL alone, but is seen with 5-FU-OXAL combination. Advise patient.

Mild-moderate diarrhea has been seen -- usually of short duration. Treat accordingly. See [Section 8.0](#Sec8p0) for ancillary treatment.

Respiratory problems (i.e., pulmonary fibrosis, cough, dyspnea, rales, pulmonary infiltrates, hypoxia, air hunger and tachypnea) have been observed in patients administered OXAL. In rare cases, death has occurred due to pulmonary fibrosis. Please monitor and instruct the patient to report any respiratory difficulties and hold OXAL until interstitial lung disease is ruled out for cases of Grade ≥ 3. If patient is experiencing shortness of breath, a chest x-ray and assessment of oxygenation via either finger oximetry or arterial blood gas evaluation are required to confirm the absence or presence of pulmonary infiltrates and/or hypoxia (treat accordingly: no intervention, steroids, diuretics, oxygen, or assisted ventilation).

Veno-occlusive disease (VOD) is a rare but serious complication that has been reported in patients receiving oxaliplatin in combination with 5-FU. This condition can lead to hepatomegaly, splenomegaly, portal hypertension and/or esophageal varices. Instruct patients to report any jaundice, ascites, or hematemesis to the MD immediately as these could be a sign of VOD or other serious condition.

Acute vein irritation can occur with infusion. Apply heat to arm of infusion if you are using a peripheral line. However, extravasation of drug can cause severe pain, redness, soreness, and exfoliation of the skin in the affected area with loss of affected vein for a long period. If a patient has a problem with pain or sclerosis when chemotherapy is given by a peripheral line, then placement of a central line should be considered.

Hemolytic Uremic Syndrome (HUS) may result in kidney damage. Oxaliplatin is to be discontinued in cases where hematocrit is <25%, thrombocytopenia <100,000, and creatinine ≥1.6 mg/dL.

Patients may experience sleep disturbances, specifically insomnia. Encourage good sleep hygiene, and instruct patient to report any problems with sleep to the MD, to assess for the potential use of sleeping aids.

Cold-induced transient visual abnormalities can be experienced by patients while receiving OXAL, although the relationship to OXAL has not been completely determined. Instruct patient to report any problems with vision to the MD.

Extrapyramidal side effects and/or involuntary limb movement has been seen with OXAL administration. Patients may also experience restlessness. Instruct patient to report any of these side effects to the MD.

## 10.3 Irinotecan (Camptosar) – NSC #616348

For more information, please refer to the drug package insert.

Procurement

Commercial supplies. Pharmacies or clinics shall obtain supplies from normal commercial supply chain or wholesaler.

Formulation

Commercially available for injection 20 mg/mL (2 mL, 5 mL) [contains sorbitol 45 mg/mL; do not use in patients with hereditary fructose intolerance].

Preparation, Storage and Stability

Refer to package insert for complete preparation and dispensing instructions. Store intact vials at room temperature and protect from light. Doses should be diluted in 250-500 mL D_5_W or 0.9% NaCl to a final concentration of 0.12-2.8 mg/mL. Due to the relatively acidic pH, Irinotecan appears to be more stable in D_5_W than 0.9% NaCl. Solutions diluted in D5W are stable for 24 hours at room temperature or 48 hours under refrigeration at 2ºC to 8ºC. Solutions diluted in 0.9% NaCl may precipitate if refrigerated. Do not freeze.

Administration

Administer by I.V. infusion, usually over 90 minutes.

Drug Interactions

Cytochrome P450 Effect: Substrate (major) of CYP2B6, 3A4

Increased Effect/Toxicity: CYP2B6 and CYP3A4 inhibitors may increase the levels/effects of irinotecan. Bevacizumab may increase the adverse effects of Irinotecan (e.g., diarrhea, neutropenia). Ketoconazole increases the levels/effects of Irinotecan and active metabolite; discontinue ketoconzaole 1 week prior to Irinotecan therapy; concurrent use is contraindicated.

Decreased Effect: CYP2B6 and CYP3A4 inducers may decrease the levels/effects of irinotecan.

Ethanol/Nutrition/Herb Interactions Herb/Nutraceutical: St John’s wort decreases therapeutic effect of irinotecan; discontinue ≥ weeks prior to irinotecan therapy; concurrent use is contraindicated.

Pharmacokinetics

Distribution**:** V_d_: 33-150 L/m^2^

Protein binding, plasma: Predominantly albumin; Parent drug: 30% to 68%, SN-38 (active metabolite): ~95%

Metabolism**:** Primarily hepatic to SN-38 (active metabolite) by carboxylesterase enzymes; SN-38 undergoes conjugation by UDP- glucuronosyl transferase 1A1 (UGT1A1) to form a glucuronide metabolite. Conversion of Irinotecan to SN-38 is decreased and glucoronidation of SN-38 is increased in patients who smoke cigarettes, resulting in lower levels of the metabolite and overall decreased systemic exposure. SN-38 is increased by UGT1A1*28 polymorphism (10% of North Americans are homozygous for UGT1A1*28 allele). Patients homozygous for the UGT1A1*28 allele are at increased risk of neutropenia; initial one-level dose reduction should be considered for both single-agent and combination regimens. The lactones of both Irinotecan and SN-38 undergo hydrolysis to inactive hydroxyl acid forms.

Half-life elimination**:** SN-38: Mean terminal: 10-20 hours

Time to peak: SN-38: Following 90-minute infusion: ~1 hour

Excretion: Within 24 hours: urine: Irinotecan (11% to 20%), metabolites (SN-38 <1%, SN-38 glucuronide, 3%)

Adverse Events

Consult the package insert for the most current and complete information including U.S. Boxed Warnings pertaining to severe diarrhea and severe myelosuppression.

Common known potential toxicities, > 10%:

Cardiovascular: Vasodilation

Central nervous system: Cholinergic toxicity (includes rhinitis, increased salivation, miosis, lacrimation, diaphoresis, flushing and intestinal hyperperistalsis); fever, pain, dizziness, insomnia, headache, chills

Dermatologic: Alopecia, rash

Endocrine & metabolic: Dehydration

Gastrointestinal: Late onset diarrhea, early onset diarrhea, nausea, abdominal pain, vomiting, cramps, anorexia, constipation, mucositis, weight loss, flatulence, stomatitis

Hematologic: Anemia, leukopenia, thrombocytopenia, neutropenia

Hepatic: Bilirubin increased, alkaline phosphatase increased

Neuromuscular & skeletal: Weakness, back pain

Respiratory: Dyspnea, cough, rhinitis

Miscellaneous: Diaphoresis, infection

Less common known potential toxicities, 1% - 10%:

Cardiovascular: Edema, hypotension, thromboembolic events

Central nervous system: Somnolence, confusion

Gastrointestinal: Abdominal fullness, dyspepsia

Hematologic: Neutropenic fever, hemorrhage, neutropenic infection

Hepatic: AST increased, ascites and/or jaundice

Respiratory: Pneumonia

Rare known potential toxicities, <1% (Limited to important or life-threatening):

ALT increased, amylase increased, anaphylactoid reaction, anaphylaxis, angina, arterial thrombosis, bleeding, Bradycardia, cardiac arrest, cerebral infarct, cerebrovascular accident, circulatory failure, colitis, deep thrombophlebitis, dysrhythmia, embolus, gastrointestinal bleeding, gastrointestinal obstruction, hepatomegaly, hiccups, hyperglycemia, hypersensitivity, hyponatremia, ileus, interstitial lung disease, intestinal perforation, ischemic colitis, lipase increased, lymphocytopenia, megacolon, MI, muscle cramps, myocardial ischemia, pancreatitis, paresthesia, peripheral vascular disorder, pulmonary embolus, pulmonary toxicity (dyspnea, fever, reticulnodular infiltrates on chest x-ray), renal failure (acute), renal impairment, syncope, thrombophlebitis, thrombosis, typhlitis, ulceration, ulcerative colitis, vertigo

Nursing Guidelines

If possible, check for any history of hypersensitivity reaction to any previous drug formulated with polysorbate 80.

Cholinergic symptoms of lacrimation, nasal congestion, diaphoresis, flushing, ABD cramping, and diarrhea can occur at the beginning, during, or immediately after the CPT-11 infusion. It is suggested that the patient remain in the treatment area for a minimum of one hour following the completion of the very first CPT-11 infusion. If diarrhea occurs within one hour of infusion, refer to [Section 8.1.5](#Sec8p1p5) for management.

Patient education is extremely important. Impress on the patient the importance of compliance with treatment of diarrhea management. Stress the need for prompt recognition and early intervention. Motivate the patient to report any complications immediately. The cholera-like syndrome can be unresponsive to conventional antidiarrheals and can result in severe dehydration.

Ondanestron and diphenhydramine should provide good relief from the nausea/vomiting/cramping. Avoid prochlorperazine on the day of treatment due to its association with akathisia (motor restlessness). Prochlorperazine may be taken between treatments.

Advise avoidance of excess caffeine, a GI stimulant. Avoid magnesium-based antacids such as Mylanta, Maalox, Rolaids, MOM, Mag-Ox 400, and Tylenol with antacid.

The pulmonary toxicity seen is usually manifested by dyspnea beginning 42-175 days after treatment and occurs at a cumulative dose ranging from 400-1000 mg/m (median 750). Instruct patient to report any cough or SOB.

Patients are at risk for developing eosinophilia and will improve on steroid therapy.

Hepatic enzyme elevations have been transient and did not require intervention.

Monitor CBC closely. Leukopenia occurs primarily as neutropenia but can be severe and dose limiting. The simultaneous occurrence of grade 4 diarrhea and grade 4 neutropenia is rare but may render the patient more susceptible to polymicrobial sepsis and potentially death.

Advise patients of probable hair loss**.**

## 10.4 Leucovorin Calcium – NSC #3590

For more information, please refer to the drug package insert.

Procurement

Commercial supplies. Pharmacies or clinics shall obtain supplies from normal commercial supply chain or wholesaler.

Formulation

Commercially available:

Solution for Injection 100 mg/10mL (10mL, 30 mL)

Lyophilized Powder for Injection 50 mg, 100 mg, 200 mg, 350 mg, 500 mg

Preparation, Storage and Stability

Powder for injection: Store at room temperature of 25°C (77°F). Protect from light. Solutions reconstituted with bacteriostatic water for injection U.S.P., must be used within 7 days. Solutions reconstituted with SWFI must be used immediately. Parenteral admixture is stable for 24 hours stored at room temperature (25°C) and for 4 days when stored under refrigeration (4°C). Powder for injection: Reconstitute with SWFI or BWFI; dilute with D5W or NS for infusion. When doses > 10 mg/m^2^ are required, reconstitute using sterile water for injection, not a solution containing benzyl alcohol.

Solution for injection: Prior to dilution, store vials under refrigeration at 2°C to 8°C (36°F to 46°F). Protect from light. Dilute in D5W or NS for infusion.

Administration

Combination therapy with fluorouracil: Fluorouracil is usually given after, or at the midpoint, of the leucovorin infusion. Leucovorin is usually administered by IV bolus injection or short (10-120 minutes) IV infusion. Other administration schedules have been used; refer to individual protocols.

Drug Interactions

Capecitabine: Leucovorin Calcium-Levoleucovorin may enhance the adverse/toxic effect of Capecitabine. Risk C: Monitor therapy

Fluorouracil (Systemic): Leucovorin Calcium-Levoleucovorin may enhance the adverse/toxic effect of Fluorouracil (Systemic). This effect is associated with the ability of leucovorin or levoleucovorin to enhance the anticancer effects of fluorouracil. Risk C: Monitor therapy

Fluorouracil (Topical): Leucovorin Calcium-Levoleucovorin may enhance the adverse/toxic effect of Fluorouracil (Topical). Risk C: Monitor therapy

Fosphenytoin: Leucovorin Calcium-Levoleucovorin may decrease the serum concentration of Fosphenytoin. Risk C: Monitor therapy

PHENobarbital: Leucovorin Calcium-Levoleucovorin may decrease the serum concentration of PHENobarbital. Risk C: Monitor therapy

Phenytoin: Leucovorin Calcium-Levoleucovorin may decrease the serum concentration of Phenytoin. Risk C: Monitor therapy

Primidone: Leucovorin Calcium-Levoleucovorin may decrease the serum concentration of Primidone. Additionally, leucovorin/levoleucovorin may decrease concentrations of active metabolites of primidone (e.g., phenobarbital). Risk C: Monitor therapy

Raltitrexed: Leucovorin Calcium-Levoleucovorin may diminish the therapeutic effect of Raltitrexed. Risk X: Avoid combination

Tegafur: Leucovorin Calcium-Levoleucovorin may enhance the adverse/toxic effect of Tegafur. This effect is associated with the ability of leucovorin or levoleucovorin to enhance the anticancer effects of fluorouracil. Risk C: Monitor therapy

Trimethoprim: Leucovorin Calcium-Levoleucovorin may diminish the therapeutic effect of Trimethoprim. Management: Avoid concurrent use of leucovorin or levoleucovorin with trimethoprim (plus sulfamethoxazole) for Pneumocystis jiroveci pneumonia. If trimethoprim is used for another indication, monitor closely for reduced efficacy. Risk X: Avoid combination

Pharmacokinetics

Absorption: Oral, IM: Well absorbed

Metabolism: Intestinal mucosa and hepatically to 5-methyl-tetrahydrofolate (5MTHF; active)

Bioavailability: Saturable at oral doses >25 mg; 25 mg (97%), 50 mg (75%), 100 mg (37%)

Half-life elimination: ~4-8 hours

Time to peak: Oral: ~2 hours; IV: Total folates: 10 minutes; 5MTHF: ~1 hour

Excretion: Urine (primarily); feces

Adverse Events

Consult the package insert for the most current and complete information.

Dermatologic: Rash, pruritus, erythema, urticaria

Hematologic: Thrombocytosis

Respiratory: Wheezing

Miscellaneous: Allergic reactions, anaphylactoid reactions

Nursing Guidelines

Headache may occur. Advise patient that analgesics such as Tylenol may help. Instruct patient to report any headache that is unrelieved.

Observe for sensitization reaction (rash, hives, pruritus, facial flushing, and wheezing).

May potentiate the toxic effects of fluropyrimidine (5-FU) therapy, resulting in increased hematologic and gastrointestinal (diarrhea, stomatitis) adverse effects. Monitor closely.

May cause mild nausea or upset stomach. Administer antiemetics if necessary and evaluate for their effectiveness.

## 10.5 5-Fluorouracil (5-FU) (Efudex, Arucil, Carac, Fluroplex) – NSC #19893

For more information, please refer to the drug package insert.

Commercial supplies. Pharmacies or clinics shall obtain supplies from normal commercial supply chain or wholesaler.

Formulation

Commercially available as Intravenous Solution: 500 mg/10 mL (10 mL); 1 g/20 mL (20 mL); 2.5 g/50 mL (50 mL); 5 g/100 mL (100 mL)

Preparation, Storage and Stability

Store intact vials at room temperature and protect from light. A slight discoloration may occur with storage but usually does not denote decomposition. Dilute in 50 – 1000 mL of 0.9% NaCl or D5W. If exposed to cold, a precipitate may form; gentle heating to 60ºC will dissolve the precipitate without impairing the potency. Solutions in 50 – 1000 mL 0.9% NaCl or D5W or undiluted solutions in syringes are stable for 72 hours at room temperature. Fluorouracil should not be coadministered with either diazepam, doxorubicin, daunorubicin, idarubicin, cisplatin, or cytarabine. However, fluorouracil and leucovorin are compatible for 14 days at room temperature. Fluorouracil is compatible with vincristine, methotrexate, and cyclophosphamide.

Administration

Fluorouracil may be given IV push, IV infusion. Refer to the treatment section for specific administration instructions. Avoid extravasation, may be an irritant.

Drug Interactions

Fluorouracil may increase effects of warfarin. Avoid ethanol (due to GI irritation). Avoid black cohosh.

Pharmacokinetics

**Distribution:** V_d_ ~ 22% of total body water; penetrates extracellular fluid, CSF, and third space fluids (e.g., pleural effusions and ascitic fluid)

**Metabolism:** Hepatic (90%); via a dehydrogenase enzyme; Fluorouracil must be metabolized to be active.

**Half-life elimination:** Biphasic: Initial: 6-20 minutes; two metabolites, FdUMP and FUTP, have prolonged half-lives depending on the type of tissue.

**Excretion**: Lung (large amounts as CO_2_); urine (5% as unchanged drug) in 6 hours.

Adverse Events

Consult the package insert for the most current and complete information.

Common known potential toxicities, > 10%:

Dermatologic: Dermatitis, pruritic maculopapular rash, alopecia.

Gastrointestinal (route and schedule dependent): Heartburn, nausea, vomiting, anorexia, stomatitis, esophagitis, anorexia, diarrhea. GI toxicity (anorexia, nausea, and vomiting) is generally more severe with continuous-infusion schedules.

Emetic potential: <1000 mg: Moderately low (10% to 30%) ≥ 1000 mg: Moderate (30% to 60%)

Hematologic: Leukopenia; Myelosuppressive (tends to be more pronounced in patients receiving bolus dosing of FU). Decreased white blood cell count with increased risk of infection; decreased platelet count with increased risk of bleeding.

Local: Irritant chemotherapy.

Less common known potential toxicities, 1% - 10%:

Dermatologic: Dry skin

Gastrointestinal: GI ulceration

**Rare known potential toxicities, <1%** (Limited to important or life-threatening):

Cardiac enzyme abnormalities, chest pain, coagulopathy, dyspnea, ECG changes similar to ischemic changes, hepatotoxicity; hyperpigmentation of nail beds, face, hands, and veins used in infusion; hypotension, palmar-plantar syndrome (hand-foot syndrome), photosensitization. Cerebellar ataxia, headache, somnolence, ataxia are seen primarily in intracarotid arterial infusions for head and neck tumors.

Nursing Guidelines

Monitor complete blood count and platelet count. Instruct patient to report signs and symptoms of infection, unusual bruising or bleeding to the physician.

Administer antiemetics as indicated.

Diarrhea may be dose–limiting; encourage fluids and treat symptomatically.

Assess for stomatitis; oral care measures as indicated. May try vitamin E oil dabbed on sore, six times daily. Cryotherapy recommended with IV push administration.

Monitor for neurologic symptoms (headache, ataxia).

Inform patient of potential alopecia.

Those patients on continuous infusion may need instruction regarding central intravenous catheters and portable intravenous or IA infusion devices.

5FU–induced conjunctivitis is a common problem. Advise patient to report any eye soreness or redness to the healthcare team.

Photosensitivity may occur. Instruct patients to wear sun block when outdoors.

# 11.0 Measurement of Effect

Response and progression will be evaluated in this study using the new international criteria proposed by the revised Response Evaluation Criteria in Solid Tumors (RECIST) guidelines (version 1.1) [38]*.* Changes in the largest diameter (unidimensional measurement) of the tumor lesions and the short axis measurements in the case of lymph nodes are used in the RECIST guideline*.*

## 11.1 Schedule of Evaluations:

Restaging cross sectional imaging studies are performed as scheduled throughout the study timeline:

- Prior to treatment (includes real-time central review),
- Following 4 cycles of neoadjuvant chemotherapy,
- Following 3 cycles of neoadjuvant chemotherapy and radiation (Arm 1) and before surgery

OR

- Following 4 cycles of neoadjuvant chemotherapy (Arm 2) and before surgery
- After surgery and prior to postoperative chemotherapy
- After 4 cycles of adjuvant chemotherapy
- Surveillance per the schedule outlined in the study calendar in [Section 5.0](#Sec5p0).

Supporting documentation of surgical and pathologic response, and progression should be submitted, per [Section 6.1.1](#Sec6p1p1).

## 11.2 Definitions of Measurable and Non-Measurable Disease

11.2.1 Measurable Disease

A non-nodal lesion is considered measurable if its longest diameter can be accurately measured as ≥2.0 cm with chest x-ray, or as ≥1.0 cm with CT scan or MRI.

A superficial non-nodal lesion is measurable if its longest diameter is ≥ 1.0 cm in diameter as assessed using calipers (e.g. skin nodules) or imaging. In the case of skin lesions, documentation by color photography, including a ruler to estimate the size of the lesion, is recommended.

A malignant lymph node is considered measurable if its short axis is >1.5 cm when assessed by CT scan (CT scan slice thickness recommended to be no greater than 5 mm).

11.2.2 Non-Measurable Disease

All other lesions (or sites of disease) are considered non-measurable disease, including pathological nodes (those with a short axis ≥ 1.0 to < 1.5 cm). Bone lesions, leptomeningeal disease, ascites, pleural/pericardial effusions, lymphangitis cutis/pulmonis, inflammatory breast disease, and abdominal masses (not followed by CT or MRI), are considered as non-measurable as well.

Note: ‘Cystic lesions’ thought to represent cystic metastases can be considered as measurable lesions, if they meet the definition of measurability described above. However, if non-cystic lesions are present in the same patient, these are preferred for selection as target lesions. In addition, lymph nodes that have a short axis < 1.0 cm are considered non-pathological (i.e., normal) and should not be recorded or followed.

## 11.3 Guidelines for Evaluation of Measurable Disease

11.3.1 Measurement Methods:

• All measurements should be recorded in metric notation (i.e., decimal fractions of centimeters) using a ruler or calipers.

• The same method of assessment and the same technique must be used to characterize each identified and reported lesion at baseline and during follow-up. For patients having only lesions measuring at least 1 cm to less than 2 cm must use CT imaging for both pre- and post-treatment tumor assessments.

• Imaging-based evaluation is preferred to evaluation by clinical examination when both methods have been used at the same evaluation to assess the antitumor effect of a treatment.

11.3.2 Acceptable Modalities for Measurable Disease:

**• Conventional CT and MRI:**  This guideline has defined measurability of lesions on CT scan based on the assumption that CT slice thickness is 3 mm or less.

As with CT, if an MRI is performed, the technical specifications of the scanning sequences used should be optimized for the evaluation of the type and site of disease. The lesions should be measured on the same pulse sequence. Ideally, the same type of scanner should be used and the image acquisition protocol should be followed as closely as possible to prior scans. Body scans should be performed with breath-hold scanning techniques, if possible.

**• Chest X-ray:** Lesions on chest x-ray are acceptable as measurable lesions when they are clearly defined and surrounded by aerated lung. However, CT scans are preferable.

11.3.3 Measurement at Follow-up Evaluation:

• The cytological confirmation of the neoplastic origin of any effusion that appears or worsens during treatment when the measurable tumor has met criteria for response or stable disease is mandatory to differentiate between response or stable disease (an effusion may be a side effect of the treatment) and progressive disease.

• Cytologic and histologic techniques can be used to differentiate between PR and CR in rare cases (e.g., residual lesions in tumor types such as germ cell tumors, where known residual benign tumors can remain.)

## 11.4 Measurement of Treatment/Intervention Effect

11.4.1 Target Lesions & Target Lymph Nodes

• Measurable lesions (as defined in [Section 11.2.1](#Sec11p2p1)) up to a maximum of 5 lesions, representative of all involved organs, should be identified as “Target Lesions” and recorded and measured at baseline. These lesions can be non-nodal or nodal (as defined in [11.2.1](#Sec11p2p1)), where no more than 2 lesions are from the same organ and no more than 2 malignant nodal lesions are selected.

**Note:** If fewer than 5 target lesions and target lymph nodes are identified (as there often will be), there is no reason to perform additional studies beyond those specified in the protocol to discover new lesions.

• Target lesions and target lymph nodes should be selected on the basis of their size, be representative of all involved sites of disease, but in addition should be those that lend themselves to reproducible repeated measurements. It may be the case that, on occasion, the largest lesion (or malignant lymph node) does not lend itself to reproducible measurements in which circumstance the next largest lesion (or malignant lymph node) which can be measured reproducibly should be selected.

• **Baseline Sum of Dimensions (BSD):** A sum of the longest diameter for all target lesions plus the sum of the short axis of all the target lymph nodes will be calculated and reported as the baseline sum of dimensions (BSD). The BSD will be used as reference to further characterize any objective tumor response in the measurable dimension of the disease.

• **Post-Baseline Sum of the Dimensions (PBSD):** A sum of the longest diameter for all target lesions plus the sum of the short axis of all the target lymph nodes will be calculated and reported as the post-baseline sum of dimensions (PBSD). If the radiologist is able to provide an actual measure for the target lesion (or target lymph node), that should be recorded, even if it is below 0.5 cm. If the target lesion (or target lymph node) is believed to be present and is faintly seen but too small to measure, a default value of 0.5 cm should be assigned. If it is the opinion of the radiologist that the target lesion or target lymph node has likely disappeared, the measurement should be recorded as 0 cm.

• **The minimum sum of the dimensions (MSD)** is the minimum of the BSD and the PBSD.

11.4.2 Non-Target Lesions & Non-Target Lymph Nodes

Non-measurable sites of disease ([Section 11.2.2](#Sec11p2p2)) are classified as non- target lesions or non-target lymph nodes and should also be recorded at baseline. These lesions and lymph nodes should be evaluated in accordance with [Section 11.4.3](#Sec11p4p3).

11.4.3 Response Criteria

All target lesions and target lymph nodes followed by CT/MRI/Chest X-ray/physical examination must be measured on re-evaluation at the evaluation time points specified in [Section 11.1](#Sec11p1). Specifically, a change in objective status to either a PR or CR cannot be done without re-measuring target lesions and target lymph nodes.

Note: Non-target lesions and non-target lymph nodes should be evaluated at each assessment, especially in the case of first response or confirmation of response. In selected circumstances, certain non-target organs may be evaluated less frequently. For example, bone scans may need to be repeated only when complete response is identified in target disease or when progression in bone is suspected.

Evaluation of Target Lesions

**Complete Response (CR):** All of the following must be true:

a. Disappearance of all target lesions.

b. Each target lymph node must have reduction in short axis to < 1.0 cm.

**Partial Response (PR):** At least a 30% decrease in PBSD (sum of the longest diameter for all target lesions plus the sum of the short axis of all the target lymph nodes at current evaluation) taking as reference the BSD (see [Section 11.4.1](#Sec11p4p1)).

**Progression (PD):** At least one of the following must be true:

a. At least one new malignant lesion, which also includes any lymph node that was normal at baseline (< 1.0 cm short axis) and increased to ≥ 1.0 cm short axis during follow-up.

b. At least a 20% increase in PBSD (sum of the longest diameter for all target lesions plus the sum of the short axis of all the target lymph nodes at current evaluation) taking as reference the MSD ([Section 11.4.1](#Sec11p4p1)). In addition, the PBSD must also demonstrate an absolute increase of at least 0.5 cm from the MSD.

**Stable Disease (SD):** Neither sufficient shrinkage to qualify for PR, nor sufficient increase to qualify for PD taking as reference the MSD.

Evaluation of Non-Target Lesions & Non-target Lymph Nodes

**Complete Response (CR):** All of the following must be true:

a. Disappearance of all non-target lesions.

b. Each non-target lymph node must have a reduction in short axis to <1.0 cm.

**Non-CR/Non-PD:** Persistence of one or more non-target lesions or non-target lymph nodes.

**Progression (PD):** At least one of the following must be true:

a. At least one new malignant lesion, which also includes any lymph node that was normal at baseline (< 1.0 cm short axis) and increased to ≥ 1.0 cm short axis during follow-up.

b. Unequivocal progression of existing non-target lesions and non-target lymph nodes. (NOTE: Unequivocal progression should not normally trump target lesion and target lymph node status. It must be representative of overall disease status change.)

11.4.3.1 Evaluation of Elevated CA19-9 Levels (when present)

Note: this evaluation will be done for each patient for descriptive purposes only and will not be used to evaluate the secondary endpoint of progression free survival. Decision making for the efficacy of therapy and for relapse or progression must be based on imaging. Biochemical progression without the demonstration of objective progression of target or non-target lesion will not be sufficient indication to remove the patient from the study (refer to [Section 13.1](#Sec13p1)).

**Biochemical response (B-RESP):** Reduction in elevated CA 19-9 level by at least 50% of normalization of an elevated value, confirmed with 2 successive measures.

**Biochemical progression (B-PROG):** Increase in elevated CA 19-9 level by 50%, confirmed with 2 successive measures. If patient has had biochemical response as defined above, then subsequent biochemical progression is defined as >50% increase from the lowest recorded level while on study.

**Biochemical stability (B-STAB):** Elevated CA 19-9 level does not change enough to qualify as response or progression.

11.4.4 Overall Objective Status

The overall objective status for an evaluation is determined by combining the patient’s status on target lesions, target lymph nodes, non-target lesions, non-target lymph nodes, and new disease as defined in the following tables:

| **For Patients with Measurable Disease** | | | |
| --- | --- | --- | --- |
| **Target Lesions &**  **Target Lymph Nodes** | **Non-Target Lesions &**  **Non-Target Lymph Nodes** | **New**  **Sites of Disease** | **Overall Objective Status** |
| CR | CR | No | CR |
| CR | Non-CR/Non-PD | No | PR |
| PR | CR  Non-CR/Non-PD | No | PR |
| CR/PR | Not All Evaluated* | No | PR** |
| SD | CR  Non-CR/Non-PD  Not All Evaluated* | No | SD |
| Not all Evaluated | CR  Non-CR/Non-PD  Not All Evaluated* | No | Not Evaluated (NE) |
| PD | Unequivocal PD  CR  Non-CR/Non-PD  Not All Evaluated* | Yes or No | PD |
| CR/PR/SD/PD/Not all Evaluated | Unequivocal PD | Yes or No | PD |
| CR/PR/SD/PD/Not all Evaluated | CR  Non-CR/Non-PD  Not All Evaluated* | Yes | PD |

* See [Section 11.4.3](#Sec11p4p3).

| **For Patients with Non-Measurable Disease Only** | | |
| --- | --- | --- |
| **Non-Target Lesions &**  **Non-Target Lymph Nodes** | **New**  **Sites of Disease** | **Overall**  **Objective Status** |
| **CR** | **No** | **CR** |
| **Non-CR/Non-PD** | **No** | **Non-CR/Non-PD** |
| **Not All Evaluated*** | **No** | **Not Evaluated (NE)** |
| **Unequivocal PD** | **Yes or No** | **PD** |
| **Any** | **Yes** | **PD** |

* See [Section 11.4.3](#Sec11p4p3).

11.4.5 Symptomatic Deterioration: Patients with global deterioration of health status requiring discontinuation of treatment without objective evidence of disease progression at that time, and not either related to study treatment or other medical conditions, should be reported as PD due to “symptomatic deterioration.” Every effort should be made to document the objective progression even after discontinuation of treatment due to symptomatic deterioration.

## 11.5 Definitions of Analysis Variables

Formal definitions of variables used in analyses can be found in the Statistical Considerations section of the protocol.

## 11.6 Surgical Pathologic Determination of Treatment Response

Pathologic response to neoadjuvant therapy will be documented at the site by using the information from the local operative report and local pathology report. This will be utilized at the site for individual patient care, and the information will also be included in the statistical analysis in the secondary endpoint of R0 resection. See [Section 11.7](#Sec11p7) for further information.

In addition, there will be a retrospective central pathology review that documents the pathologic response to neoadjuvant therapy that will be included in the statistical analysis, but will NOT be utilized for the management of the individual patient.

Histopathologic response to preoperative therapy will be determined by analysis of the resected surgical specimen by the pathologist (see [Section 7.3](#Sec7p3)). The following grades will be used to semi-quantitatively characterize treatment response:

I - 0% residual tumor cells in the specimen (pCR)

II - 1 to <5% residual tumor cells in the specimen

III - ≥5% residual tumor cells in the specimen

## 11.7 Histopathologic Determination of R-status

11.7.1 “Macroscopic disease” is assessed by surgeon and “microscopic disease” is assessed by the histopathologist.

11.7.2 Margins to be assessed in this trial include: 1) common bile duct, 2) SMA margin, 3) pancreatic neck as described in pathology section:

R0- Macroscopically complete tumor removal with negative microscopic surgical margins.

R1- Macroscopically complete tumor removal with positive microscopic margins (any or all).

R2- Macroscopically incomplete tumor removal with known or suspected residual gross disease.

## 11.8 Treatment Evaluation after Completion of Surgery and Post-operative Chemotherapy

11.8.1 The term “recurrence” is used if cancer returns either in the locoregional area of the primary tumor or at distant sites following R0 or R1 resection.

11.8.2 Recurrence is determined radiographically and biopsy need not be performed except as detailed below or when CT findings are equivocal in the determination of the enrolling physician.

**Locoregional recurrence**: identified by a new soft-tissue mass around the mesenteric vasculature or regional lymph nodes. Note that soft-tissue in this location on early postoperative scans may be benign and due to postoperative changes. Such findings may be difficult to distinguish from early tumor progression. In cases in which the diagnosis is ambiguous, discussion with the PI should occur. Performance of tissue biopsy should be considered prior to assigning a diagnosis of locoregional recurrent disease.

**Distant recurrence**: identified by a new hypodensity in the liver or nodule(s) in the lungs or peritoneum. Other sites of presumptive distant recurrence (adrenal, brain) should require prompt biopsy confirmation. New ascites should be interrogated with aspiration and cytology if it is an isolated finding and should not necessarily be taken to indicate carcinomatosis, particularly following the performance of resection and reconstruction of the mesenteric vein(s).

# 12.0 End of Treatment/Intervention

## 12.1 Duration of Treatment

12.1.1 CR, PR, or SD: Patients who are in CR, PR or SD will continue on therapy until disease progression (See [Section 12.1.3](#Sec12p1p3)), excess toxicity or patient withdrawal. After treatment is discontinued, patients will be followed per the study calendar in [Section 5.0](#Sec5p0).

12.1.2 Disease Progression:

Remove from protocol therapy any patient with disease progression as per [Section 12.1.3](#Sec12p1p3) or recurrence after surgery (either Arm 1 or Arm 2 patients). Document details, including tumor measurements, on data forms.

After disease progression, patients should be followed for survival per the study calendar ([Section 5.0](#Sec5p0)).

Note: Biochemical progression (B-PROG) without the demonstration of objective progression (PD) of target or non-target lesion will not be sufficient indication to remove the patient from the study (see [Section 11.4.3](#Sec11p4p3)).

12.1.3 Removal of Patients from Protocol Therapy

In the absence of treatment delays due to adverse events, treatment will be completed unless the patient experiences any one of the following scenarios. The specific indication should be described as:

- Metastatic disease identified on imaging studies
- For patients with RECIST PD with local progression only, the treating investigator should decide whether continuing protocol treatment is in the best interest of the patient
- The presence of locally advanced disease on pre-surgical restaging imaging studies (See [Appendix II](#AppII) for definition of locally advanced pancreatic cancer)
- Decline in PS in the absence of radiographic evidence for disease progression if PS cannot be recovered (symptomatic deterioration)
- Metastatic disease identified intraoperatively and biopsy-proven
- Unresectable disease identified intraoperatively (e.g., locally infiltrative disease without metastases for which the surgeon does not feel resection is safe)
- R2 surgical resection
- Nontherapeutic (aborted) operation for any other reason
- Intercurrent illness that prevents further administration of treatment
- Unacceptable adverse event(s)
- Patient decides to withdraw from the study
- General or specific changes in the patient’s condition that render the patient unacceptable for further treatment in the judgment of the investigator

Patient should be removed from protocol therapy, and followed as per [Section 5.0](#Sec5p0), post-treatment follow-up. Protocol treatment will be discontinued and further treatment is at the discretion of the treating physician.

12.1.4 Follow-up for patients removed from protocol therapy

For patients who are removed from protocol therapy in any instance listed in [Section 12.1.2](#Sec12p1p2), follow patients per the schedule found in [Section 5.0](#Sec5p0) of the protocol for clinical and survival status.

## 12.2 Managing Ineligible Patients and Registered Patients Who Never Receive Protocol Intervention

12.2.1 Definition of ineligible patient

A study participant who is registered to the trial but does not meet all of the eligibility criteria is deemed to be ineligible.

12.2.2 Follow-up for ineligible patients who continue with protocol treatment

Patients who are deemed ineligible after registering may continue protocol treatment, provided the treating physician, study chair, and executive officer agree there are no safety concerns if the patient continues protocol treatment. All scans, tests, and data submission are to continue as if the patient were eligible. Notification of the local IRB may be necessary per local IRB policies.

12.2.3 Follow-up for ineligible patients who discontinue protocol treatment

For patients who are deemed ineligible after registering to the trial, who start treatment, but then discontinue study treatment, the same data submission requirements are to be followed as for those patients who are eligible and who discontinue study treatment.

12.2.4 Follow-up for patients who are registered, but who never start study treatment

For all study participants who are registered to the trial but who never receive study intervention (regardless of eligibility), the follow-up requirements are specified below.

Baseline, off treatment, and post-treatment follow up (i.e., relapse, progression, and survival) data submission is required. See the Data Submission Schedule accompanying the All Forms Packet.

## 12.3 Extraordinary Medical Circumstances

If, at any time the constraints of this protocol are detrimental to the patient's health and/or the patient no longer wishes to continue protocol therapy, protocol therapy shall be discontinued. In this event:

- Document the reason(s) for discontinuation of therapy on data forms.
- Follow the patient for protocol endpoints as required by the Study Calendar.

# 13.0 Statistical Considerations

## 13.1 Study Endpoints

13.1.1 Primary Endpoint

The primary endpoint of this study is the 18-month OS rate, defined as the number of patients who are alive at 18 months after randomization divided by the total number of evaluable patients in each arm. An evaluable patient is defined as any patient who signed informed consent, deemed eligible by central review and received any protocol-defined treatment.

13.1.2 Secondary Endpoints

13.1.2.1 R0 resection rate

R0 resection rate is defined as the proportion of patients in whom an achieved R0 resection was achieved during surgery (see [Section 11.7.2](#Sec11p7p2)). The R0 resection rate will be estimated in two cohorts, 1) patients who signed the consent form, deemed eligible by central review, and received any protocol defined treatment in each arm and 2) patients who signed the consent form, deemed eligible by central review, and underwent surgery in each arm. The R0 resection rate will be estimated by the number of patients in whom R0 resection was performed divided by total number of patients in cohort 1) and cohort 2) in each arm.

13.1.2.2 Event-free survival

Event-free survival is defined as time from randomization to the first documentation of event where events considered are 1) disease progression, per RECIST, prior to surgery, 2) surgery with R2 resection, 3) recurrent disease following surgery, or 4) death due to any cause.

13.1.2.3 Pathologic complete response (pCR) rate

Pathologic complete response (pCR) rate is defined as the proportion of patients in whom a pCR was confirmed by histopathologic review of the surgical specimen (see [Section 11.6](#Sec11p6)) The pCR rate will be estimated in two cohorts, 1) patients who signed the consent form, deemed eligible by central review, and received any protocol-defined treatment in each arm and 2) patients who signed the consent form, deemed eligible by central review, and underwent surgery. The pCR rate will be estimated by the number of patients in whom a pCR was confirmed divided by total number of patients in cohort 1) and cohort 2) in each arm.

13.1.2.4 Adverse events (AE)

AEs and the maximum grade for each type of adverse events will be recorded for each patient separately for the following 3 periods:

1. During neoadjuvant chemotherapy and chemoradiation therapy
2. Surgical complications during surgery and post-op period for 90 days or prior to adjuvant therapy
3. During adjuvant therapy. See [Section 9.1](#Sec9p1).

Similarly, scores (0-4) and maximum score for each PRO-CTCAE item will be recorded for each patient separately for the 3 periods.

## 13.2 Sample Size

We anticipate enrolling a maximum of 124 evaluable patients (62 per arm) per statistical design. Additional 10 (5 per arm) patients will be accrued to account for cancelations, ineligibilities, major violations, and lost-to-follow up, etc. Thus the total targeted accrual will be 134 patients.

We anticipate to pre-register 5 patients per month. Since we estimate 80% of patients who are pre-registered for central review will be randomized to therapy, the actual accrual will be 4 patients per month. Thus, we expect to pre-register 168 patients to reach the targeted accrual of 134 patients. The accrual period of this study is estimated to be 34 months.

We also expect that 40% of patients randomized to therapy will be off protocol treatment prior to surgery (i.e. will not undergo surgery). Therefore we expect that of the 134 patients, 80 will proceed to surgery. The primary endpoint, overall survival at 18 month post randomization, is well-defined even among patients who do not receive surgery. Therefore, the percentages of patients who do not undergo surgery do not affect the trial design.

## 13.3 Power Justification

The primary aim of this randomized phase II study is to evaluate 18-month OS rate in patients with borderline resectable cancer of the head of the pancreas treated neoadjuvantly using one of the following regimens: 1) Arm 1: 8 cycles of systemic FOLFIRINOX or 2) Arm 2: 7 cycles - of systemic FOLFIRINOX followed by short-course hypofractionated radiation therapy. All patients who are able to undergo subsequent surgery will also receive 4 cycles of postoperative FOLFOX. Eligible patients will be randomized and stratified by ECOG performance score onto two arms. Within each arm, single-arm sequential design with one interim analysis for futility will be implemented. The final efficacy analysis and interim analysis will be based on 18-month OS rate and R0 resection rate, respectively, and evaluated in each arm separately. The comparison of OS between arms will be carried out only if both arms are deemed promising at the end of the trial. “Pick-a-winner” strategy will be implemented to choose one treatment strategy for recommendation.

An extensive literature review was conducted to evaluate median OS and R0 resection rate in patients purported to have “borderline resectable” tumors who received chemo, chemoRT, etc. were conducted. Twenty-six studies published between 2004 and 2015 are identified with number of borderline resectable patients range from 8 to 129. All but three of these studies are retrospective studies.

The following table summarizes the historical data of median OS and R0 resection rate among all patients started treatment, including both patients with and without surgery.

|  | **Among studies with N > 20** | | | | **Among studies with N > 50** | | | |
| --- | --- | --- | --- | --- | --- | --- | --- | --- |
| **Time era** | **N of studies** | - **Weighted** | **Median** | **Range (IQR)** | **N of studies** | - **Weighted** | - **Median** | **Range**  **(IQR)** |
| **Median OS in months** | | | | | | | | |
| **Prior to 2012** | 6 | - 21.3 | 21.5 | 14.2 – 25 (18.0 – 22.8) | 3 | - 22.0 | 22.0 | 14.2 – 25 (21.5 – 22.5) |
| **2012 and later** | 9 | - 21.2 | 19.9 | 17.2 – 28 (18.0 – 23.6) | 6 | - 21.2 | 21.0 | 17.2 – 28 (17.9 – 23.2) |
| **All studies** | 15 | - 21.2 | 21.0 | 14.2 – 28 (17.6 – 23.3) | 9 | - 21.5 | 22.0 | 15 – 28 (19.0 – 23.0) |
| **R0 resection rate among all patients started neoadjuvant treatment** | | | | | | | | |
| **Prior to 2012** | 6 | 41.4% | 36.9% | 30 – 59% | 3 | 43.0% | 38.8% | 30 – 59% |
| **2012 and later** | 9 | 61.2% | 54.4% | 42 – 92% | 6 | 60.0% | 54.2% | 42 – 92% |
| **All studies** | 15 | 53.0% | 53.4% | 30 – 92% | 9 | 52.8% | 53.9% | 37 – 92% |

We consider studies with reasonable number of borderline resectable patients (e.g., N > 20, and/or N > 50) to estimate the historical estimates of the median OS and R0 resection rate for sample size and power determinations. There is substantial variability in the literature reported median OS across studies. The median OS reported in these studies varies from 14 to 28 months, with a median of 21 to 22 months and an interquartile range of about 18 to 23 months. Most of the previous studies are retrospective single-institution studies subject to patient selection bias, which likely to skew the reported OS higher. Thus, we consider the median OS of at most 18 months (equivalent to 18m OS rate of 0.50, assuming exponential survival function) which is the lower bound of the IQR of literature reported data as the null hypothesis. To demonstrate clinical meaningful improvement in OS, we target an alternative hypothesis of median OS of at least 27 months (equivalent to 18m OS rate of 0.63; a 50% increase in median OS time).

The summary data in the table shows clear increase trend of R0 resection rate over time. For interim analysis based on R0 resection rate, if R0 resection rate is at least 60% (null hypothesis) based on weighted R0 resection rate from historical data, the arm warrants continuing to full accrual for OS final analysis. If the observed R0 resection rate is significantly less than 60%, we consider early futility of the arm and stop accrual of patients onto that arm. Therefore, the interim analysis based on R0 resection rate will be testing alternative hypothesis of R0 resection rate ≤ 40% against the null hypothesis of R0 resection rate ≥ 60%.

The study operating characteristics were assessed by simulation studies based on the estimation procedures and decision rules specified for final and interim analysis described in [Section 13.4.1](#Sec13p4p1). A maximum of 62 evaluable patients in each arm will provide 82% power to detect an improvement of 13% in 18 months OS rate, i.e., testing alternative hypothesis that the 18 months OS rate is at least 63% against the null hypothesis that the 18 months OS rate is at most 50%, at a one-sided significance level of 0.07. The probability of stopping accrual at interim is 43% and 0.8% if the true R0 resection rate is 40% and 60%, respectively.

## 13.4 Statistical Analysis Plan

13.4.1 Primary endpoint

Treatment Efficacy Decision Rules:

13.4.1.1 Interim Analysis

One interim analysis will be performed to assess treatment futility for each arm separately. For each given treatment arm, the R0 resection rate will be evaluated when surgical data become available for first 30 evaluable patients, approximately 8 months after trial activation. If at most 11 patients (i.e., ≤ 11 patients) among 30 evaluable patients underwent R0 resection, then the given treatment arm will be deemed not promising and the accrual to this treatment arm will be terminated. An evaluable patient is defined as any patient who signed informed consent, deemed eligible by central review and received any protocol-defined treatment.

13.4.1.2 Final Analysis

The primary efficacy analysis will be performed on 18-month OS rate for the two treatment arms, separately, based on all evaluable patients, For each given treatment arm, if full accrual is reached and at least 36 patients (i.e. ≥ 36 patients) out of 62 evaluable patients are alive up to 18 months after randomization, the regimen of the evaluated arm will be considered worthy of further testing in this disease population. Otherwise, the regimen will be deemed inefficacious. The final analysis will be carried out approximately 18 months after the last patient, in each arm, is enrolled. This will occur approximately 52 months after trial activation.

13.4.1.3 Analysis plan

The treatment arm specific 18 month OS rate and 95% confidence interval will be estimated based on standard method [39]. Chi-squared test (or Fisher’s exact test if the data in contingency table is sparse) will be used to compare 18 month OS rates among treatment arms. Overall survival within each arm will be summarized by Kaplan-Meier method. Median, 1-year and 2-year rates will be estimated based on Kaplan-Meier curves.

If both arms are deemed worthy of further testing in this disease population, a log-rank test will be used to compare the median OS between the two arms. A “pick-the-winner” approach will be utilized, meaning that the nominally better arm (the arm with higher 18-month OS rate, by any amount) will be prioritized for future study.

13.4.2 Secondary endpoints

13.4.2.1 Event-free survival

The distribution of event-free survival will be estimated using the method of Kaplan-Meier in each arm. Event-free survival will be compared between treatment groups using the log-rank test. The correlation between pCR status and event-free survival time will be assessed by Cox model with landmark approach.

13.4.2.2 R0 resection rate

R0 resection rate and confidence intervals for the R0 resection rate will be calculated according to the approach of Duffy and Santner. Chi-square test (or Fisher’s exact test if the data in contingency table is sparse) will be used to compare R0 resection rate between treatment arms. Sensitivity analysis will be conducted among patients in cohort 1) and cohort 2). The association between R0 resection rate and OS/PFS will be assessed by log-rank test and Cox model.

13.4.2.3 Pathologic complete response (pCR) rate

pCR rate and confidence intervals for the pCR rate will be calculated according to the approach of Duffy and Santner. Chi-square test (or Fisher’s exact test if the data in contingency table is sparse) will be used to compare pCR resection rate between two treatment arms. Sensitivity analysis will be conducted among patients in cohort 1) and cohort 2). The association between pCR rate and OS/PFS will be assessed by log-rank test and Cox model.

13.4.2.4 Adverse events (AE)

For CTCAE data, the frequency tables will be reviewed to determine the patterns. The overall adverse event rates will be compared between treatment groups using Chi-square test (or Fisher’s exact test if the data in contingency table is sparse).

PRO-CTCAE data will, at minimum, be analyzed similarly to CTCAE data. Reasons for missed PRO-CTCAE assessments will be collected and we will describe the extent of missing data as well as its patterns and causes. The initial analysis of each PRO-CTCAE item will use all available scores in an analysis which mirrors the approach used for the CTCAE data. Supplemental analysis will use model-based multiple imputation incorporating baseline patient characteristics and physician-rated performance status (which is collected at each cycle). CTCAE data may be incorporated as axillary data into multiple imputation models for AEs which are captured by both PRO-CTCAE and CTCAE. Results from supplemental analysis will be descriptively compared to the results of the initial analysis to assess the robustness of results to missing data. Since a preferred or optimal statistical methodology for PRO-CTCAE data is yet to be determined, additional analyses of PRO-CTCAE data beyond those specified above may be undertaken based on the current state of the science at time of data maturity for this study.

## 13.5 Safety Monitoring

The stopping rule specified below is based on the knowledge available at study development. We note that the Adverse Event Stopping Rule may be adjusted in the event of either (1) the study re-opening to accrual or (2) at any time during the conduct of the trial and in consideration of newly acquired information regarding the adverse event profile of the treatment(s) under investigation. The study team may choose to suspend accrual because of unexpected adverse event profiles that have not crossed the specified rule below.

Accrual will be temporarily suspended to this study if at any time in either arm we observe the following:

- The rate of on-study deaths during treatment, or within the first 90 days following off-protocol treatment, exceeds 2 or more in the first 10 patients or, after 10 patients enrolled, 20% or more of all treated patients. The rate will be assess for each arm separately.

Accrual will also be temporarily suspended to this study if prior to surgery we observe events considered at least possibly related to study treatment (i.e., an adverse event with attribute specified as “possible”, “probable”, or “definite”), in each treatment arm separately, that satisfy the following:

- If 4 or more patients in the first 20 treated patients (or 20% of all patients after 20 are accrued) experience a grade 4 or higher non-hematologic adverse event.

The principal investigator and the study statistician will review the study at least twice a year to identify accrual, adverse event/safety, and any endpoint problems that might be developing. The Alliance Data Safety Monitoring Board (DSMB) is responsible for reviewing accrual and safety data for this trial at least twice a year, based on reports provided by the study statisticians.

## 13.6 Descriptive Factors

- Age: < 55yo versus > 55yo
- Gender
- Albumin < LLN: yes/no

## 13.7 Inclusion of Women and Minorities

This study will be available to all eligible patients, regardless of race, gender, or ethnic origin. There is no information currently available regarding differential effects of this regimen in subsets defined by race, gender, or ethnicity, and there is no reason to expect such differences to exist. Therefore, although the planned analysis will, as always, look for differences in treatment effect based on racial and gender groupings, the sample size is not increased in order to provide additional power for subset analyses.

| **Accrual Targets** | | | | | |
| --- | --- | --- | --- | --- | --- |
| **Racial Categories** | **Ethnic Categories** | | | | **Total** |
|  | **Not Hispanic or Latino** | | **Hispanic or Latino** | |  |
|  | **Female** | **Male** | **Female** | **Male** |  |
| American Indian/ Alaska Native | 2 | 2 | 0 | 0 | 4 |
| Asian | 3 | 7 | 0 | 0 | 10 |
| Native Hawaiian or Other Pacific Islander | 2 | 10 | 0 | 0 | 12 |
| Black or African American | 6 | 1 | 0 | 1 | 8 |
| White | 41 | 53 | 3 | 3 | 100 |
| More Than One Race | 0 | 0 | 0 | 0 | 0 |
| **Total** | 54 | 73 | 3 | 4 | 134 |

| **Ethnic Categories:** | **Hispanic or Latino –** a person of Cuban, Mexican, Puerto Rican, South or Central American, or other Spanish culture or origin, regardless of race. The term “Spanish origin” can also be used in addition to “Hispanic or Latino.”  **Not Hispanic or Latino** |
| --- | --- |
| **Racial Categories:** | **American Indian or Alaskan Native –** a person having origins in any of the original peoples of North, Central, or South America, and who maintains tribal affiliations or community attachment.  **Asian –** a person having origins in any of the original peoples of the Far East, Southeast Asia, or the Indian subcontinent including, for example, Cambodia, China, India, Japan, Korea, Malaysia, Pakistan, the Philippine Islands, Thailand, and Vietnam. (Note: Individuals from the Philippine Islands have been recorded as Pacific Islanders in previous data collection strategies.)  **Black or African American –** a person having origins in any of the black racial groups of Africa. Terms such as “Haitian” or “Negro” can be used in addition to “Black or African American.”  **Native Hawaiian or other Pacific Islander –** a person having origins in any of the original peoples of Hawaii, Guam, Samoa, or other Pacific Islands.  **White –** a person having origins in any of the original peoples of Europe, the Middle East, or North Africa. |

# 14.0 Correlative and Companion Studies

There will be two substudies and all patients are encouraged to participate.

14.1 Pharmacogenetic Studies in Alliance A021501: A021501-PP1

14.1.1 Background

Pharmacogenetics examines inter-individual genetic variability that influences the course of drug action so that medication regimens may be optimized to maximize response, while minimizing drug-induced toxicity. The ability to predict prognosis and efficacy, as well as mitigate adverse drug reactions in oncology is of paramount importance because a majority of chemotherapeutic agents are dosed to their maximally tolerated dose, and possess a narrow therapeutic index between efficacy and toxicity. There is currently a dearth of clinically relevant prognostic and predictive pharmacogenetic markers derived from germline variants that have been translated for incorporation into clinical practice. There is an opportunity to better understand the relationship between germline variants, efficacy, prognosis, and toxicity so that more of these pharmacogenetic markers may be utilized clinically to individualize therapies for cancer patients [40].

This could be particularly helpful in the setting of pancreatic cancer, for example, a disease that is rarely curable at the time of diagnosis and that is almost always associated with survival of little more than a year.

Currently, no biomarkers are available to predict response in patients with this disease, and several targeted therapies have failed to confer a survival advantage. Multidisciplinary efforts have focused on improving tools for early diagnosis and cataloguing the architecture of somatic mutations. In conjunction with these efforts, the interrogation of the germline genome of patients aims at characterizing the constitutive basis of biological systems of fundamental importance for tumor biology, like angiogenesis, inflammation, immunity, and others.

A previous genome-wide association study (GWAS) in 294 pancreatic cancer patients treated with gemcitabine in CALGB 80303 identified several new candidate genes for overall survival (OS) [41, 42]. To discover and validate novel genes and pathways of importance in the biology of pancreatic cancer and the pharmacology of its treatment, we set out to use an external cohort of pancreatic cancer patients from the Mayo Clinic where GWAS information for OS had already been obtained [43].

In the Mayo Clinic patients, three SNPs located in genes (*VDR*, *CMYA5*, *CAMK4*) had an effect on OS concordant with that observed in the CALGB 80303 patients (p<0.05). Consistent with a link between the vitamin D receptor gene (VDR) and pancreatic cancer, a common SNP in VDR (rs2853564, A>G) was associated with OS in patients from both the Mayo Clinic (HR 0.81, 95% CI 0.70-0.94, p=0.0059) and CALGB 80303 (0.74, 0.63-0.87, p=0.0002). Presence of high pre-treatment levels of endogenous vitamin D interacted with rs2853564 to confer improved OS (p<0.01). rs2853564 increased transcriptional activity in luciferase assays and altered binding of the IRF4 transcription factor to VDR. Functional genetic variation in VDR affects the survival of pancreatic cancer patients. This effect is potentiated by an interaction with vitamin D levels.

The prognostic value of the rs2853564 *VDR* variant has never been tested in the setting of borderline resectable disease. In addition, a comprehensive survey of the germline DNA variation of patients might inform the discovery of novel candidate genes and pathways that relate to the pharmacology of treatments and the biology of disease.

14.1.2 Objectives

Primary objective

To test the effect of the rs2853564 *VDR* variant on the 18 months OS rate using both arms combined. We hypothesize that patients with the GG genotype have worse survival than patients with either the AA or AG genotype.

Secondary objectives

1. To test an interaction between the rs2853564 *VDR* variant and treatment arm on the 18 months OS rate. We hypothesize that the effect of the rs2853564 *VDR* variant on survival might differ between the two arms.

**Statistical methods**: Logistic regression will be used to test this interaction. The response variable will be an indicator for survival at 18 months. The predictor variables will include clinical variables, an indicator for rs2853564 VDR variant [GG vs (AA or AG)] and an indicator for treatment arm, and the interaction between variant and treatment arm.

1. To discover novel candidate genes associated with the 18 months OS rate and severe toxicity of chemotherapy. We hypothesize that the genotyping a large series of common germline variants through a genome wide approach will allow the identification of novel gene markers of survival and severe toxicity.
2. To run association analyses with the many molecular markers identified in the correlative science section of this protocol. We hypothesize that the information on the germline variation of patients will associate with molecular markers that relate to constitutional systems like inflammation, immunity, angiogenesis, stromal functions, and others.

**Statistical methods:** Penalized regression methods such as LASSO or elastic net will be utilized to determine combinations of genes that may predict the outcomes. Clinical covariates will be forced into the models. Cross validation will be used in the model building to reduce over-fitting. The logistic framework will be used for dichotomous variables, and a Gaussian or time-to-event framework for continuous or survival outcomes, respectively. We will stay abreast of the literature for modeling strategies that are appropriate for such modeling.

14.1.3 Methods

The germline DNA of patients who have consented to these analyses will be collected from peripheral blood as described in [Section 6.2](#Sec6p2).

Genotyping of the rs2853564 *VDR* variant will be conducted by TaqMan assay (Applied Biosystems, Foster City, CA) per the manufacturer’s instructions using a CFX384 Real-Time System (Bio-Rad, Hercules, CA) and performed in the laboratory of Dr. Innocenti at the University of North Carolina at Chapel Hill. Sanger-based DNA sequencing (Mammalian Genotyping Core at the University of North Carolina at Chapel Hill) will be used to validate representative samples and determine thresholds for allelic discrimination. The allele frequency will be compared with those previously reported from the HapMap and 1,000 Genomes projects. Deviation from Hardy-Weinberg equilibrium (p<0.01 for significance) will be tested to evaluate genotyping accuracy.

Genome-wide genotyping will be performed by the OmniExpress Exome chip, which enables whole-genome genotyping of over 700,000 single nucleotide polymorphism (SNP) loci. This platform gives an outstanding breadth of coverage over the genome for the planned association studies. It will be performed at the Mammalian Genotyping Core at the University of North Carolina at Chapel Hill.

Exome and whole sequencing could be also performed from germline and somatic DNA to detect associations between outcome and rare variants.

Logistic regression will be used to access the relationship between rs2853564 VDR variant and 18 month OS rate. The treatment assignment and the interaction term between rs2853564 VDR variant and treatment assignment will entered the model in order to examine whether the effect of the rs2853564 VDR variant differ between 2 arms.

Fisher’s Exact test will be used to assess the association of the GG genotype with worse 18 month OS. With a total sample size of N=125 in both arms, and 85% expected rate of consent to this study, we expect a sample size of 106 for this aim. We assume 63% 18 month OS rate (the alternative hypothesis in the primary aim of this trial) and 16% prevalence of the GG variant. We expect n=17 GG variant patients. Under these assumptions, a Fisher’s Exact test has 82% power to detect a difference of 44% versus 82% (i.e., a delta of 38%) 18 month OS rates between the groups defined by the GG variant. Nonparametric methods such as recursive partitioning for classification, regression and survival trees (RPART) will be used to explore a possible predictive value of the GG genotype (secondary objective 1), and prognostic value of common variants (secondary objective 2) [44]. In secondary objective 4, generalized linear model methods and penalized regression together with cross validation will be used to evaluate the association between germline variation and molecular markers of inflammation, immunity, angiogenesis, and stromal functions in an exploratory fashion.

Similar approaches to *Quality Control and Normalization of Expression Data, Differential expression, Model Building, and Internal Model Validation* will be employed. We will stay abreast of the literature for methods that are appropriate for data analysis. A detailed analysis plan will be formulated prior to actual data analysis.

## 14.2 Imaging Science Studies in Alliance A021501: Alliance A021501-IM1

We propose three imaging correlates for this study. The primary objective of these correlative studies is to prospectively validate prognostic biomarkers derived from imaging. We have reviewed how previous clinical trials in this disease could have benefited from stratification and proper selection [45] [46]. By validating the imaging-based biomarkers in this trial, we can develop a better understanding of the results of this trial and design the next generation of trials with these biomarkers in mind. We have previously shown that standard imaging can correlate with drug delivery in PDAC [36]. Our group has pilot data to suggest that two imaging-based properties independently predict overall survival (OS) outcomes of patients. These measurements are the degree of enhancement in the tumor relative to the normal pancreas (called normalized area under the enhancement curve, NAUC) and a measurement of the morphology of the tumor (called the delta measurement). Our pilot data also indicate that changes in NAUC associate with local control of the tumor, and we hypothesize this observation is due to the pathological response in the tumor after neoadjuvant therapy. With a planned accrual of 134 patients in this Alliance trial, we will be well powered to prospectively validate our pilot data.

14.2.1 Primary Objectives

To evaluate risk classification previously developed by Koay et al [36] using NAUC.

To access prognostic value of NAUC ration defined as post-neoadjuvant NAUC divided by pre-neoadjuvant therapy NAUC.

To evaluate risk classification previously developed by Koay et al using delta measure.

14.2.2 NAUC and OS

In a pilot study of 110 patients who underwent neoadjuvant gemcitabine-based chemoradiation for resectable pancreatic cancer, NAUC from pre-therapy CT scans associated with OS as a survival variable. Exploratory analysis (Koay et al, JCI 2014) identified a cutoff of 0.6 for NAUC, differentiating patients into two prognostic groups: high NAUC (20% of patients) and low NAUC (80% of patients) [36]. The hazard ratio for this cutoff was 0.46 [0.24, 0.79], favoring patients with a higher NAUC.

To validate the cutoff, NAUC will be calculated for each evaluable patient based on pre-therapy CT scans. Patients will then be categorized into two groups based on NAUC value (> 0.6 versus ≤ 0.6). Proportional hazard model will be used to examine the associate between NAUC grouping (high versus low) and overall survival adjusting for treatment arm to assess the prognostic value of NAUC.

Assuming 20% of patients will have NAUC > 0.6, we will have 80% power to detect a difference in overall survival at a one-sided 5% significance level with a hazard ratio of 0.47. The power curve for different percentages of patients who have NACU > 0.6 is shown in Figure S4 below. This power analysis assumes an accrual period of 31 months, a minimum follow up of 20 months, an accrual rate of 4 patients per month, and median survival in the worst group of 18 months.


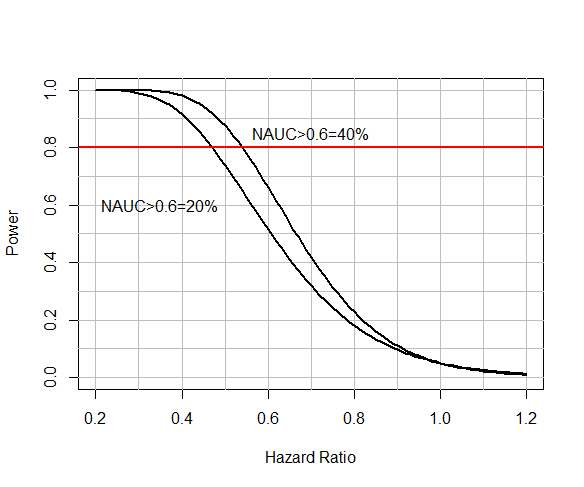


Figure S4. Power curve for different percentages of patients who have NAUC > 0.6.

NAUC will be calculated for each evaluable patient based on pre-therapy CT scans. Patients then will be categorized into 2 groups based on NAUC value (> 0.6 vs. ≤ 0.6). Proportional hazard model will be used to examine the association between NAUC grouping and overall survival

If NAUC > 0.6 cutoff is not validated, we will explore an additional cut-point to better differentiate patients to two risk groups. The methods used for exploratory analysis including, but not limited to, 1) graphical approaches based on Martingale Residual plot [47], 2) Mazumdar and Classman cut-off identification strategy [48] (minimum p-value and maximum HR approach), and 3) Contal and O’Quidley method (changepoint method) [49]. A detailed analysis plan will be formulated prior to actual data analysis.

14.2.3 Changes in NAUC and pathological response.

As a second correlate for the mass transport properties, the change in NAUC during therapy may also provide important information. As an exploratory exercise, NAUC will be calculated for each evaluable patient based on pre-therapy and pre-surgery CT scans. A NAUC ratio of pre-surgery to pre-therapy NAUC will then be calculated for each patient. Proportional hazard model will be used to examine the association between NAUC ratio grouping, progression free survival and overall survival

Additionally, we propose analyzing changes in the NAUC (comparing post-neoadjuvant therapy and pre-neoadjuvant therapy) as a prognostic biomarker of the pathological response to neoadjuvant therapy. Our pilot data indicate that patients who have a decrease in NAUC after neoadjuvant therapy (NAUC ratio of post- to pre- NAUC less than 1) have significantly better local control than those who have a NAUC ratio above 1, with a hazard ratio of 3.01 [1.45, 6.25]). We hypothesize this difference in the groups is based on response at the pathological level. As an exploratory study, we will score the pathological response in patients who undergo resection on this Alliance trial and measure NAUC on post- and pre-therapy CT scans, similar to our previously published methods.[36] This estimate was based on a conservative estimate of the hazard ratio from our pilot study (2.2) and the same assumptions as (A). The model will include a treatment arm indicator. In addition, the predictive value will be assessed by testing the interaction between NAUC and treatment arm.

If our preliminary data reflect the differences that we can expect, then we estimate we will need a minimum of 62 patients to detect a difference in the NAUC ratio between those who have an excellent response to therapy from all others. This estimate was based on a conservative estimate of the hazard ratio from our pilot study (2.2) and the same assumptions as (A).

NAUC ratio of pre-surgery to pre-therapy NAUC will be calculated for each patient. Proportional hazard model will be used to examine the association between NAUC ratio (as a continuous variable) and overall survival adjusting for treatment arm to assess the prognostic value of NAUC ratio. Optimal cut point of NAUC ratio will be explored to dichotomize patients into two risk groups.

Assuming 60% of evaluable patients (N=74) undergo resection, there will be are 41 events. The power curve is a function of hazard ratio and the variance of the NAUC ratio (Figure S5).

Figure S5. Power curve
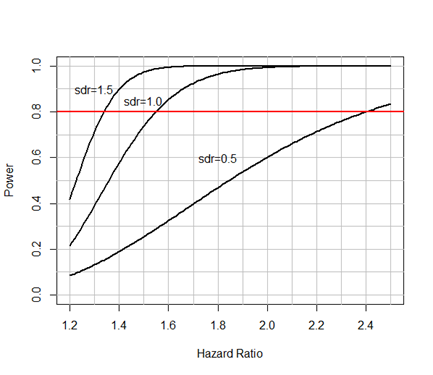
 as a function of hazard ratio and the variance of the NAUC ratio.

We will have 80% power to detect a difference in overall survival at a two-sided 5% significance level with a hazard ratio of 1.55 if the variance of NAUC ratio is one. Similarly, we can detect a difference of 1.32 and 2.4 if the variance of NAUC ratio is 1.5 and 0.5, respectively. This power analysis assumes a minimal follow-up of 20 months and accrual rate of 4 patients per month. A detailed analysis plan will be formulated prior to actual data analysis.

14.2.4 Tumor morphological measurements and OS.

The final imaging correlate that we will perform will also involve analysis of the pre-therapy CT images. We will obtain a measurement of the tumor-pancreas interface (the delta measurement). In our pilot study, we identified a cutoff for this delta measurement from the CT scans by quantifying the amount of stroma in treatment naïve specimens of 12 patients who underwent upfront resection. Patients with a delta measure ≥ 40 Hounsfield Units (HU) had significantly less stroma than patients with a delta measure < 40 HU. This cutoff was validated in a separate cohort of 33 patients who underwent upfront resection. This defined high and low delta classes.

We have found this delta classification to differentiate 156 patients who received neoadjuvant therapy into two distinct prognostic groups, where patients with a low delta tumor have significantly better prognosis than those with a high delta tumor (difference in OS, HR 0.58 [0.40, 0.81]), due to differences in the tumor biology between tumors that have a high delta (> 40 onuce/unit) compared to those with a low delta (≤ 40 ounce/unit), as defined by our quantitative image analysis.

We will validate the delta classification as a prognostic biomarker to OS. The delta classification will be calculated for each evaluable patient based on pre-therapy CT scans. Proportional hazard models will be used to examine the association between delta classification (high or low) and overall survival adjusting for treatment arm.

Assuming 40% of evaluable patients will have high delta, we will have 80% power to detect a difference in OS at a two sided 5% significance level with a hazard ratio of 0.51. We will analyze this imaging marker independent of randomization on the study. This power analysis assumes an accrual period of 31 months, a minimum follow-up of 20 months, an accrual rate of 4 patients per month, and a median survival in the worst group of 18 months.

If delta measure > 40 HU is not validated, we will explore an additional cut-point to better differentiate patients into two risk groups. The methods used for exploratory analysis include, but are not limited to, the following: 1) graphical approaches based on Martingale Residual plot [47] 2) Mazumdar and Classman cut-off identification strategy [48] (minimum p-value and maximum HR approach), and 3) Contal and O’Quidley method (changepoint method) [49]. A detailed analysis plan will be formulated prior to actual data analysis.

The delta measurement will be calculated for each evaluable patients based on pre-therapy CT scans. We will examine measurement and search for a cut-point which will separate patients into two distinct prognostic groups. Proportional hazard model will be used to examine the association between delta measurement grouping and overall survival.

The delta measurement will be calculated for each evaluable patient based on pre-therapy CT scans. Proportional hazard models will be used to examine the association between delta measurement grouping and overall survival adjusting for treatment arm. In addition, the predictive value will be assessed by testing the interaction between the delta variable and treatment arm. We will examine the delta measurement and outcome, and evaluate the possibility of search for a cut-point which may separate patients into two distinct prognostic groups.

# 15.0 General Regulatory Considerations and Credentialing

## 15.1 Institutional Credentialing

15.1.1 IROC Ohio institutional requirements

Prior to the enrollment of patients, institutions that have not previously been credentialed for any other Alliance trials must be credentialed to participate in the trial by the Imaging Core Laboratory at IROC Ohio. For institutions that have previously been credentialed by the Imaging Core Laboratory at IROC Ohio to participate in imaging studies, the ICL will provide a brief A021501 protocol refresher prior to patient enrollments. Institutions should contact the Alliance ICL directly to complete credentialing or a refresher for A021501.

Contact information for IROC Ohio can be found under protocol contacts near the title page.

15.1.2 Radiation Therapy Requirements

Institutions that have been previously credentialed for participation in RTOG 1112 need only update their Facility Questionnaire as indicated in the table below. All other institutions must be credentialed for the modality they plan to use prior to delivery of radiation therapy on any protocol patient. Credentialing requirements are listed in the table below. Use of IMRT is required for those treating with SBRT. Use of proton therapy is not allowed.

Web Link for Credentialing Procedures and Instructions: http://irochouston.mdanderson.org

|  | **Treatment Modality** | |  |
| --- | --- | --- | --- |
| **RT Credentialing Requirements** | **HIGRT** | **SBRT** | **Key Information** |
| Facility Questionnaire | X | X | The IROC Houston electronic facility questionnaire (FQ) should be completed or updated with the most recent information about your institution. To access this FQ, email irochouston@mdanderson.org to receive your FQ link. |
| Phantom Irradiation |  | X | An IMRT liver phantom w/motion management study provided by the IROC Houston QA Center must be successfully completed. Instructions for requesting and irradiating the phantom may be found on the IROC Houston website (http://irochouston.mdanderson.org).  Tomotherapy and Cyberknife treatment delivery modalities must be credentialed individually. |
|  | w/IMRT |  | Successful completion of a phantom study is required. Prior irradiation of the head and neck phantom will be accepted. |
| IGRT |  | X | Instructions for IGRT credentialing may be found on the IROC Houston website (http://irochouston.mdanderson.org). |
| Credentialing Status Inquiry Form | X | X | To determine if your institution has completed the requirements above, please complete a “Credentialing Status Inquiry Form” found under Credentialing on the IROC Houston QA Center website (<http://irochouston.mdanderson.org>). |
| Institution |  |  | Institutions will be credentialed for the treatment modality that they intend to use on all patients. Institutions credentialed for use of SBRT may treat with either SBRT or HIGRT. IROC Houston QA Center will notify the institution and Alliance Headquarters that all desired credentialing requirements have been met. |

# 16.0 References

1. Katz, M.H., et al., *Borderline resectable pancreatic cancer: the importance of this emerging stage of disease.* J Am Coll Surg, 2008. **206**(5): p. 833-46; discussion 846-8.

2. Ishikawa, O., et al., *Preoperative indications for extended pancreatectomy for locally advanced pancreas cancer involving the portal vein.* Ann Surg, 1992. **215**(3): p. 231-6.

3. Nakao, A., et al., *Correlation between radiographic classification and pathological grade of portal vein wall invasion in pancreatic head cancer.* Ann Surg, 2012. **255**(1): p. 103-8.

4. Abrams, R.A., et al., *Combined modality treatment of resectable and borderline resectable pancreas cancer: expert consensus statement.* Ann Surg Oncol, 2009. **16**(7): p. 1751-6.

5. Callery, M.P., et al., *Pretreatment assessment of resectable and borderline resectable pancreatic cancer: expert consensus statement.* Ann Surg Oncol, 2009. **16**(7): p. 1727-33.

6. Oettle, H., et al., *Adjuvant chemotherapy with gemcitabine vs observation in patients undergoing curative-intent resection of pancreatic cancer: a randomized controlled trial.* JAMA, 2007. **297**(3): p. 267-77.

7. Neoptolemos, J.P., et al., *A randomized trial of chemoradiotherapy and chemotherapy after resection of pancreatic cancer.* N Engl J Med, 2004. **350**(12): p. 1200-10.

8. Abrams, R.A. *RTOG 0848: A Phase IIR and A Phase III Trial Evaluating Both Erlotinib (Ph IIR) And Chemoradiation (Ph III) As Adjuvant Treatment For Patients With Resected Head Of Pancreas Adenocarcinoma*. 2014 [cited 2015; Available from: <https://www.rtog.org/ClinicalTrials/ProtocolTable/StudyDetails.aspx?study=0848>.

9. Hammel, P., et al. *Comparison of chemoradiotherapy (CRT) and chemotherapy (CT) in patients with a locally advanced pancreatic cancer (LAPC) controlled after 4 months of gemcitabine with or without erlotinib: Final results of the international phase III LAP 07 study*. in *ASCO Annual Meeting Proceedings*. 2013.

10. Katz, M.H., et al., *Response of borderline resectable pancreatic cancer to neoadjuvant therapy is not reflected by radiographic indicators.* Cancer, 2012. **118**(23): p. 5749-56.

11. Conroy, T., et al., *FOLFIRINOX versus gemcitabine for metastatic pancreatic cancer.* N Engl J Med, 2011. **364**(19): p. 1817-25.

12. Von Hoff, D.D., et al., *Increased survival in pancreatic cancer with nab-paclitaxel plus gemcitabine.* N Engl J Med, 2013. **369**(18): p. 1691-703.

13. Herman, J.M., et al., *Phase 2 multi-institutional trial evaluating gemcitabine and stereotactic body radiotherapy for patients with locally advanced unresectable pancreatic adenocarcinoma.* Cancer, 2015. **121**(7): p. 1128-37.

14. Mellon, E.A., et al., *Long-term outcomes of induction chemotherapy and neoadjuvant stereotactic body radiotherapy for borderline resectable and locally advanced pancreatic adenocarcinoma.* Acta Oncologica, 2015(0): p. 1-7.

15. Katz, M.H., et al., *Borderline resectable pancreatic cancer: need for standardization and methods for optimal clinical trial design.* Ann Surg Oncol, 2013. **20**(8): p. 2787-95.

16. Katz, M.H., et al. *Preoperative modified FOLFIRINOX (mFOLFIRINOX) followed by chemoradiation (CRT) for borderline resectable (BLR) pancreatic cancer (PDAC): Initial results from Alliance Trial A021101*. in *ASCO Annual Meeting Proceedings*. 2015.

17. Katz, M.G., et al., *Preoperative modified folfirinox treatment followed by capecitabine-based chemoradiation for borderline resectable pancreatic cancer: Alliance for clinical trials in oncology trial a021101.* JAMA Surgery, 2016. **151**(8): p. e161137.

18. Heinrich, S., et al., *Prospective phase II trial of neoadjuvant chemotherapy with gemcitabine and cisplatin for resectable adenocarcinoma of the pancreatic head.* J Clin Oncol, 2008. **26**(15): p. 2526-31.

19. O'Reilly, E.M., et al., *A single-arm, nonrandomized phase II trial of neoadjuvant gemcitabine and oxaliplatin in patients with resectable pancreas adenocarcinoma.* Ann Surg, 2014. **260**(1): p. 142-8.

20. Rose, J.B., et al., *Extended neoadjuvant chemotherapy for borderline resectable pancreatic cancer demonstrates promising postoperative outcomes and survival.* Ann Surg Oncol, 2014. **21**(5): p. 1530-7.

21. Loehrer, P.J., Sr., et al., *Gemcitabine alone versus gemcitabine plus radiotherapy in patients with locally advanced pancreatic cancer: an Eastern Cooperative Oncology Group trial.* J Clin Oncol, 2011. **29**(31): p. 4105-12.

22. Huguet, F., et al., *Impact of chemoradiotherapy after disease control with chemotherapy in locally advanced pancreatic adenocarcinoma in GERCOR phase II and III studies.* J Clin Oncol, 2007. **25**(3): p. 326-31.

23. Chauffert, B., et al., *Phase III trial comparing intensive induction chemoradiotherapy (60 Gy, infusional 5-FU and intermittent cisplatin) followed by maintenance gemcitabine with gemcitabine alone for locally advanced unresectable pancreatic cancer. Definitive results of the 2000-01 FFCD/SFRO study.* Ann Oncol, 2008. **19**(9): p. 1592-9.

24. Chang, D.T., et al., *Stereotactic radiotherapy for unresectable adenocarcinoma of the pancreas.* Cancer, 2009. **115**(3): p. 665-72.

25. Chang, B.K. and R.D. Timmerman, *Stereotactic body radiation therapy: a comprehensive review.* Am J Clin Oncol, 2007. **30**(6): p. 637-44.

26. Timmerman, R., et al., *Optimizing dose and fractionation for stereotactic body radiation therapy. Normal tissue and tumor control effects with large dose per fraction.* Front Radiat Ther Oncol, 2007. **40**: p. 352-65.

27. Moningi, S., et al., *The Role of Stereotactic Body Radiation Therapy for Pancreatic Cancer: A Single-Institution Experience.* Ann Surg Oncol, 2015. **22**(7): p. 2352-8.

28. Pollom, E.L., et al., *Single-versus multifraction stereotactic body radiation therapy for pancreatic adenocarcinoma: outcomes and toxicity.* International Journal of Radiation Oncology* Biology* Physics, 2014. **90**(4): p. 918-925.

29. Varadhachary, G.R., et al., *Preoperative gemcitabine and cisplatin followed by gemcitabine-based chemoradiation for resectable adenocarcinoma of the pancreatic head.* J Clin Oncol, 2008. **26**(21): p. 3487-95.

30. Hong, T.S., et al., *A phase 1/2 and biomarker study of preoperative short course chemoradiation with proton beam therapy and capecitabine followed by early surgery for resectable pancreatic ductal adenocarcinoma.* International Journal of Radiation Oncology* Biology* Physics, 2014. **89**(4): p. 830-838.

31. Chuong, M.D., et al., *Stereotactic body radiation therapy for locally advanced and borderline resectable pancreatic cancer is effective and well tolerated.* Int J Radiat Oncol Biol Phys, 2013. **86**(3): p. 516-22.

32. Koong, A.C., et al., *Phase I study of stereotactic radiosurgery in patients with locally advanced pancreatic cancer.* Int J Radiat Oncol Biol Phys, 2004. **58**(4): p. 1017-21.

33. Koong, A.C., et al., *Phase II study to assess the efficacy of conventionally fractionated radiotherapy followed by a stereotactic radiosurgery boost in patients with locally advanced pancreatic cancer.* Int J Radiat Oncol Biol Phys, 2005. **63**(2): p. 320-3.

34. Schellenberg, D., et al., *Gemcitabine chemotherapy and single-fraction stereotactic body radiotherapy for locally advanced pancreatic cancer.* Int J Radiat Oncol Biol Phys, 2008. **72**(3): p. 678-86.

35. Schellenberg, D., et al., *Single-fraction stereotactic body radiation therapy and sequential gemcitabine for the treatment of locally advanced pancreatic cancer.* Int J Radiat Oncol Biol Phys, 2011. **81**(1): p. 181-8.

36. Koay, E.J., et al, *Transport properties of pancreatic cancer describe gemcitabine delivery and response.* Journal of Clinical Investigations, 2014. **124**(4): p. 1525-36.

37. Nelson, H., American College of Surgeons, and Alliance for Clinical Trials in Oncology, *Operative standards for cancer surgery*. xxxiii, 332 pages.

38. Eisenhauer, E., et al., *New response evaluation criteria in solid tumours: revised RECIST guideline (version 1.1).* European journal of cancer, 2009. **45**(2): p. 228-247.

39. Duffy, D.E. and T.J. Santner, *Confidence Intervals for a Binomial Parameter Based on Multistage Tests.* Biometrics, 1987. **43**(1): p. 81-93.

40. Crona, D. and F. Innocenti, *Can knowledge of germline markers of toxicity optimize dosing and efficacy of cancer therapy?* Biomarkers in medicine, 2012. **6**(3): p. 349-362.

41. Innocenti, F., et al., *A genome-wide association study of overall survival in pancreatic cancer patients treated with gemcitabine in CALGB 80303.* Clinical Cancer Research, 2012. **18**(2): p. 577-584.

42. Kindler, H.L., et al., *Gemcitabine plus bevacizumab compared with gemcitabine plus placebo in patients with advanced pancreatic cancer: phase III trial of the Cancer and Leukemia Group B (CALGB 80303).* Journal of Clinical Oncology, 2010. **28**(22): p. 3617-3622.

43. Petersen, G.M., et al., *A genome-wide association study identifies pancreatic cancer susceptibility loci on chromosomes 13q22. 1, 1q32. 1 and 5p15. 33.* Nature genetics, 2010. **42**(3): p. 224-228.

44. Therneau, T., and Atkinson, EJ, *An introduction to recursive partitioning using the RPART routines.* Section of Biostatistics, Mayo Clinic, Technical Report Series, 1997. **61**.

45. Koay, E.J., et al, *Toward stratification of patients with pancreatic cancer: past lessons from traditional approaches and future applications with physical biomarkers.* Cancer Letters, 2016.

46. Crane, C.H. and C.A. Iacobuzio-Donahue, *Keys to Personalized Care in Pancreatic Oncology.* Journal of Clinical Oncology, 2012. **30**(33): p. 4049-4950.

47. Mandrekar, J., et al, *Cutpoint determination methods in survival analysis using SAS. .* Proceedings of the 28th SAS Users Group International Conference (SUGI) 2003, 2003: p. 261–28.

48. Mazumdar, M. and J.R. Glassman, *Categorizing a prognostic variable: review of methods, code for easy implementation and applications to decision-making about cancer treatments.* Stat Med, 2000. **19**(1): p. 113-32.

49. Contal, C., O'Quigley, J., *An application of changepoint methods in studying the effect of age on survival in breast cancer.* Computational Statistics and Data Analysis,, 1999. **30**: p. 253-70.

# Appendix I: Registration QOL/Mental Well-being/Fatigue and PRO-CTCAE Measures

**Registration QOL/Mental Well-being/Physical Well-being/Fatigue**

At patient registration, this form is to be administered by a nurse/CRA, completed by the patient, and entered into Medidata Rave at the time of registration.

If needed, this appendix can be adapted to use as a source document. A booklet containing this assessment does not exist – please do not order this booklet.

How would you describe:

**1. your overall qualify of life in the past week including today?**

0 1 2 3 4 5 6 7 8 9 10

As bad as As good as

it can be it can be

**2. your overall mental (intellectual) well being in the past week including today?**

0 1 2 3 4 5 6 7 8 9 10

As bad as As good as

it can be it can be

**3. your overall physical well being in the past week including today?**

0 1 2 3 4 5 6 7 8 9 10

As bad as As good as

it can be it can be

**4. your level of fatigue, on average in the past week including today?**

0 1 2 3 4 5 6 7 8 9 10

No Fatigue

fatigue as bad as it can be

**NCI PRO-CTCAE^TM^ Items - English**

**Item Library Version 1.0**

As individuals go through treatment for their cancer they sometimes experience different symptoms and side effects. For each question, please check or mark an in the one box that best describes your experiences over the past 7 days…

| 1. | In the last 7 days, what was the SEVERITY of your DRY MOUTH at its WORST? | | | | |
| --- | --- | --- | --- | --- | --- |
|  | ○ None | ○ Mild | ○ Moderate | ○ Severe | ○ Very severe |

| 2. | In the last 7 days, what was the SEVERITY of your PROBLEMS WITH TASTING FOOD OR DRINK at their WORST? | | | | |
| --- | --- | --- | --- | --- | --- |
|  | ○ None | ○ Mild | ○ Moderate | ○ Severe | ○ Very severe |

| 3. | In the last 7 days, what was the SEVERITY of your DECREASED APPETITE at its WORST? | | | | |
| --- | --- | --- | --- | --- | --- |
|  | ○ None | ○ Mild | ○ Moderate | ○ Severe | ○ Very severe |
|  | In the last 7 days, how much did DECREASED APPETITE INTERFERE with your usual or daily activities? | | | | |
|  | ○ Not at all | ○ A little bit | ○ Somewhat | ○ Quite a bit | ○ Very much |

| 4. | In the last 7 days, how OFTEN did you have NAUSEA? | | | | |
| --- | --- | --- | --- | --- | --- |
|  | ○ Never | ○ Rarely | ○ Occasionally | ○ Frequently | ○ Almost constantly |
|  | In the last 7 days, what was the SEVERITY of your NAUSEA at its WORST? | | | | |
|  | ○ None | ○ Mild | ○ Moderate | ○ Severe | ○ Very severe |

| 5. | In the last 7 days, how OFTEN did you have VOMITING? | | | | |
| --- | --- | --- | --- | --- | --- |
|  | ○ Never | ○ Rarely | ○ Occasionally | ○ Frequently | ○ Almost constantly |
|  | In the last 7 days, what was the SEVERITY of your VOMITING at its WORST? | | | | |
|  | ○ None | ○ Mild | ○ Moderate | ○ Severe | ○ Very severe |

| 6. | In the last 7 days, how OFTEN did you have HEARTBURN? | | | | |
| --- | --- | --- | --- | --- | --- |
|  | ○ Never | ○ Rarely | ○ Occasionally | ○ Frequently | ○ Almost constantly |
|  | In the last 7 days, what was the SEVERITY of your HEARTBURN at its WORST? | | | | |
|  | ○ None | ○ Mild | ○ Moderate | ○ Severe | ○ Very severe |

The PRO-CTCAE™ items and information herein were developed by the NATIONAL CANCER INSTITUTE at the NATIONAL INSTITUTES OF HEALTH, in Bethesda, Maryland, U.S.A. Use of the PRO-CTCAE™ is subject to NCI's Terms of Use.

**NCI PRO-CTCAE^TM^ Items - English**

**Item Library Version 1.0**

| 7. | In the last 7 days, how OFTEN did you have BLOATING OF THE ABDOMEN (BELLY)? | | | | |
| --- | --- | --- | --- | --- | --- |
|  | ○ Never | ○ Rarely | ○ Occasionally | ○ Frequently | ○ Almost constantly |
|  | In the last 7 days, what was the SEVERITY of your BLOATING OF THE ABDOMEN (BELLY) at its WORST? | | | | |
|  | ○ None | ○ Mild | ○ Moderate | ○ Severe | ○ Very severe |

| 8. | In the last 7 days, how OFTEN did you have LOOSE OR WATERY STOOLS (DIARRHEA)? | | | | |
| --- | --- | --- | --- | --- | --- |
|  | ○ Never | ○ Rarely | ○ Occasionally | ○ Frequently | ○ Almost constantly |

| 9. | In the last 7 days, how OFTEN did you have PAIN IN THE ABDOMEN (BELLY AREA)? | | | | |
| --- | --- | --- | --- | --- | --- |
|  | ○ Never | ○ Rarely | ○ Occasionally | ○ Frequently | ○ Almost constantly |
|  | In the last 7 days, what was the SEVERITY of your PAIN IN THE ABDOMEN (BELLY AREA) at its WORST? | | | | |
|  | ○ Never | ○ Mild | ○ Moderate | ○ Severe | ○ Very severe |
|  | In the last 7 days, how much did PAIN IN THE ABDOMEN (BELLY AREA) INTERFERE with your usual or daily activities? | | | | |
|  | ○ Not at all | ○ A little bit | ○ Somewhat | ○ Quite a bit | ○ Very much |

| 10. | In the last 7 days, what was the SEVERITY of your ITCHY SKIN at its WORST? | | | | |
| --- | --- | --- | --- | --- | --- |
|  | ○ None | ○ Mild | ○ Moderate | ○ Severe | ○ Very severe |

| 11. | In the last 7 days, what was the SEVERITY of your NUMBNESS OR TINGLING IN YOUR HANDS OR FEET at its WORST? | | | | |
| --- | --- | --- | --- | --- | --- |
|  | ○ None | ○ Mild | ○ Moderate | ○ Severe | ○ Very severe |
|  | In the last 7 days, how much did NUMBNESS OR TINGLING IN YOUR HANDS OR FEET INTERFERE with your usual or daily activities? | | | | |
|  | ○ Not at all | ○ A little bit | ○ Somewhat | ○ Quite a bit | ○ Very much |

| 12. | In the last 7 days, how OFTEN did you have PAIN? | | | | |
| --- | --- | --- | --- | --- | --- |
|  | ○ Never | ○ Rarely | ○ Occasionally | ○ Frequently | ○ Almost constantly |
|  | In the last 7 days, what was the SEVERITY of your PAIN at its WORST? | | | | |
|  | ○ Never | ○ Mild | ○ Moderate | ○ Severe | ○ Very severe |
|  | In the last 7 days, how much did PAIN INTERFERE with your usual or daily activities? | | | | |
|  | ○ Not at all | ○ A little bit | ○ Somewhat | ○ Quite a bit | ○ Very much |

The PRO-CTCAE™ items and information herein were developed by the NATIONAL CANCER INSTITUTE at the NATIONAL INSTITUTES OF HEALTH, in Bethesda, Maryland, U.S.A. Use of the PRO-CTCAE™ is subject to NCI's Terms of Use.

**NCI PRO-CTCAE^TM^ Items - English**

**Item Library Version 1.0**

| 13. | In the last 7 days, what was the SEVERITY of your FATIGUE, TIREDNESS, OR LACK OF ENERGY at its WORST? | | | | |
| --- | --- | --- | --- | --- | --- |
|  | ○ None | ○ Mild | ○ Moderate | ○ Severe | ○ Very severe |
|  | In the last 7 days, how much did FATIGUE, TIREDNESS, OR LACK OF ENERGY  INTERFERE with your usual or daily activities? | | | | |
|  | ○ Not at all | ○ A little bit | ○ Somewhat | ○ Quite a bit | ○ Very much |

| 14. | In the last 7 days, how OFTEN did you feel ANXIETY? | | | | |
| --- | --- | --- | --- | --- | --- |
|  | ○ Never | ○ Rarely | ○ Occasionally | ○ Frequently | ○ Almost constantly |
|  | In the last 7 days, what was the SEVERITY of your ANXIETY at its WORST? | | | | |
|  | ○ None | ○ Mild | ○ Moderate | ○ Severe | ○ Very severe |
|  | In the last 7 days, how much did ANXIETY INTERFERE with your usual or daily activities? | | | | |
|  | ○ Not at all | ○ A little bit | ○ Somewhat | ○ Quite a bit | ○ Very much |

| 15. | In the last 7 days, how OFTEN did you have SAD OR UNHAPPY FEELINGS? | | | | |
| --- | --- | --- | --- | --- | --- |
|  | ○ Never | ○ Rarely | ○ Occasionally | ○ Frequently | ○ Almost constantly |
|  | In the last 7 days, what was the SEVERITY of your SAD OR UNHAPPY FEELINGS at their WORST? | | | | |
|  | ○ None | ○ Mild | ○ Moderate | ○ Severe | ○ Very severe |
|  | In the last 7 days, how much did SAD OR UNHAPPY FEELINGS INTEFERE with your usual or daily activities? | | | | |
|  | ○ Not at all | ○ A little bit | ○ Somewhat | ○ Quite a bit | ○ Very much |

The PRO-CTCAE™ items and information herein were developed by the NATIONAL CANCER INSTITUTE at the NATIONAL INSTITUTES OF HEALTH, in Bethesda, Maryland, U.S.A. Use of the PRO-CTCAE™ is subject to NCI's Terms of Use.

**NCI PRO-CTCAE^TM^ Items - English**

**Item Library Version 1.0**

| Do you have any other symptoms that you wish to report? | |
| --- | --- |
| ○ Yes | ○ No |

Please list any other symptoms:

| 1. | In the last 7 days, what was the SEVERITY of this symptom at its WORST? | | | | |
| --- | --- | --- | --- | --- | --- |
|  | ○ None | ○ Mild | ○ Moderate | ○ Severe | ○ Very severe |
| 2. | In the last 7 days, what was the SEVERITY of this symptom at its WORST? | | | | |
|  | ○ None | ○ Mild | ○ Moderate | ○ Severe | ○ Very severe |
| 3. | In the last 7 days, what was the SEVERITY of this symptom at its WORST? | | | | |
|  | ○ None | ○ Mild | ○ Moderate | ○ Severe | ○ Very severe |
| 4. | In the last 7 days, what was the SEVERITY of this symptom at its WORST? | | | | |
|  | ○ None | ○ Mild | ○ Moderate | ○ Severe | ○ Very severe |
| 5. | In the last 7 days, what was the SEVERITY of this symptom at its WORST? | | | | |
|  | ○ None | ○ Mild | ○ Moderate | ○ Severe | ○ Very severe |

The PRO-CTCAE™ items and information herein were developed by the NATIONAL CANCER INSTITUTE at the NATIONAL INSTITUTES OF HEALTH, in Bethesda, Maryland, U.S.A. Use of the PRO-CTCAE™ is subject to NCI's Terms of Use.

**NCI PRO-CTCAETM Items - Spanish**

**Item Library Version 1.0**

Los pacientes que reciben tratamiento para el cáncer a menudo presentan ciertos

síntomas y efectos secundarios. Para cada pregunta, haga una marca o escriba una en

la casilla que mejor describe sus experiencias en los últimos siete días…

| 1. | En los últimos 7 días, ¿cuál fue la INTENSIDAD de la SEQUEDAD EN LA BOCA en su PEOR momento? | | | | |
| --- | --- | --- | --- | --- | --- |
|  | ○ Ninguna | ○ Leve | ○ Moderada | ○ Intensa | ○ Muy intensa |

| 2. | En los últimos 7 días, ¿cuál fue la INTENSIDAD de los PROBLEMAS PARA NOTAR EL SABOR DE LAS COMIDAS O LAS BEBIDAS en su PEOR momento? | | | | |
| --- | --- | --- | --- | --- | --- |
|  | ○ Ninguna | ○ Leve | ○ Moderada | ○ Intensa | ○ Muy intensa |

| 3. | En los últimos 7 días, ¿cuál fue la INTENSIDAD de la DISMINUCIÓN DEL APETITO en su PEOR momento? | | | | |
| --- | --- | --- | --- | --- | --- |
|  | ○ Ninguna | ○ Leve | ○ Moderada | ○ Intensa | ○ Muy intensa |
|  | En los últimos 7 días, ¿cuánto INTERFIRIÓ la DISMINUCIÓN DEL APETITO en sus actividades habituales o diarias? | | | | |
|  | ○ Nada | ○ Un poco | ○ Algo | ○ Muco | ○ Muchísimo |

| 4. | En los últimos 7 días, ¿con qué FRECUENCIA tuvo NÁUSEAS? | | | | |
| --- | --- | --- | --- | --- | --- |
|  | ○ Nunca | ○ Rara vez | ○ A veces | ○ A menudo | ○ Casi siempre |
|  | En los últimos 7 días, ¿cuál fue la INTENSIDAD de las NÁUSEAS en su PEOR momento? | | | | |
|  | ○ Ninguna | ○ Leve | ○ Moderada | ○ Intensa | ○ Muy intensa |

| 5. | En los últimos 7 días, ¿con qué FRECUENCIA tuvo VÓMITOS? | | | | |
| --- | --- | --- | --- | --- | --- |
|  | ○ Nunca | ○ Rara vez | ○ A veces | ○ A menudo | ○ Casi siempre |
|  | En los últimos 7 días, ¿cuál fue la INTENSIDAD de los VÓMITOS en su PEOR momento? | | | | |
|  | ○ Ninguna | ○ Leve | ○ Moderada | ○ Intensa | ○ Muy intensa |

| 6. | En los últimos 7 días, ¿con qué FRECUENCIA tuvo ACIDEZ ESTOMACAL? | | | | |
| --- | --- | --- | --- | --- | --- |
|  | ○ Nunca | ○ Rara vez | ○ A veces | ○ A menudo | ○ Casi siempre |
|  | En los últimos 7 días, ¿cuál fue la INTENSIDAD de la ACIDEZ ESTOMACAL en su PEOR momento? | | | | |
|  | ○ Ninguna | ○ Leve | ○ Moderada | ○ Intensa | ○ Muy intensa |

The PRO-CTCAE™ items and information herein were developed by the NATIONAL CANCER INSTITUTE at the NATIONAL INSTITUTES OF HEALTH, in Bethesda, Maryland, U.S.A. Use of the PRO-CTCAE™ is subject to NCI's Terms of Use.

**NCI PRO-CTCAETM Items - Spanish**

**Item Library Version 1.0**

| 7. | En los últimos 7 días, ¿con qué FRECUENCIA tuvo HINCHAZÓN DEL ABDOMEN (EN EL VIENTRE)? | | | | |
| --- | --- | --- | --- | --- | --- |
|  | ○ Nunca | ○ Rara vez | ○ A veces | ○ A menudo | ○ Casi siempre |
|  | En los últimos 7 días, ¿cuál fue la INTENSIDAD de la HINCHAZÓN DEL ABDOMEN (EN EL VIENTRE) en su PEOR momento? | | | | |
|  | ○ Ninguna | ○ Leve | ○ Moderada | ○ Intensa | ○ Muy intensa |

| 8. | En los últimos 7 días, ¿con qué FRECUENCIA tuvo HECES O EXCREMENTOS SUELTOS O LÍQUIDOS (DIARREA)? | | | | |
| --- | --- | --- | --- | --- | --- |
|  | ○ Nunca | ○ Rara vez | ○ A veces | ○ A menudo | ○ Casi siempre |

| 9. | En los últimos 7 días, ¿con qué FRECUENCIA tuvo DOLOR EN EL ABDOMEN (EL  VIENTRE)? | | | | |
| --- | --- | --- | --- | --- | --- |
|  | ○ Nunca | ○ Rara vez | ○ A veces | ○ A menudo | ○ Casi siempre |
|  | En los últimos 7 días, ¿cuál fue la INTENSIDAD del DOLOR EN EL ABDOMEN (EL VIENTRE) en su PEOR momento? | | | | |
|  | ○ Ninguna | ○ Leve | ○ Moderada | ○ Intensa | ○ Muy intensa |
|  | En los últimos 7 días, ¿cuánto INTERFIRIÓ el DOLOR EN EL ABDOMEN (EL VIENTRE) en sus actividades habituales o diarias? | | | | |
|  | ○ Nada | ○ Un poco | ○ Algo | ○ Muco | ○ Muchísimo |

| 10. | En los últimos 7 días, ¿cuál fue la INTENSIDAD de la PICAZÓN (COMEZÓN) EN LA PIEL en su PEOR momento? | | | | |
| --- | --- | --- | --- | --- | --- |
|  | ○ Ninguna | ○ Leve | ○ Moderada | ○ Intensa | ○ Muy intensa |

| 11. | En los últimos 7 días, ¿cuál fue la INTENSIDAD del ADORMECIMIENTO O DEL  HORMIGUEO EN LAS MANOS O EN LOS PIES en su PEOR momento? | | | | |
| --- | --- | --- | --- | --- | --- |
|  | ○ Ninguna | ○ Leve | ○ Moderada | ○ Intensa | ○ Muy intensa |
|  | En los últimos 7 días, ¿cuánto INTERFIRIERON el ADORMECIMIENTO O EL  HORMIGUEO EN LAS MANOS O EN LOS PIES en sus actividades habituales o diarias? | | | | |
|  | ○ Nada | ○ Un poco | ○ Algo | ○ Muco | ○ Muchísimo |

The PRO-CTCAE™ items and information herein were developed by the NATIONAL CANCER INSTITUTE at the NATIONAL INSTITUTES OF HEALTH, in Bethesda, Maryland, U.S.A. Use of the PRO-CTCAE™ is subject to NCI's Terms of Use.

**NCI PRO-CTCAETM Items - Spanish**

**Item Library Version 1.0**

| 12. | En los últimos 7 días, ¿con qué FRECUENCIA tuvo DOLOR? | | | | |
| --- | --- | --- | --- | --- | --- |
|  | ○ Nunca | ○ Rara vez | ○ A veces | ○ A menudo | ○ Casi siempre |
|  | En los últimos 7 días, ¿cuál fue la INTENSIDAD del DOLOR en su PEOR momento? | | | | |
|  | ○ Ninguna | ○ Leve | ○ Moderada | ○ Intensa | ○ Muy intensa |
|  | En los últimos 7 días, ¿cuánto INTERFIRIÓ el DOLOR en sus actividades habituales o diarias? | | | | |
|  | ○ Nada | ○ Un poco | ○ Algo | ○ Muco | ○ Muchísimo |

| 13. | En los últimos 7 días, ¿cuál fue la INTENSIDAD del AGOTAMIENTO, EL CANSANCIO O LA FALTA DE ENERGÍA en su PEOR momento? | | | | |
| --- | --- | --- | --- | --- | --- |
|  | ○ Ninguna | ○ Leve | ○ Moderada | ○ Intensa | ○ Muy intensa |
|  | En los últimos 7 días, ¿cuánto INTERFIRIERON EL AGOTAMIENTO, EL CANSANCIO O LA FALTA DE ENERGÍA en sus actividades habituales o diarias? | | | | |
|  | ○ Nada | ○ Un poco | ○ Algo | ○ Muco | ○ Muchísimo |

| 14. | En los últimos 7 días, ¿con qué FRECUENCIA SINTIÓ ANSIEDAD? | | | | |
| --- | --- | --- | --- | --- | --- |
|  | ○ Nunca | ○ Rara vez | ○ A veces | ○ A menudo | ○ Casi siempre |
|  | En los últimos 7 días, ¿cuál fue la INTENSIDAD de la ANSIEDAD en su PEOR  momento? | | | | |
|  | ○ Ninguna | ○ Leve | ○ Moderada | ○ Intensa | ○ Muy intensa |
|  | En los últimos 7 días, ¿cuánto INTERFIRIÓ la ANSIEDAD en sus actividades habituales o diarias? | | | | |
|  | ○ Nada | ○ Un poco | ○ Algo | ○ Muco | ○ Muchísimo |

| 15. | En los últimos 7 días, ¿con qué FRECUENCIA tuvo SENTIMIENTOS DE TRISTEZA O DE NO ESTAR FELIZ? | | | | |
| --- | --- | --- | --- | --- | --- |
|  | ○ Nunca | ○ Rara vez | ○ A veces | ○ A menudo | ○ Casi siempre |
|  | En los últimos 7 días, ¿cuál fue la INTENSIDAD de los SENTIMIENTOS DE TRISTEZA O DE NO ESTAR FELIZ en su PEOR momento? | | | | |
|  | ○ Ninguna | ○ Leve | ○ Moderada | ○ Intensa | ○ Muy intensa |
|  | En los últimos 7 días, ¿cuánto INTERFIRIERON los SENTIMIENTOS DE TRISTEZA O DE NO ESTAR FELIZ en sus actividades habituales o diarias? | | | | |
|  | ○ Nada | ○ Un poco | ○ Algo | ○ Muco | ○ Muchísimo |

The PRO-CTCAE™ items and information herein were developed by the NATIONAL CANCER INSTITUTE at the NATIONAL INSTITUTES OF HEALTH, in Bethesda, Maryland, U.S.A. Use of the PRO-CTCAE™ is subject to NCI's Terms of Use.

**NCI PRO-CTCAETM Items - Spanish**

**Item Library Version 1.0**

| ¿Presenta otros síntomas de los que desea informar? | |
| --- | --- |
| ○ Sí | ○ No |

Haga una lista de cualquier otro síntoma:

| 1. | En los últimos 7 días, ¿cuál fue la INTENSIDAD de este síntoma en su  PEOR momento? | | | | |
| --- | --- | --- | --- | --- | --- |
|  | ○ Ninguna | ○ Leve | ○ Moderada | ○ Intensa | ○ Muy intensa |
| 2. | En los últimos 7 días, ¿cuál fue la INTENSIDAD de este síntoma en su  PEOR momento? | | | | |
|  | ○ Ninguna | ○ Ninguna | ○ Ninguna | ○ Ninguna | ○ Ninguna |
| 3. | En los últimos 7 días, ¿cuál fue la INTENSIDAD de este síntoma en su  PEOR momento? | | | | |
|  | ○ Ninguna | ○ Ninguna | ○ Ninguna | ○ Ninguna | ○ Ninguna |
| 4. | En los últimos 7 días, ¿cuál fue la INTENSIDAD de este síntoma en su  PEOR momento? | | | | |
|  | ○ Ninguna | ○ Mild | ○ Moderate | ○ Severe | ○ Very severe |
| 5. | En los últimos 7 días, ¿cuál fue la INTENSIDAD de este síntoma en su  PEOR momento? | | | | |
|  | ○ Ninguna | ○ Ninguna | ○ Ninguna | ○ Ninguna | ○ Ninguna |

The PRO-CTCAE™ items and information herein were developed by the NATIONAL CANCER INSTITUTE at the NATIONAL INSTITUTES OF HEALTH, in Bethesda, Maryland, U.S.A. Use of the PRO-CTCAE™ is subject to NCI's Terms of Use.

# Appendix II: Definition Of Borderline Resectable Disease Table For [Section 3.2.1](#Sec3p2p1) (Eligibility)

Patient must have **1 or more** of the characteristics in the Column II to be eligible. If the patient has any characteristics in the Columns I (Less Extensive Disease) or III (More Extensive Disease), they are **NOT** eligible.

| **VESSEL** | **Column I:**  **POTENTIALLY RESECTABLE (NOT ELIGIBLE)** | **Column II:**  **BORDERLINE RESECTABLE (ELIGIBLE)** | **Column III:**  **UNRESECTABLE (NOT ELIGIBLE)** |
| --- | --- | --- | --- |
|  |  |  |  |
| SMV, PV | Interface < 180° and no occlusion | Interface ≥ 180° OR Short segment occlusion amenable to reconstruction | Occlusion not amenable to reconstruction |
| SMA, CA | No interface | Interface < 180° | Interface ≥ 180° |
| HA | No interface | Interface of any degree amenable to reconstruction | Interface not amenable to reconstruction |
| AORTA | Must have no involvement | Must have no involvement | Involvement |

# Appendix III: Patient Drug Information Handout and Wallet Card

**Information for Patients, Their Caregivers and Non-Study Healthcare Team on Possible Interactions with Other Drugs and Herbal Supplements**

The patient ____________________________ is enrolled on a clinical trial using the experimental study drug, **irinotecan***.* This clinical trial is sponsored by the National Cancer Institute. This form is addressed to the patient, but includes important information for others who care for this patient.

**These are the things that you as a healthcare provider need to know:**

**Irinotecan** interacts with a certain specific enzyme in the liver:

- The enzyme in question is CYP3A4. Irinotecan is broken down by this enzyme. The dose of irinotecan which the patient is taking assumes that these enzymes are working normally.

**To the patient: Take this paper with you to your medical appointments and keep the attached information card in your wallet**.

**Irinotecan** may interact with other drugs which can cause side effects. For this reason, it is very important to tell your study doctors of any medicines you are taking before you enroll onto this clinical trial. It is also very important to tell your doctors if you stop taking any regular medicines, or if you start taking a new medicine while you take part in this study. When you talk about your current medications with your doctors, include medicine you buy without a prescription (over-the-counter remedy), or any herbal supplements such as St. John’s Wort. It is helpful to bring your medication bottles or an updated medication list with you.

Many health care providers can write prescriptions. You must tell all of your health care providers (doctors, physician assistants, nurse practitioners, pharmacists) you are taking part in a clinical trial.

**These are the things that you and they need to know:**

**Irinotecan** must be used very carefully with other medicines that use the liver enzyme **CYP3A4**. Before you enroll onto the clinical trial, your study doctor will work with your regular health care providers to review any medicines and herbal supplements that are considered strong inducers/inhibitors of **CYP3A4*.***

- Please be very careful! Over-the-counter drugs (including herbal supplements) may contain ingredients that could interact with your study drug. Speak to your doctors or pharmacist to determine if there could be any side effects.
  - - Your regular health care provider should check a frequently updated medical reference or call your study doctor before prescribing any new medicine or discontinuing any medicine. Your study doctor’s name is

_____________________________________ and he or she can be contacted at

_____________________________________.

**Irinotecan** interacts with a specific liver enzyme called **CYP3A4** and must be used very carefully with other medicines that interact with **CYP3A4.**

- Before you enroll onto the clinical trial, your study doctor will work with your regular health care providers to review any medicines and herbal supplements that are considered strong inducers/inhibitors or substrates of CYPA4.
- Before prescribing new medicines, your regular health care providers should go to [a](http://medicine.iupui.edu/clinpharm/ddis/) frequently-updated medical reference for a list of drugs to avoid, or contact your study doctor.
- Your study doctor’s name is **_____________________________** and can be contacted at **_________________________________.**

**STUDY DRUG INFORMATION WALLET CARD**

You are enrolled on a clinical trial using the experimental study drug **irinotecan.** This clinical trial is sponsored by the NCI. **Irinotecan** may interact with drugs that are processed by your liver. Because of this, it is very important to:

- Tell your doctors if you stop taking any medicines or if you start taking any new medicines.
- Tell all of your health care providers (doctors, physician assistants, nurse practitioners, or pharmacists) that you are taking part in a clinical trial.
- Check with your doctor or pharmacist whenever you need to use an over-the-counter medicine or herbal supplement.


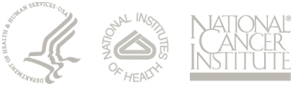


# Appendix IV: Standard lymphadenectomy Illustration

# Appendix V: Fiducial Marker Ordering Information

- **Gold Anchor: USA**

Naslund Medical Inc.

Fax: +1 (312) 277 6688

E-mail: order@FineNeedleMarker.com

- **Visicoil**:

BA Dosimetry

3150 Stage Post Drive - Suite 110

Bartlett, TN 38133 USA

E-mail: dosimetry-info@iba-group.com

Phone: +1 901 386 2242

Fax: +1 901 382 9453

- **Civco**

Toll-free within the U.S. & Canada: 800.842.8688

Direct Dial: 712.737.8688

Facsimile: 877.613.6300 or 319.248.4049

Direct Sales Email: roorder@civco.com

Distributor Sales Email: rodistorder@civco.com

OEM Email: rooemorder@civco.com
